# The accuracy of lung auscultation in the practice of physicians and medical students

**DOI:** 10.1371/journal.pone.0220606

**Published:** 2019-08-12

**Authors:** Honorata Hafke-Dys, Anna Bręborowicz, Paweł Kleka, Jędrzej Kociński, Adam Biniakowski

**Affiliations:** 1 Institute of Acoustics, Faculty of Physics, Adam Mickiewicz University in Poznań, Uniwersytetu Poznańskiego, Poland; 2 StethoMe, Winogrady, Poland; 3 Department of Pediatric Pneumonology, Allergology and Clinical Immunology, K. Jonscher Clinical Hospital in Poznań, Poznań University of Medical Sciences, Poland, Szpitalna, Poland; 4 Institute of Psychology, Adam Mickiewicz University, Wieniawskiego, Poland; University of Minnesota Masonic Children's Hospital, UNITED STATES

## Abstract

**Background:**

Auscultation is one of the first examinations that a patient is subjected to in a GP’s office, especially in relation to diseases of the respiratory system. However it is a highly subjective process and depends on the physician’s ability to interpret the sounds as determined by his/her psychoacoustical characteristics.

Here, we present a cross-sectional assessment of the skills of physicians of different specializations and medical students in the classification of respiratory sounds in children.

**Methods and findings:**

185 participants representing different medical specializations took part in the experiment. The experiment comprised 24 respiratory system auscultation sounds. The participants were tasked with listening to, and matching the sounds with provided descriptions of specific sound classes. The results revealed difficulties in both the recognition and description of respiratory sounds. The pulmonologist group was found to perform significantly better than other groups in terms of number of correct answers. We also found that performance significantly improved when similar sound classes were grouped together into wider, more general classes.

**Conclusions:**

These results confirm that ambiguous identification and interpretation of sounds in auscultation is a generic issue which should not be neglected as it can potentially lead to inaccurate diagnosis and mistreatment. Our results lend further support to the already widespread acknowledgment of the need to standardize the nomenclature of auscultation sounds (according to European Respiratory Society, International Lung Sounds Association and American Thoracic Society). In particular, our findings point towards important educational challenges in both theory (nomenclature) and practice (training).

## Introduction

Auscultation is a very common examination carried out by every general practitioner (GP) or family doctor. It is fast, easy and does not require advanced technology. Regardless of the type of stethoscope used (essentially a low cost device) the most important features of auscultation are non-invasiveness, simplicity and portability. However, the results of this kind of examination are subjective in nature, strongly dependent as they are on the experience and perceptual abilities of doctors and are hence prone to large errors. Indeed, physicians often differ in their assessments. Mangione and Nieman [1] examined the pulmonary auscultatory skills of medical students, pulmonologists and interns in internal medicine and family practice. They found that internal medicine and family practice doctors are not statistically superior to medical students. Only pulmonologists achieved statistically better results than others (e.g. for a diagnostic score: Cohen’s d = 1.16, p < .001;[1]).

Furthermore, discrepancies also arise from the differing nomenclature and formal distinctions between sound classes used in the existing medical literature. This is a wide ranging problem of international magnitude, as has been shown by numerous authors [2–5]. Pasterkamp et al. [6], proposed a unified nomenclature for six languages ​​and suggested a standardized terminology. However, there is still no uniformly standardized classification of the types of phenomena characteristic of the human respiratory system that is used by the medical community as a whole. Physicians often use words to describe perceived pathologies in the respiratory system that are associated with distinct semantic meanings in different handbooks or universities. This leads to problems in preliminary auscultation courses, and more importantly during later professional work, when doctors exchange or consult diagnoses. They use different descriptions of the same sounds or semantically similar and sometimes even identical terms to describe distinct phenomena. This results in descriptions that are ambiguous at the very least or in extreme cases even incomprehensible to other doctors, which in turn may require repetition of examinations to resolve. A further technical point is that the sounds are not stored and there is no possibility to go back to the recordings or compare them to other sounds, so there is also no possibility to verify the description, which increases the ambiguity even further.

## Study objectives

The main goal of this paper is to answer the following question: How correctly and consistently do physicians and medical students evaluate respiratory sounds, and do they categorize them identically? In this context, other aspects of the problem have also been analyzed in detail: Do pulmonologists perform better than other groups of physicians? How does the group of students fit into this community? These questions stem from the hypothesis that the reduction of pathological classes as proposed by Pasterkamp et al. [6], European Respiratory Society (ERS), International Lung Sounds Association (ILSA) or American Thoracic Society (ATS) may lead to an increase in efficiency of auscultation by medical staff. Particularly it means that even though physicians use the class of crepitus and medium crackles (see Table 1 for details) they cannot distinguished those classes from other crackles while listening and thus these terms should not be used. Moreover, we test the hypothesis that due to expert and daily practice, pulmonologists are likely to perform better than non-pulmonology medical providers and trainees.

**Table 1:** *The sound classes presented in the test*.

Sound class described by the team of medical specialists (the “standard”)	sound number in presentation
vesicular breath sound	1, 9, 21
normal bronchial sound	3, 16
abnormal bronchial sound	8
louder breath sound, prolonged expiratory phase and rhonchi	14, 24
fine crackles	4, 23
fine crackles and crepitus	13
fine crackles and abnormal bronchial sound	18
medium crackles	19
coarse crackles	5, 12
crepitus	22
rhonchi and expiratory wheezes	6
rhonchi	7
stridor	10
expiratory wheezes	11, 15, 20
inspiratory wheezes and rhonchi	17
inspiratory and expiratory wheezes	2

The investigated problem is especially crucial because most unwell patients, undergo examination by GPs and family doctors whose auscultation results are taken into account in more detailed diagnosis and further treatment [7].

## Materials and methods

The study was approved by the Bioethical Commission of the Poznań University of Medical Science.

Our test was anonymous and conducted online via the Internet using Questionpro Professional. This software was chosen because it enables the presentation of uncompressed and high quality audio to users. Moreover, before the experiment was made available to the participants, the quality of the signals in this software was subjectively verified by two experienced acousticians (sound engineers) independently (without hearing loss) for distortions and possible artifacts. No difference was found between direct and online listening. In addition, to minimize the possibility of a layperson completing the survey, the survey was distributed among the academic medical community and in hospitals. It was also passed on to interested people by both lecturers at medical universities and by practicing doctors.

The experiment consisted of two parts.

In the first part, a survey was used. Each participant responded to a number of questions regarding: (a) education, the specialization started or held, (b) the assessment of their own skills in adult and child auscultation, (c) the type of stethoscope (electronic / analog) used and the frequency of auscultation performed in their medical practice, (d) their opinions on a 5-grade scale on: the number of hours devoted to studying auscultation during their study and specialization, the need for additional training, and the scale of the problem of ambiguity in the nomenclature used in the classification of auscultation sounds.

The second part focused on the classification of auscultation sounds presented to each participant. This part consisted of 24 sounds (see Table 1 for details) that the test participant listened to, evaluated and assigned to specific classes (details below). Nine sounds were selected from the demonstration recordings included on the CD companion to Fundamentals of Lung and Heart Sounds [8]. The remaining sounds were recorded with a Littmann 3200 electronic stethoscope and consisted of the respiratory sounds of children aged 5 months to 14 years (average 7.6 years).

The choice of sounds for the test was conducted in two steps. First, sounds from a database of over 2000 sounds were selected by acousticians. The chosen sounds contained the least number of distortions and artifacts caused by the transducer of the stethoscope. The second equally important step was the choice of sound classes so as to have a pool of as diverse and unambiguous sounds as possible. In this way 50 different sound examples were selected. The final selection of sounds for the tests was made by a specialist team of eight experienced pediatricians and pulmonologists working at the Karol Jonscher Clinical Hospital in Poznań, Karol Marcinkowski Poznań University of Medical Sciences. At the meeting of these physicians, a Fostex PH-50 headset coupled with high quality professional headphones (Sennheiser HD600) enabled all the physicians to listen simultaneously to sound samples. Upon listening to each signal, they classified the sound. A common position was then reached after a discussion. The final set of sounds chosen for the test consisted of sounds that did not lead to any cause of doubt among the physicians. This set of signals along with their descriptions is called the “standard” here.

**Fig 1:**
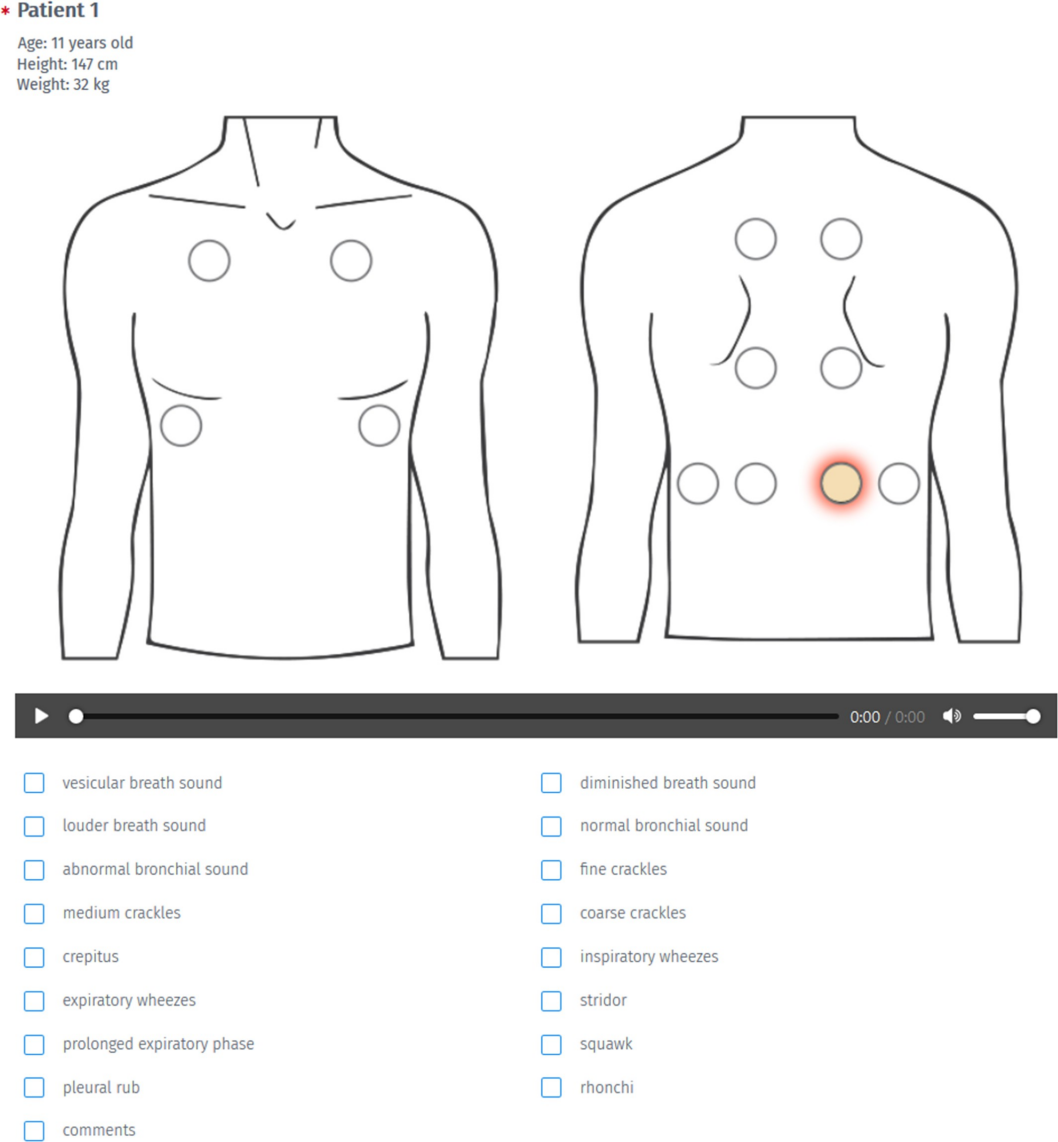
An example of the answer sheet used in the experiment and possible answers. During the experiment more than one class could be marked and each sound could be played as many times as needed.

The sounds represented certain classes (Table 1) and were presented to the participants as a collection of signals from 24 different patients. An exemplary answer sheet illustrating the scope of data presented to the participants is presented in Fig 1.

The terms used in the classes were chosen to include the entire spectrum of nomenclature that is used in contemporary medical literature [1, 4, 6, 8–11]. Some of the descriptions (e.g. squawk, diminished breath sound, pleural rub) did not correspond to any signals present in the recordings. During the experiment more than one class could be assigned to each sound by a listener. Each sound signal was supplemented with the following additional graphical and textual information: position where the stethoscope chestpiece was placed on the chest during the recording, and the age, height and weight of the patient (Fig 1). Each participant was informed of the need to use high quality headphones during the experiment. The participants were allowed to rerun each sound as many times as they needed and return to and modify previous descriptions. No feedback was used, *i*.*e*. the participants were not informed about the correctness of their response during the test. Moreover, the order of the presentation of the data was random (though identical for all participants). This was done to inhibit the possibility of systematic learning of patterns and hence correct answers by the participants during the listening task.

Due to the exploratory nature of the research, we assumed a type 1 error level of alpha = 0.10 for statistical hypothesis testing.

The survey was anonymous and voluntary. The participants were informed in the survey that their results would be analyzed and published. All the participants were over the age of 18 years. The recordings were collected in the Karol Jonscher Clinical Hospital in Poznań, Poznań University of Medical Sciences by physicians from the Department of Pediatric Pneumonology, Allergology and Clinical Immunology, with the consent of the Bioethical Commission of the Poznań University of Medical Sciences. Also, the legal guardians of children taking part were required to sign written permission for the recordings to be made.

Both parts of the experiment (survey and descriptions) were completed by 205 participants. Firstly, the results from the questionnaires were analyzed for their quality. The average time taken to complete the questionnaires was 25 min. The total duration of the signals, excluding the survey part, was about 4 minutes. Therefore, the responses of the participants who completed the entire experiment in less than 5 minutes were rejected as unreliable, and the remaining 185 questionnaires were approved for further analysis. Among these, there were: 16 pulmonologists, 22 pediatricians, 29 doctors of other specializations, 50 doctors during their internship and 68 medical students.

## Results

**Fig 2:**
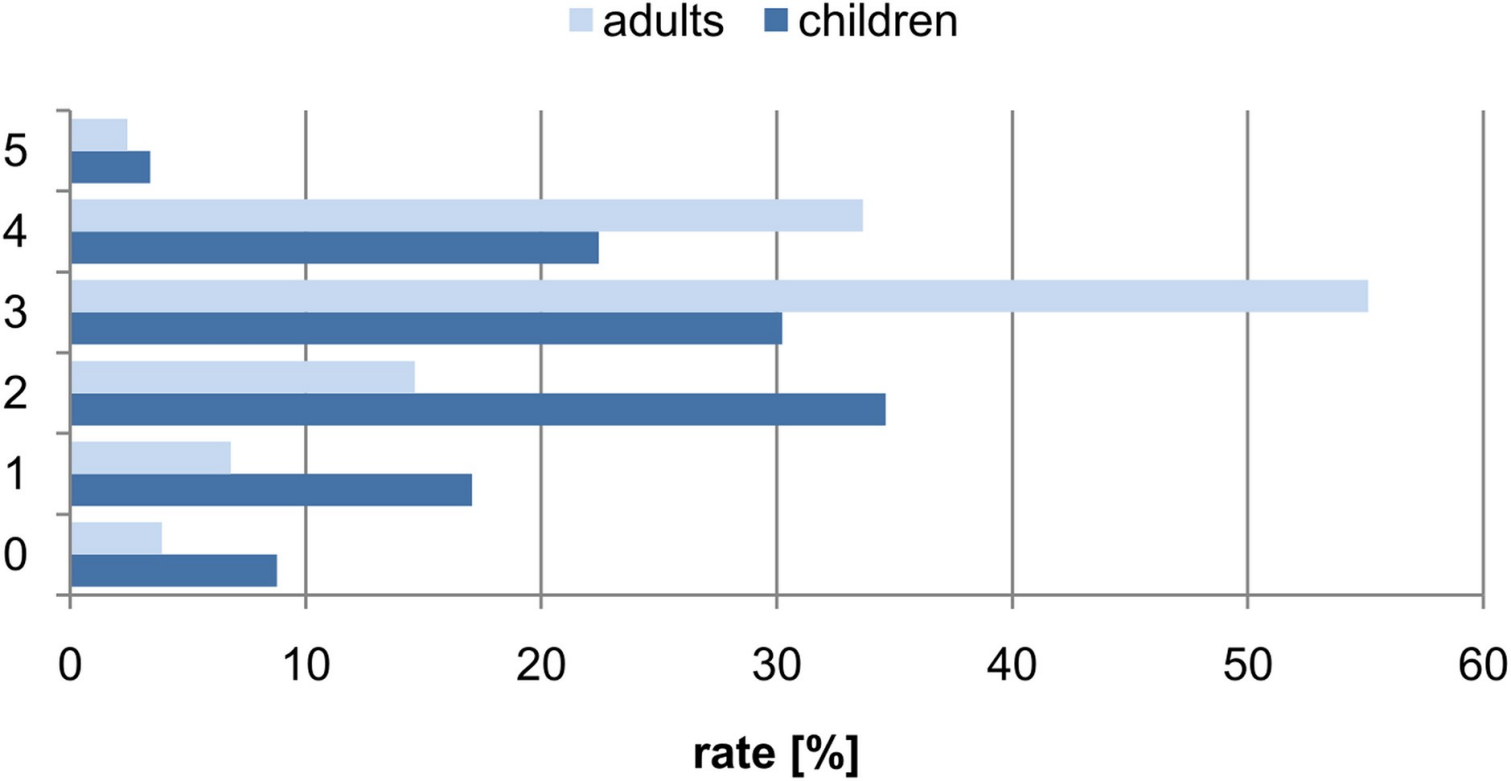
The percentage distribution of responses to the question of assessing one's own skills (on a scale of 0-"very poorly" to 5 = "very well") for the auscultation of adult and child respiratory systems.

Among the respondents, the majority (67%) auscultate patients at least once a week, while 14% do it at least once a month. The remaining group auscultate their patients less than once a month (19%). Only 4.9% of the surveyed physicians use an electronic stethoscope in their daily practice. Fig 2 shows how the physicians rated their skills in the auscultation of children and adults.

**Fig 3:**
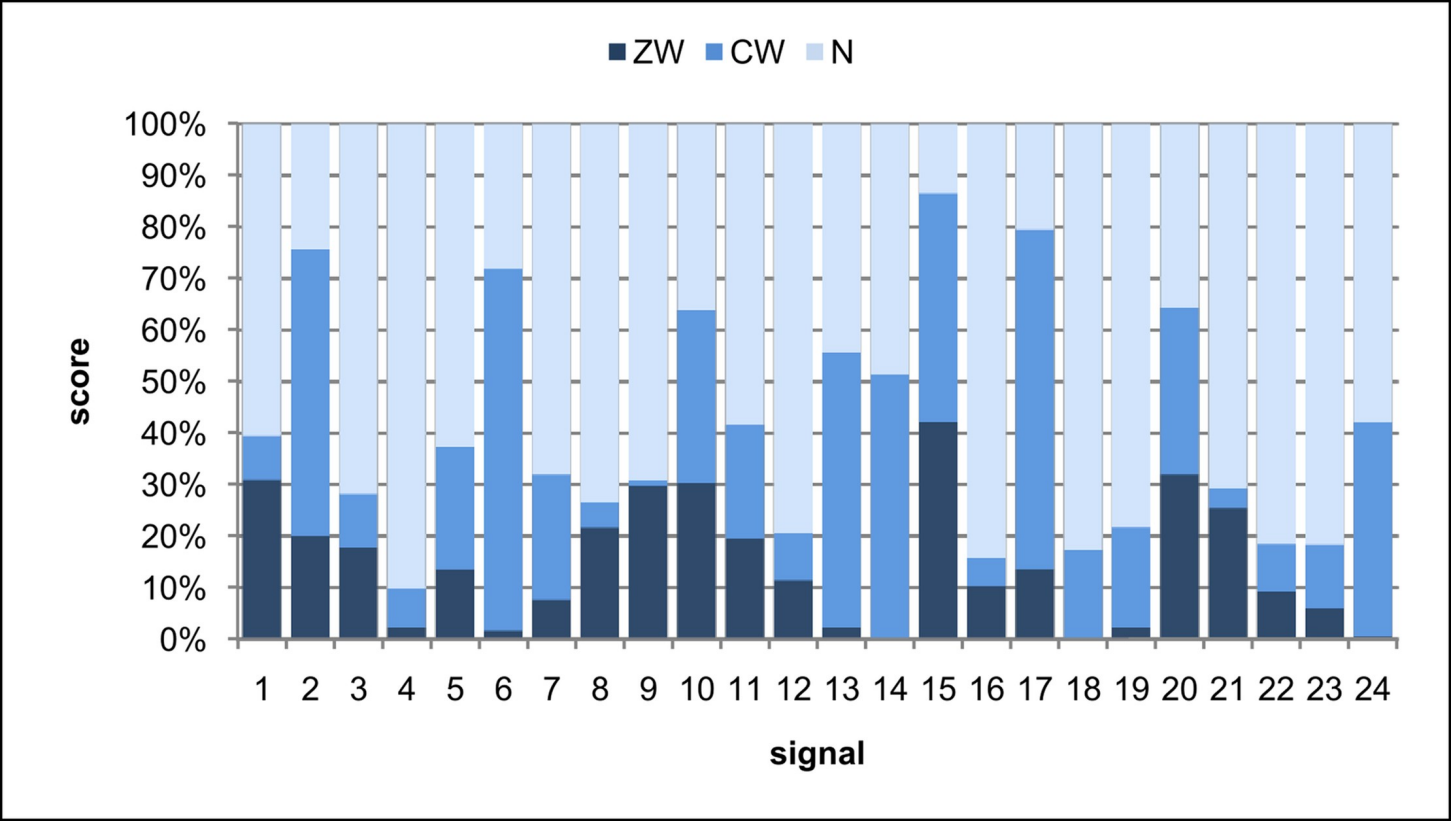
The distribution of answers that are in: full agreement with the “standard”(ZW), partial agreement with the “standard” (CW) and incorrect (N).

The results of the auscultation experiment were divided according to the correspondence of sound classification carried out by the participants with the “standard” developed by the team of medical specialists (Table 1). For each sound presented to the participants, the responses were categorized as in Table 2 and the percentage distribution of each such category is shown in Fig 3.

**Table 2:** Assumptions used to evaluate the responses of participants and the abbreviation used.

abbreviation	response category	description of category
ZW	the answer was in agreement with the standard	full agreement with the “standard” and no incorrect answers
CW	the answer was partially in agreement with the standard	at least one correct answer marked; additional phenomena that were not included in the “standard” were marked
N	incorrect answer	among the marked phenomena, the response from the “standard” was not marked

**Fig 4:**
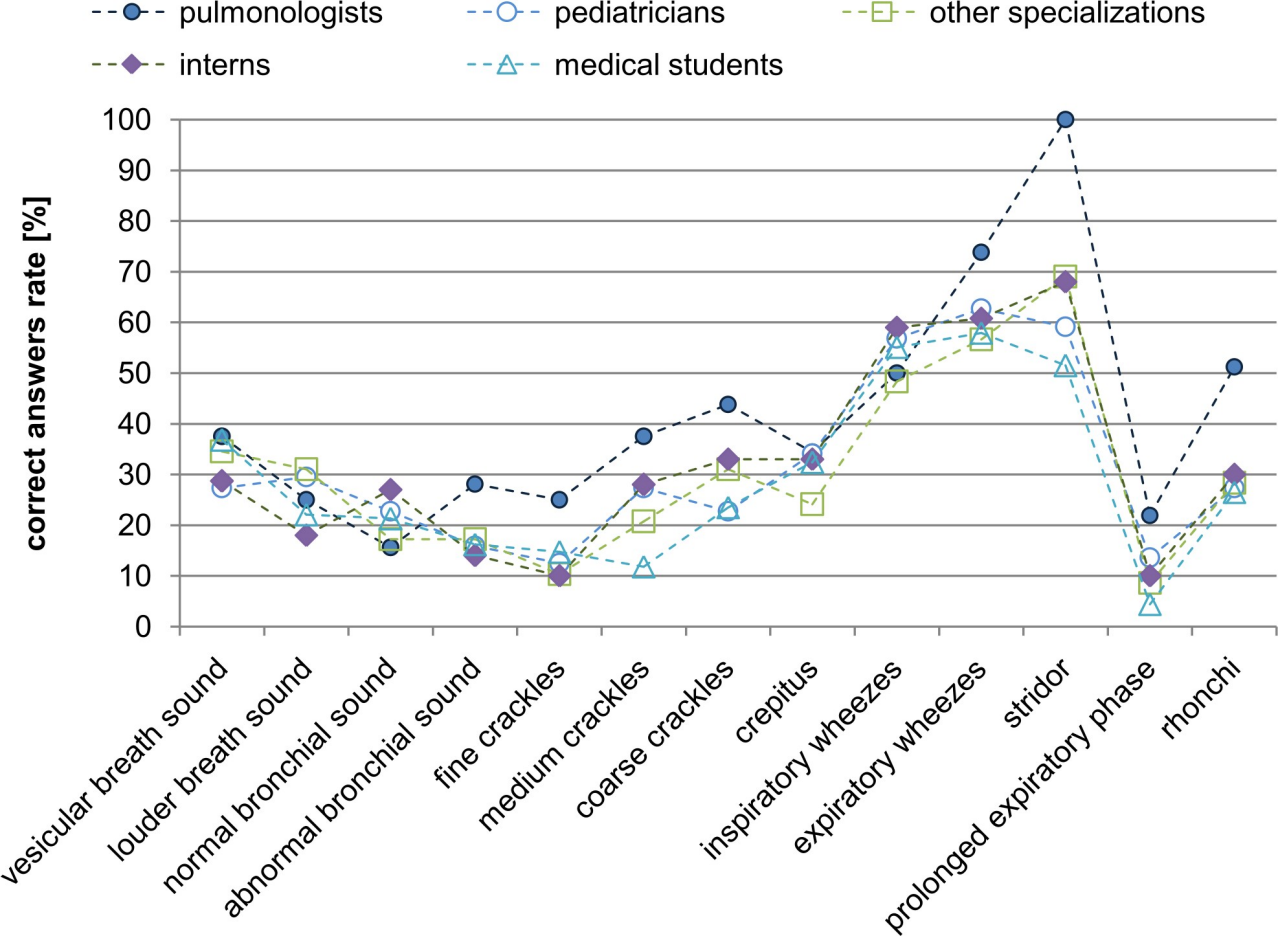
The juxtaposition of correct (P = ZW∪CW) answers for each sound class for doctors of different specializations and medical students.

Analyzing all the data presented up to now, one may infer that doctors classify sounds with varying accuracy (Fig 3). In general, after averaging the responses for all the sounds we find that there are 14.6% of ZW answers (full agreement with the “standard”), 26.2% of CW answers (partial agreement with the “standard”, see Table 2), while most answers are of type N (incorrect answers) - 59.3% (chi2(2) = 59.8, p <0.001). Furthermore, it can be seen by conducting the Kruskal-Wallis test that there are statistical differences between the groups of physicians of different specializations in the category of correct answers ZW (chi2(4) = 16.0, p < 0.003). Pair analysis of the differences in answers between groups of physicians of different specializations using post hoc tests with a Holm correction show that pulmonologists have statistically higher scores (15.9%) than students (14.4%, p = 0.023) and interns (13.8%, p = 0.060). This analysis also shows that students had significantly lower scores in CW’s (24.1%) than pulmonologists (36.4%, p = 0.022), pediatricians (26.7%, p = 0.022) and other specializations (24.6%, p = 0.073). With regard to incorrect answers (N), students obtained the most (61.5%), while pulmonologists obtained the least (47.7%) (chi2 (4) = 69.9, p <0.001). Next, the differences for individual sound classes (Fig 4) are analyzed on the basis of a Kruskal-Wallis test and post hoc tests with a Holm correction for statistically significant differences. According to the analysis, pulmonologists distinguish the sound classes better than other groups—as many as three classes are significantly recognized differently by the group of pulmonologists relative to other specializations: fine crackles (chi2(4) = 11.9, p = 0.018), stridor (chi2 = 14.4, p = 0.006) and rhonchi (chi2 (4) = 11.1, p = 0.026).

For further analysis, the sounds were grouped according to their class and the percentage of correct responses (P), and considered for each medical specialization separately (Fig 4). To give a general view, we assumed the correct answer category (P) to contain both the responses in full (ZW) and partial (CW) agreement with the “standard”.

Next, we grouped the responses using the main classes proposed by ERS [6], ILSA or ATS. In practice, this means that if the participant had indicated any of the subgroups shown in Table 3 and any of them were marked as correct in the “standard”, the answer was treated as a correct one. The cases of the vesicular breath and bronchical sounds, are each treated as standalone sounds. In this way, five main classes of sounds were created (Table 3).

**Table 3:** The main classes of respiratory sounds and the subclasses that are part of them.

Main class	subclasses included in the main class
normal lung sound	vesicular breath sound diminished breath sound louder breath sound normal bronchial sound
abnormal bronchial sound	no subclasses
crackles	fine crackles medium crackles coarse crackles crepitus
wheezes	inspiratory wheezes expiratory wheezes stridor
rhonchi	no subclasses

**Fig 5:**
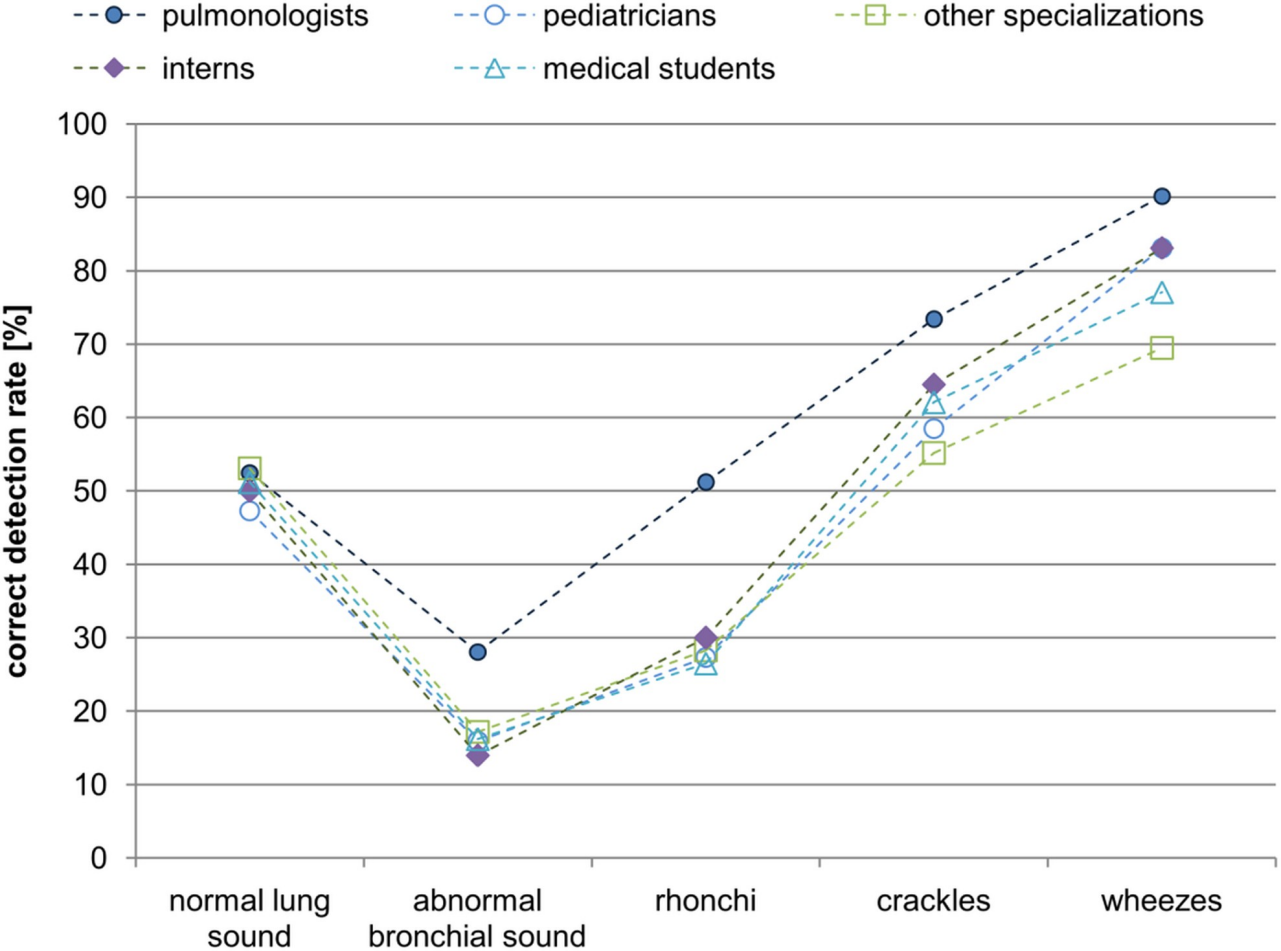
The percentage of correct detection of the grouped sound classes (main classes, Table 3) by physicians of various specializations. To emphasize the differences, the points for individual groups are connected by lines.

After grouping the responses as in Table 3, for all the classes associated with pathological signals, the pulmonologist group is still seen to be the most effective one (Fig 5). However, statistically significant differences based on the Kruskal-Wallis test and a comparison of the groups of participants in pairs are obtained only for the rhonchi class between the group of pulmonologists who scored higher than the pediatricians (p = 0.085), interns (p = 0.085), other specializations (p = 0.046) and medical students (p = 0.015). For wheezes, pulmonologists had statistically significantly higher scores than other specializations (p = 0.008). Also interns had better scores than physicians of other specializations (p = 0.026). Generally,we find that that this grouping highlights the advantage of pulmonologists over the rest of the groups in the correct recognition of phenomena related to respiratory sounds.

The lowest value of false interpretation appears for the wheezes class. This suggests that this class is the easiest to recognize because the acoustically distinctive features contained in the signal are easy to detect and interpret. There was no difference in the results for the different groups of physicians: normal lung sound (chi2 (4) = 2.4, p = 0.671); abnormal bronchial sound (chi2 (4) = 4.5, p = 0.348); rhonchi (chi2 (4) = 2.1, p = 0.722); crackles (chi2 (4) = 1.0, p = 0.908); wheezes (chi2 (4) = 7.0, p = 0.135).

A graph of the correlation between the correct answers for each main class is presented using the the described grouping (Fig 5). For each group of participants the percentage of false positives is about 50% and decreases to 30% for wheezes only.

**Fig 6:**
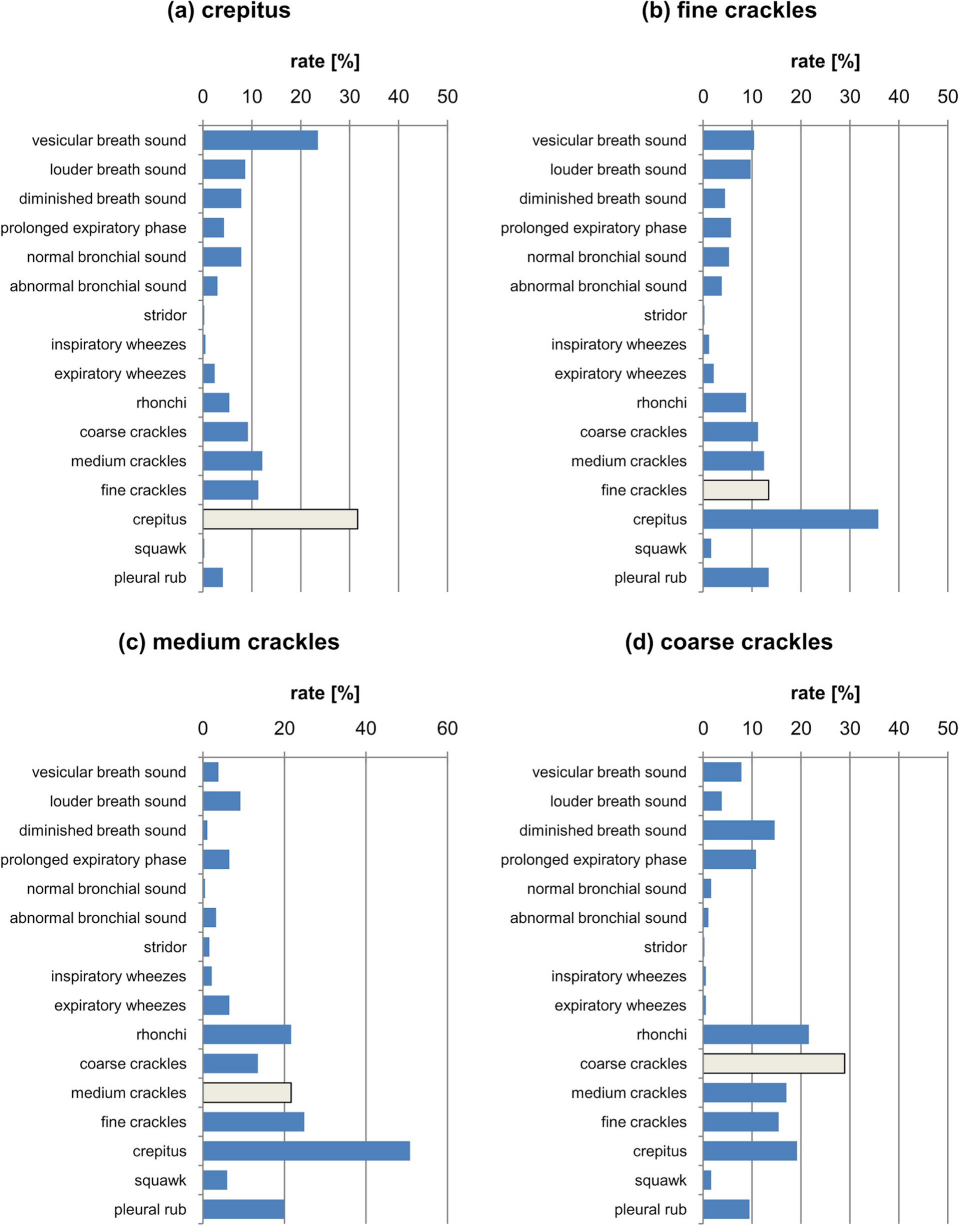
The percentage distribution of responses marked for the classes (a) crepitus (b) fine crackles (c) medium crackles (d) coarse crackles. The light grey bar depicts the correct answer.

**Fig 7:**
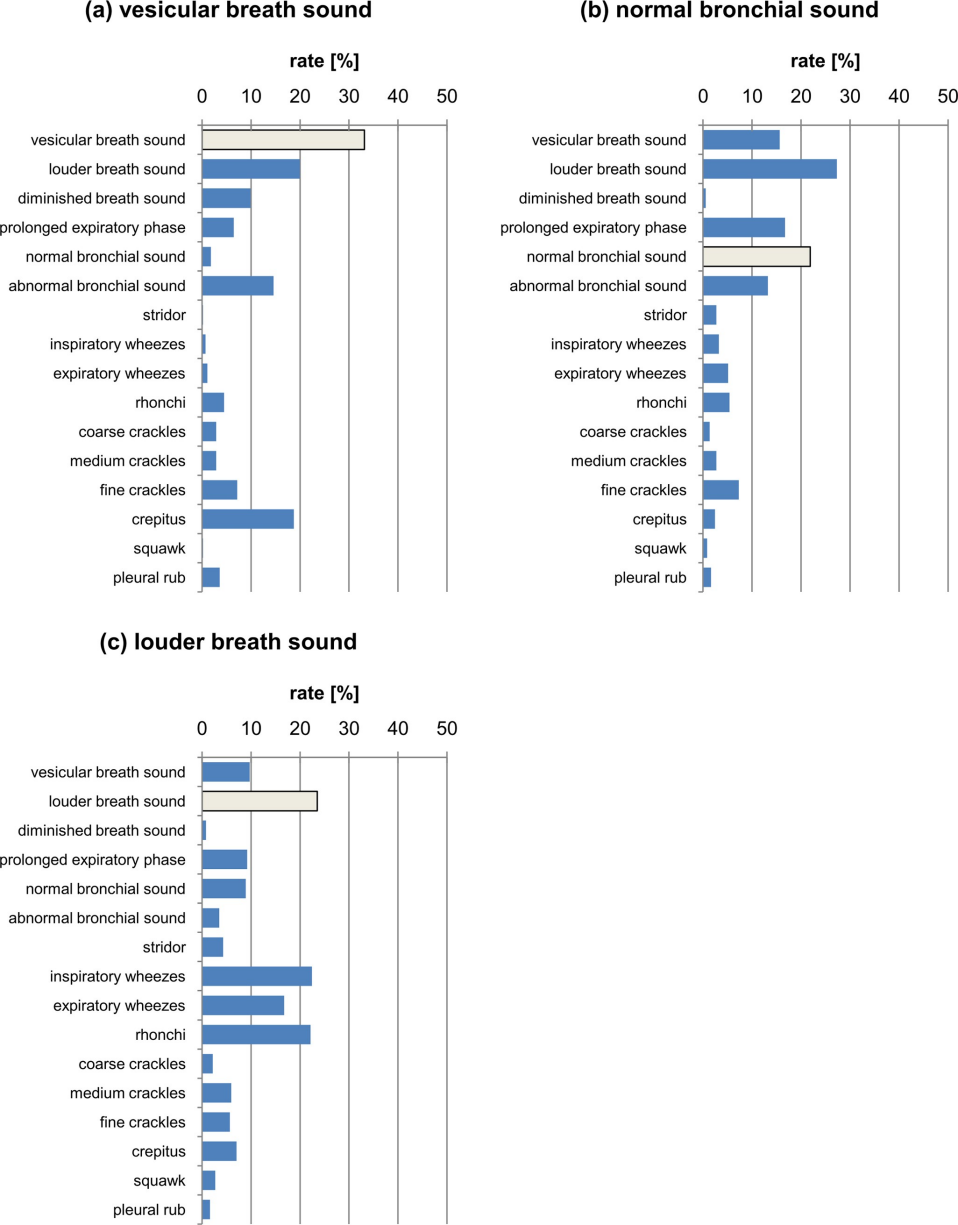
The percentage distribution of responses marked for the classes (a) vesicular breath sound; (b) normal bronchial sound; (c) louder breath sound. The light grey bar depicts the correct answer.

**Fig 8:**
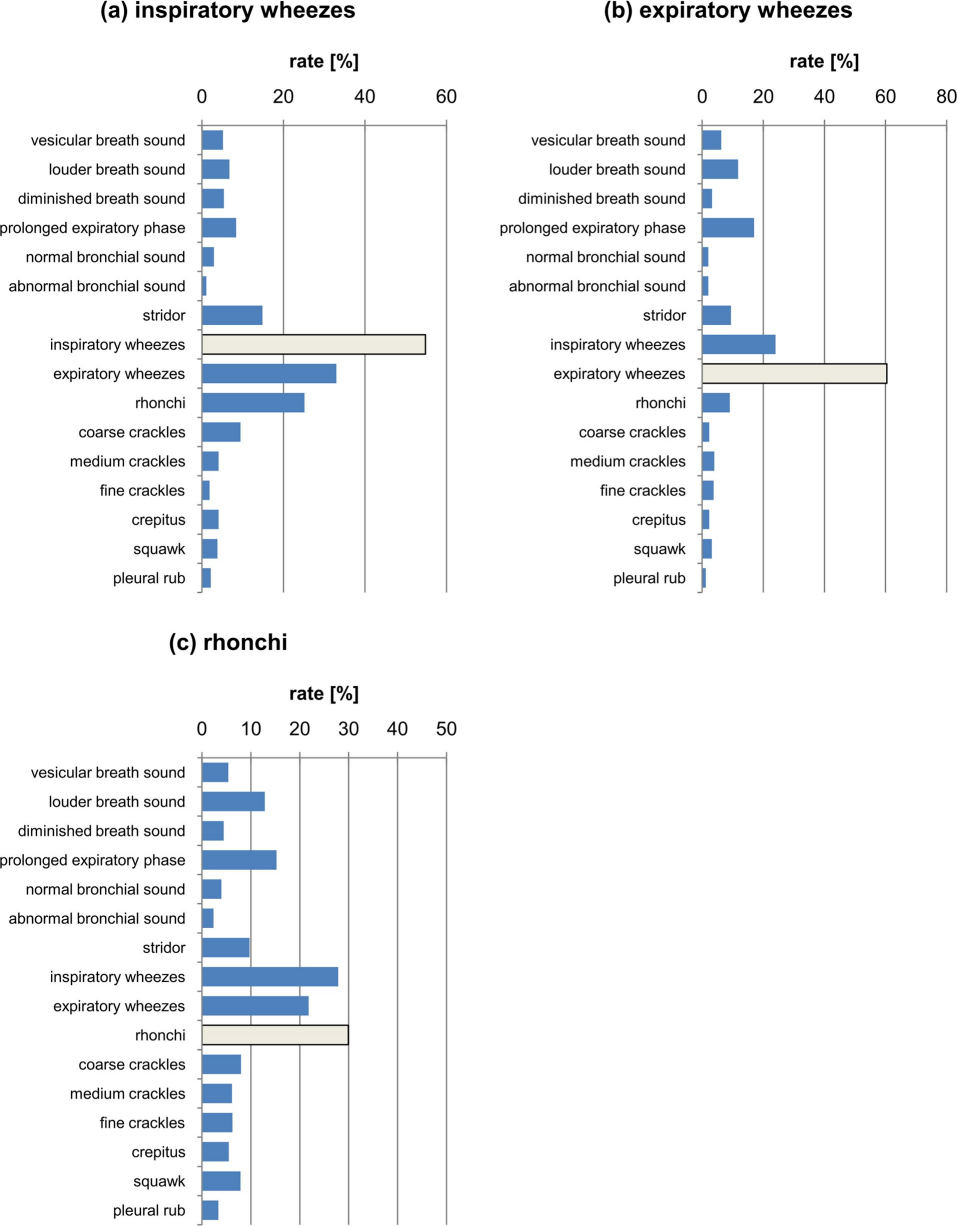
The percentage distribution of responses marked for (a) inspiratory wheezes, (b) expiratory wheezes, (c) rhonchi class. The light grey bar depicts the correct answer.

Furthermore, we analyzed what phenomena other than corresponding to the “standard” were marked by the participants. It should be emphasized that the purpose of this analysis was primarily to investigate inter-relations between groups of classes which is important in the context of further future unification of the existing nomenclature. We assumed that phenomena that are most similar would also most often be confused with each other and hence should be grouped together giving rise to the the following coarse-grained classes: the crackles class (Fig 6), breath sounds (vesicular and louder) and bronchial sounds (Fig 7), wheezes and rhonchi class (Fig 8). Each plot shows all responses, where specifically the correct answer is depicted in grey. In this way, one can analyse which classes were chosen by the respondents and the kind and frequency of the mistakes made.

Finally, in the test, both the reference sounds included on the CD of the physician handbook [8] and real-life sounds recorded using the Littmann 3200 stethoscope were used. The effect of the type of sound recording on the results was analyzed, taking into account the specialization of the participants. On the basis of the results of the Wilcoxon test, no significant difference in the percentage of any type of diagnoses between the two kinds of recording (CD and Littmann 3200) was observed in any of the participants (p>0.10).

## Discussion

It should be emphasized that the medical community recognizes the problem of the ambiguous nomenclature used in the classification of respiratory sounds. In our test, more than 65% of respondents strongly agree or agree, and only 14.2% disagree or strongly disagree with the statement that the classification of respiratory sounds requires coherence and uniformity.

Analysing different sources of recordings one may state that that the type of recording did not affect the results of participants and that they performed at a similar level at the task of classification of sounds irrespective of of different origins (from a book or from a hospital). It means that the results can be generalized and are not affected by the user or device. Therefore they can be analyzed together.

Analysing the results corresponding to the juxtaposition of coarse, medium, fine crackles and crepitus (Fig 6), it can be seen that these are the classes that are confused with each other. Coarse crackles are most often confused with other types of crackles and crepitus, and even rhonchi. In the medium crackle class the results are similar to those for coarse crackles, but the most noticeable additional class marked for this phenomenon was crepitus.

In turn, fine crackles are not confused so often with rhonchi and medium and coarse crackles, but are often confused with the crepitus category, which is a class that is marked much more often than in the standard (fine crackles). This state of affairs seems natural, because in many medical communities crepitus is equivalent to fine crackles, or is a subgroup of this class [11]. This findings lead to the conclusion that only two classes for these phenomena should be distinguished, namely fine and coarse classes. Nevertheless, in the case of crepitus, the most common category marked was vesicular breath sound, which may mean that the respondents did not notice the sound class and treated the extra sound as an artifact occurring during the recording. This is confirmed by Fig 7A, in which the answers differing from the correct ones (vesicular breath sound) are shown. Note that the respondents, in addition to the correct answer, most often chose a louder breath sound and crepitus. In the case of normal bronchial sound (Fig 7B), the louder breath sound was more frequent than the correct answer. In addition, the respondents also often noted a prolonged expiration phase.

In samples consisting of the louder breath sound (Fig 7C), the subjects often recognized the three respiratory pathology classes—inspiratory and expiratory wheezes or rhonchi. In the case of normal sounds, these classes were never selected.

In the case of the juxtaposition of inspiratory and expiratory wheezes (Fig 8), notice that they are generally recognized correctly, as witnessed by the highest percentage of correct answers provided. These two classes were also confused most frequently, i.e. inspiratory wheezes were confused with expiratory wheezes and vice versa. In addition, only in the case of inspiratory wheezes was rhonchi marked as an additional class. This means that distinguishing the inspiratory and expiratory phases poses significant difficulty to physicians.

In the case of rhonchi (Fig 8C), notice that this is the class closest to wheezes and is most often confused with them. The class of inspiratory and expiratory wheezes is marked almost as often as the rhonchi class, which was the correct answer here. Additionally, for the rhonchi class a louder breath sound and prolonged expiration phase were also often marked.

In general, comparing the results in Fig 8A and 8C, we find that the wheezes and rhonchi group are subjectively close to each other and are often confused due to similar acoustic features. Fig 8C shows that in sound samples which contained rhonchi, according to the “standard”, the respondents mainly mistook them for the class of wheezes. Moreover, the class of rhonchi was also often chosen (Fig 6) when the standard response was medium or coarse crackles. We infer that the rhonchi class possesses the acoustic features characteristic for the crackles class, causing confusion among the participants. Rhonchi, like wheezes, are continuous and periodic sounds. However, their fundamental frequency is lower than that of wheezes. They are often mistaken for them. This is consistent with the results obtained by Willkins et al. [4], who also showed that rhonchi are an ambiguous class that are very often incorrectly identified. In conclusion, these results point towards the fact that the class of rhonchi is quite ambiguous and is differently classified by the respondents due to the fact that it has the features of both wheezes and crackles.

The comparison of the results of correct answers for the detailed classification and the classification with the grouped (main) classes shows that grouping according to ERS, ILSA and ATS nomenclature significantly increases the percentage of correct responses. This is also consistent with the research by Malbye et.al. [12] and emphasized in handbooks [13].

The comparison of the efficiency of different groups of medical staff shown in Fig 5 reveals that pulmonologists perform better than other groups- this is also consistent with the results of Mangione and Nieman [1]. Their advantage is the greatest for the rhonchi class. Interns and pediatricians perform next best followed by medical students and other specializations. We note here, that the former two classes of physicians use stethoscopes in their everyday practice similarly to pulmonologists. Furthermore physicians (except for pulmonologists), in general, are not better than medical students. It must be emphasized that some authors (e.g. [14]) do not find pulmonologists to have an advantage.

## Conclusions

The main findings of this study are collected below:

- The average number of correct detections of auscultation phenomena in our test using the wide detailed nomenclature used in the medical community ranges from from only 24.1% for students to 36.5% for pulmonologists.
- The highest number of correct answers was obtained for the wheeze classes. This is not surprising, since they are the most characteristic sounds: continuous, tonal, periodic and relatively loud. For coarse and fine crackle classes, the number of correct detections was lower.
- The most ambiguous is the class of rhonchi, which is at the boundary between the class of wheezes and the class of coarse crackles.
- Our results lend support to the use of the classification of ERS [6], ILSA or ATS which is simple, short, well defined acoustically, and do not imply any wrong mechanism of production. On this basis one can distinguish two main categories of sounds: discontinuous sounds (which are divided into classes of fine and coarse crackles), and continuous sounds (wheezes and rhonchi). These four classes seem to be enough since more detailed ones cannot be correctly distinguished by physicians.
- For all classes of sounds, the advantage of the group of pulmonologists in terms of their correct answers is evident in our results.The effectiveness of physicians in the clear classification of auscultation sounds with the use of detailed sound classes is very heterogeneous and does not bring any advantage over general classes.
- Due to the ever-growing market of electronic stethoscopes, it is possible to record normal and pathological respiratory sounds. It is therefore possible to create large sound databases that can be subsequently described by experts in the field of pulmonology. Such databases, consisting of sound examples with descriptions and attributed sound classes, can be used to train medical students and improve the competence of medical practitioners of various specialities.

## Supporting information

S1 Table Raw data table. According to Table 1 in the paper.
(PDF)

S1 File Survey template in Polish. (PDF)

S2 File Survey template in English. (PDF)
