# Supplementary material for: The accuracy of lung auscultation in the practice of physicians and medical students
Source: PLoS One. 2019 Aug 12;14(8):e0220606. doi: 10.1371/journal.pone.0220606 (PMC6690530; doi:10.1371/journal.pone.0220606)
Supplement: S1 Table — According to Table 1 in the paper. (PDF) [file pone.0220606.s001.pdf]

			See Table 1 for details			1	1	1	1	1	1	1	1	1	
response_id	time_taken	specialization	year of graduation (prediction for students)	skills assessment in children auscultation	skills assessment in adults auscultation	1	2	3	4	5	6	7	8	9	[illegible]
60925047	1893	pulmonologist	1995.0	6.0	5.0	1	0	0	0	0	0	0	0	0	[illegible]
60930255	3652	pulmonologist	2003.0	5.0	4.0	0	0	0	0	0	1	0	0	0	[illegible]
60932321	2487	pulmonologist	2005.0	5.0	3.0	0	0	0	0	0	1	0	0	0	[illegible]
60987975	1306	pediatrician	2011.0	5.0	4.0	0	0	0	0	1	0	0	0	0	[illegible]
60989281	2532	pulmonologist	2006.0	4.0	5.0	0	0	0	0	1	0	0	0	0	[illegible]
60992594	4784	pulmonologist	1996.0	5.0	4.0	0	1	0	0	0	0	0	0	0	[illegible]
61043414	2622	pediatrician	2003.0	5.0	5.0	0	0	0	0	0	0	0	0	0	[illegible]
61043560	3783	pulmonologist	2004.0	5.0	3.0	0	0	1	0	0	0	0	0	1	[illegible]
61048793	3660	pulmonologist	1998.0	5.0	4.0	1	0	0	0	0	0	0	0	0	[illegible]
61052046	5266	pulmonologist	1996.0	5.0	4.0	1	0	0	0	0	0	0	0	0	[illegible]
61058037	2421	other	1995.0	6.0	5.0	0	0	0	0	0	0	0	0	1	[illegible]
61058319	4206	pulmonologist	1990.0	5.0	5.0	0	0	1	0	0	0	0	0	1	[illegible]
61061881	4109	pulmonologist	1995.0	5.0	5.0	1	0	0	0	0	0	0	0	0	[illegible]
61064314	4236	pulmonologist	1994.0	5.0	4.0	0	0	1	0	0	1	0	0	0	[illegible]
61088645	858	pulmonologist	2002.0	5.0	3.0	0	0	1	0	0	0	0	0	0	[illegible]
61094756	1114	pulmonologist	2001.0	6.0	4.0	1	0	0	0	0	0	0	0	0	[illegible]
61097671	2498	pediatrician	2005.0	5.0	3.0	0	0	1	0	0	0	0	0	1	[illegible]
61105871	2958	pediatrician	2001.0	5.0	3.0	1	0	0	0	0	0	0	0	0	[illegible]
61182117	1788	pulmonologist	1993.0	5.0	3.0	0	0	0	0	0	0	0	0	1	[illegible]
61459345	1125	pediatrician	2013.0	4.0	4.0	1	0	0	0	0	0	0	0	0	[illegible]
61461061	1374	pediatrician	2009.0	6.0	3.0	0	0	0	0	1	0	0	0	0	[illegible]
61653537	701	intern	2014.0	4.0	5.0	0	0	0	0	0	0	0	0	1	[illegible]
61663996	2057	pediatrician	2015.0	4.0	4.0	0	0	0	0	1	0	0	0	1	[illegible]
61727131	841	pediatrician	2014.0	5.0	4.0	1	0	0	0	0	0	0	0	0	[illegible]
61743507	4551	student	2017.0	4.0	4.0	1	0	0	0	0	0	0	0	0	[illegible]
61744463	940	student	2018.0	2.0	4.0	1	0	0	0	0	0	0	0	0	[illegible]
61744998	798	student	2018.0	4.0	4.0	0	0	0	0	0	0	0	0	1	[illegible]
61746641	1154	student	2020.0	4.0	4.0	0	0	0	0	1	0	1	0	1	[illegible]
61747621	1217	intern	2015.0	4.0	5.0	1	0	0	0	0	0	0	0	0	[illegible]
61748199	5880	student	2017.0	3.0	4.0	0	0	1	0	0	0	0	0	0	[illegible]
61758762	1373	student	2020.0	2.0	2.0	1	0	0	0	0	0	0	0	0	[illegible]
61782611	1422	student	2017.0	1.0	2.0	0	0	1	0	0	0	0	0	0	[illegible]
61782724	688	student	2017.0	4.0	5.0	0	0	0	0	0	0	0	0	1	[illegible]
61782793	792	intern	2016.0	3.0	2.0	1	0	0	0	0	0	0	0	1	[illegible]
61782870	645	intern	2015.0	4.0	3.0	0	0	0	0	1	0	0	1	0	[illegible]
61782877	970	student	2020.0	4.0	4.0	1	0	0	0	0	0	0	0	0	[illegible]
61783036	778	student	2018.0	3.0	4.0	0	0	1	0	0	0	0	0	0	[illegible]
61783244	1337	intern	2016.0	4.0	5.0	0	0	1	0	0	0	0	0	1	[illegible]
61783349	795	student	2017.0	3.0	4.0	0	0	1	0	0	0	0	0	1	[illegible]
61783370	724	student	2020.0	1.0	1.0	1	0	0	0	0	0	0	0	0	[illegible]
61783382	825	student	2017.0	3.0	4.0	0	0	1	0	0	0	0	0	1	[illegible]
61783501	966	intern	2015.0	3.0	5.0	0	0	0	0	0	0	0	0	1	[illegible]
61783701	1404	intern	2016.0	4.0	4.0	1	0	0	0	0	0	0	0	0	[illegible]
61783970	1280	intern	2014.0	4.0	4.0	0	0	0	0	0	1	0	0	0	[illegible]
61784599	1642	other	2014.0	3.0	4.0	1	0	0	0	0	0	0	0	0	[illegible]
61784643	657	student	2017.0	3.0	3.0	0	0	0	0	0	0	0	0	1	[illegible]
61784702	706	pediatrician	2016.0	3.0	3.0	1	0	0	0	0	0	0	0	0	[illegible]
61784914	787	student	2020.0	2.0	4.0	0	0	0	0	1	0	0	0	0	[illegible]
61785874	927	other	2015.0	4.0	5.0	1	0	0	0	0	0	0	0	0	[illegible]
61786207	1621	intern	2016.0	3.0	4.0	1	0	0	0	0	0	0	0	1	[illegible]
61786383	895	intern	2013.0	5.0	3.0	0	0	1	0	0	0	0	0	1	[illegible]
61786470	1507	intern	2016.0	1.0	2.0	1	0	0	0	1	0	0	0	1	[illegible]
61786592	1382	other	2014.0	5.0	5.0	1	0	0	0	0	0	0	0	0	[illegible]
61786886	1358	pediatrician	2014.0	4.0	4.0	0	0	0	0	0	0	0	0	1	[illegible]
61787029	837	intern	2016.0	3.0	4.0	0	0	0	0	0	1	0	0	0	[illegible]
61787669	986	other	2016.0	3.0	4.0	0	0	0	0	1	0	0	0	0	[illegible]
61788150	1678	pulmonologist	2015.0	4.0	5.0	0	1	0	0	0	0	0	0	1	[illegible]
61788217	1598	other	2013.0	3.0	4.0	1	0	0	0	0	0	0	0	0	[illegible]
61788610	901	student	2019.0	4.0	5.0	1	0	0	0	0	0	0	0	1	[illegible]
61788764	801	intern	2015.0	3.0	5.0	0	0	0	0	1	0	0	0	1	[illegible]
61788876	1845	intern	2016.0	4.0	4.0	1	0	0	0	0	0	0	0	0	[illegible]
61788991	1632	pediatrician	2015.0	3.0	4.0	0	0	0	0	1	0	0	0	0	[illegible]
61789008	1271	student	2019.0	4.0	5.0	1	0	0	0	1	0	0	0	0	[illegible]
61789058	948	other	2015.0	1.0	5.0	0	0	1	0	0	0	0	0	0	[illegible]
61789566	1062	student	2020.0	1.0	1.0	1	0	0	0	0	0	0	0	0	[illegible]
61789639	1404	intern	2016.0	4.0	5.0	0	0	1	0	0	0	0	0	1	[illegible]
61789641	1576	student	2017.0	4.0	4.0	0	0	1	0	0	0	0	0	1	[illegible]
61789727	2639	student	2017.0	4.0	4.0	0	0	0	0	0	0	0	0	1	[illegible]
61790016	812	intern	2016.0	2.0	4.0	0	0	1	0	0	0	0	0	1	[illegible]
61790145	3049	student	2017.0	4.0	5.0	0	0	1	0	0	0	0	0	0	[illegible]
61790148	2314	intern	2016.0	4.0	4.0	1	0	0	1	0	0	0	0	0	[illegible]
61790188	2736	other	2014.0	3.0	5.0	1	0	0	0	0	0	0	0	0	[illegible]
61790778	931	student	2017.0	3.0	4.0	0	0	1	0	0	0	0	0	0	[illegible]
61791389	879	intern	2016.0	4.0	4.0	1	0	0	0	0	0	0	0	0	[illegible]
61791463	1156	student	2019.0	4.0	5.0	1	0	0	0	0	0	0	0	0	[illegible]
61792155	2727	intern	2016.0	1.0	2.0	0	0	0	0	0	0	0	1	1	[illegible]
61792929	750	intern	2012.0	2.0	4.0	0	0	1	0	0	0	0	0	0	[illegible]
61793635	1103	student	2019.0	2.0	4.0	0	0	0	0	1	0	0	0	1	[illegible]
61793901	2677	student	2017.0	3.0	5.0	0	0	0	0	1	0	0	0	1	[illegible]
61793912	4597	other	2015.0	2.0	3.0	1	0	0	0	0	0	0	0	0	[illegible]
61793942	811	student	2017.0	3.0	4.0	0	0	1	0	1	0	0	0	0	[illegible]
61793951	3896	student	2019.0	2.0	4.0	0	0	1	0	0	1	0	0	1	[illegible]
61795878	2122	student	2018.0	3.0	5.0	1	0	0	0	0	0	0	0	1	[illegible]
61795944	858	intern	2016.0	4.0	4.0	1	0	0	0	0	0	0	0	0	[illegible]
61796040	943	intern	2015.0	3.0	4.0	0	0	1	0	0	0	0	0	0	[illegible]
61796080	1211	other	2012.0	5.0	6.0	0	0	1	0	0	1	0	0	0	[illegible]
61796421	1178	student	2018.0	2.0	2.0	0	0	0	0	0	0	0	0	0	[illegible]
61796471	1697	student	2019.0	3.0	3.0	0	0	0	1	0	0	0	0	0	[illegible]
61796700	1315	intern	2015.0	5.0	4.0	1	0	0	0	0	0	0	0	0	[illegible]
61796751	958	pediatrician	2015.0	3.0	3.0	1	0	0	0	0	0	0	0	0	[illegible]
61797051	938	intern	2016.0	4.0	4.0	1	0	0	0	0	0	0	0	0	[illegible]
61797332	1013	pediatrician	2015.0	3.0	3.0	1	0	0	0	0	1	0	0	0	[illegible]
61798111	987	student	2018.0	4.0	5.0	1	0	0	0	0	0	0	0	0	[illegible]
61798276	1888	other	2015.0	2.0	3.0	1	0	0	0	0	0	0	0	0	[illegible]
61798416	1159	intern	2016.0	4.0	5.0	1	0	0	0	0	0	0	0	0	[illegible]
61798515	1196	intern	2014.0	4.0	5.0	0	0	0	0	0	0	0	0	1	[illegible]
61798535	2168	student	2017.0	4.0	5.0	1	0	0	0	0	0	0	0	1	[illegible]
61798869	1121	pediatrician	2015.0	5.0	5.0	0	0	0	0	0	0	1	0	0	[illegible]
61800257	4697	intern	2016.0	3.0	4.0	0	0	1	0	0	0	0	0	0	[illegible]
61801321	610	student	2018.0	3.0	4.0	1	0	0	0	0	0	0	0	0	[illegible]
61802092	1088	student	2017.0	3.0	4.0	0	0	0	0	0	0	0	0	0	[illegible]
61802811	803	other	2015.0	5.0	5.0	0	0	1	0	0	0	1	0	0	[illegible]
61803091	3299	student	2017.0	5.0	5.0	1	0	0	0	0	0	0	0	0	[illegible]
61804171	866	student	2019.0	3.0	4.0	1	0	0	0	0	0	0	0	0	[illegible]
61804673	4123	student	2018.0	3.0	4.0	1	0	0	0	0	0	0	0	0	[illegible]
61804799	997	other	2012.0	2.0	4.0	0	0	1	0	0	0	0	0	0	[illegible]
61804890	743	other	2012.0	4.0	5.0	1	0	0	0	0	0	0	0	0	[illegible]
61806367	3542	intern	2016.0	4.0	4.0	0	0	0	0	0	0	0	1	1	[illegible]
61807301	1378	intern	2014.0	2.0	4.0	1	0	0	0	0	0	0	0	0	[illegible]
61807357	667	student	2019.0	1.0	4.0	1	0	0	0	0	0	0	0	0	[illegible]
61807427	856	intern	2016.0	4.0	5.0	1	0	0	0	0	0	0	0	0	[illegible]
61807457	1382	other	2013.0	3.0	4.0	0	0	0	0	1	0	0	0	0	[illegible]
61807571	563	other	2016.0	3.0	3.0	0	0	1	0	0	0	0	0	1	[illegible]
61807879	743	other	2014.0	4.0	4.0	0	0	1	0	0	0	0	0	1	[illegible]
61809569	1865	intern	2014.0	4.0	5.0	1	0	0	0	0	0	0	0	0	[illegible]
61809911	1200	student	2018.0	3.0	4.0	1	0	0	0	0	0	0	0	0	[illegible]
61809988	1847	intern	2016.0	3.0	4.0	0	0	0	0	0	1	0	0	1	[illegible]
61811734	1530	student	2018.0	1.0	3.0	0	0	1	0	0	0	0	0	0	[illegible]
61813458	2740	student	2017.0	3.0	3.0	1	0	0	0	0	0	0	0	1	[illegible]
61827063	746	pediatrician	2014.0	5.0	4.0	0	0	1	0	0	0	0	0	0	[illegible]
61829267	1225	student	2018.0	4.0	4.0	0	0	1	0	0	0	0	0	0	[illegible]
61830568	1196	other	2015.0	3.0	4.0	0	0	0	0	1	0	0	0	0	[illegible]
61832021	592	other	2012.0	3.0	4.0	1	0	0	0	0	0	0	0	0	[illegible]
61833114	705	other	2014.0	4.0	5.0	1	0	0	0	0	0	0	0	0	[illegible]
61834286	842	student	2017.0	2.0	3.0	0	0	1	0	0	0	0	0	0	[illegible]
61835676	1085	intern	2016.0	3.0	4.0	0	1	0	0	0	0	0	0	0	[illegible]
61835965	717	student	2020.0	1.0	1.0	1	0	0	0	0	0	0	0	0	[illegible]
61840033	382	student	2017.0	2.0	2.0	1	0	0	0	0	0	0	0	0	[illegible]
61842216	813	student	2018.0	2.0	3.0	0	0	0	0	0	0	0	1	0	[illegible]
61844843	1383	intern	2015.0	3.0	4.0	0	0	1	0	0	0	0	0	0	[illegible]
61845285	4988	student	2019.0	5.0	4.0	0	0	0	0	0	0	0	0	0	[illegible]
61846070	4399	student	2019.0	3.0	4.0	1	0	0	0	0	0	0	0	0	[illegible]
61849089	906	student	2020.0	3.0	5.0	1	0	0	0	0	0	0	0	0	[illegible]
61853325	685	intern	2016.0	4.0	4.0	0	0	0	0	0	1	0	0	0	[illegible]
61853579	679	other	2014.0	2.0	5.0	1	0	0	0	0	0	0	0	0	[illegible]
61854305	1164	other	2015.0	2.0	4.0	0	0	0	0	0	0	0	0	0	[illegible]
61854369	5428	intern	2016.0	5.0	5.0	0	0	0	0	0	1	0	0	1	[illegible]
61855024	1222	intern	2016.0	3.0	5.0	1	0	0	0	0	0	0	0	0	[illegible]
61859170	1138	intern	2016.0	3.0	4.0	0	0	0	0	0	0	0	0	1	[illegible]
61861761	4195	other	2014.0	2.0	4.0	1	0	0	0	0	0	0	0	0	[illegible]
61861781	867	other	2013.0	4.0	5.0	0	0	1	0	0	0	0	0	0	[illegible]
61863370	1223	student	2017.0	5.0	5.0	0	0	1	0	0	0	0	0	1	[illegible]
61887179	1145	other	2015.0	4.0	5.0	0	0	1	0	0	0	0	0	0	[illegible]
61899360	1381	intern	2016.0	3.0	5.0	0	0	0	0	1	0	0	1	0	[illegible]
61902153	937	student	2017.0	1.0	4.0	0	0	1	0	0	0	0	0	1	[illegible]
61903011	813	student	2017.0	1.0	4.0	0	0	1	0	0	0	0	0	1	[illegible]
61903937	1169	student	2018.0	2.0	3.0	1	0	0	0	0	0	0	0	1	[illegible]
61904276	483	intern	2015.0	2.0	4.0	0	0	0	0	0	0	0	0	1	[illegible]
61920168	2460	pediatrician	2012.0	4.0	4.0	0	0	0	0	0	1	1	0	0	[illegible]
61921696	873	pediatrician	2014.0	5.0	4.0	0	0	1	0	0	0	0	0	0	[illegible]
61940041	712	intern	2016.0	3.0	3.0	1	0	0	0	0	0	0	0	0	[illegible]
61946133	4081	student	2019.0	2.0	3.0	0	0	0	0	0	1	0	0	0	[illegible]
62002392	1135	student	2019.0	2.0	4.0	0	0	1	0	0	0	0	0	1	[illegible]
62016284	841	student	2022.0	2.0	2.0	0	1	0	0	0	0	0	0	1	[illegible]
62025026	1451	pediatrician	2015.0	3.0	5.0	1	0	0	0	0	0	0	0	0	[illegible]
62059842	918	student	2018.0	3.0	4.0	0	0	0	0	0	1	0	0	0	[illegible]
62089454	938	student	2019.0	3.0	4.0	1	0	0	0	0	0	0	0	0	[illegible]
62115195	1626	pulmonologist	2015.0	4.0	4.0	0	0	0	0	0	0	0	1	0	[illegible]
62135515	1089	intern	2015.0	5.0	6.0	0	0	1	0	0	0	0	0	1	[illegible]
62190082	722	student	2018.0	3.0	5.0	0	0	0	0	0	0	0	0	1	[illegible]
62196224	1066	pediatrician	2018.0	3.0	5.0	0	0	0	0	0	0	1	1	0	[illegible]
62324658	653	intern	2016.0	2.0	2.0	0	0	1	0	0	0	0	0	0	[illegible]
62334491	668	student	2018.0	3.0	4.0	0	0	1	0	0	0	0	0	0	[illegible]
62334541	1028	student	2018.0	3.0	5.0	0	0	1	0	1	0	0	0	0	[illegible]
62345294	912	student	2018.0	3.0	4.0	0	0	1	0	0	1	0	0	1	[illegible]
62355092	960	other	2014.0	3.0	4.0	0	0	1	0	0	0	0	0	0	[illegible]
62378446	2914	intern	2015.0	2.0	2.0	0	0	1	0	0	0	0	0	1	[illegible]
62411447	1019	student	2018.0	3.0	5.0	1	0	0	0	0	0	0	0	0	[illegible]
62417067	2129	pediatrician	2010.0	5.0	4.0	0	0	1	0	0	0	0	0	1	[illegible]
62521947	3431	student	2017.0	3.0	4.0	1	0	0	0	0	0	0	0	0	[illegible]
62525047	2500	intern	2015.0	3.0	4.0	1	0	0	0	0	0	0	0	0	[illegible]
62773201	1630	pediatrician	2012.0	5.0	3.0	0	0	0	0	1	0	0	0	0	[illegible]
62835025	1076	intern	2016.0	3.0	4.0	1	0	0	0	0	0	0	0	0	[illegible]
62839736	1680	intern	2016.0	4.0	5.0	0	0	1	0	0	0	0	0	0	[illegible]
62881269	2805	intern	2016.0	1.0	3.0	1	0	0	0	0	0	1	0	1	[illegible]
62902488	0	other	2013.0	5.0	5.0	0	0	0	0	0	0	0	0	0	[illegible]
62928084	0	student	2021.0	1.0	1.0	0	0	0	0	0	0	0	0	0	[illegible]
62928816	0	pediatrician	2012.0	6.0	6.0	0	0	0	0	0	0	0	0	0	[illegible]
62938758	1832	intern	2016.0	3.0	4.0	1	0	0	0	0	1	0	0	0	[illegible]
62957684	0	student	2018.0	3.0	3.0	0	0	0	0	0	0	0	0	0	[illegible]
63064242	0	intern				0	0	0	0	0	0	0	0	0	[illegible]
63153368	858	other	2014.0	3.0	4.0	0	0	0	0	0	1	0	0	0	[illegible]
63168889	928	student	2020.0	2.0	4.0	1	0	0	0	0	0	0	0	0	[illegible]
64058180	0	student	2018.0	5.0	5.0	0	0	0	0	0	0	0	0	0	[illegible]
64121396	0	other	2012.0	2.0	5.0	0	0	0	0	0	0	0	0	0	[illegible]
