# Supplementary material for: The accuracy of lung auscultation in the practice of physicians and medical students
Source: PLoS One. 2019 Aug 12;14(8):e0220606. doi: 10.1371/journal.pone.0220606 (PMC6690530; doi:10.1371/journal.pone.0220606)
Supplement: S1 File — (PDF) [file pone.0220606.s002.pdf]

Survey: Dźwięki układu oddechowego

Proszę wpisać adres e-mail. Na wpisany adres zostaną przesłane wyniki ankiety i Państwa odpowiedzi. Pole nie jest obowiązkowe.

Adres e-mail : ______________________

* **Proszę wybrać rozpoczętą lub posiadaną specjalizację.**

- ☐ specjalista pediatrii
- ☐ w trakcie specjalizacji z pediatrii
- ☐ specjalista neonatologii
- ☐ w trakcie specjalizacji z neonatologii
- ☐ specjalista pulmonologii
- ☐ w trakcie specjalizacji z pulmonologii
- ☐ specjalista chorób wewnętrznych
- ☐ w trakcie specjalizacji z chorób wewnętrznych
- ☐ specjalista medycyny rodzinnej
- ☐ w trakcie specjalizacji z medycyny rodzinnej
- ☐ specjalista kardiochirurgii
- ☐ w trakcie specjalizacji z kardiochirurgii
- ☐ specjalista anestezjologii i intensywnej terapii
- ☐ w trakcie specjalizacji z anestezjologii i intensywnej terapii
- ☐ student medycyny (proszę wpisać w polu "inna" rok studiów)
- ☐ inna (proszę wpisać)

____

* **Rok ukończenia studiów medycznych (w przypadku studentów planowany rok ukończenia studiów)**

____

* **Ukończona uczelnia (w przypadku studentów uczelnia na której aktualnie studiuje)**

- ☐ Collegium Medicum Uniwersytetu Jagiellońskiego
- ☐ Warszawski Uniwersytet Medyczny
- ☐ Gdański Uniwersytet Medyczny
- ☐ Uniwersytet Medyczny im. Karola Marcinkowskiego w Poznaniu
- ☐ Uniwersytet Medyczny im. Piastów Śląskich we Wrocławiu

☐ Uniwersytet Medyczny w Łodzi

☐ Uniwersytet Medyczny w Białymstoku

☐ Uniwersytet Medyczny w Lublinie

☐ Pomorski Uniwersytet Medyczny w Szczecinie

☐ Śląski Uniwersytet Medyczny w Katowicach

☐ Collegium Medicum UMK im. Ludwika Rydygiera w Bydgoszczy

☐ inna (proszę wpisać)

W skali od 0-5 (gdzie 0 oznacza bardzo słabo a 5 bardzo dobrze) jak ocenia Pan/Pani swoje umiętności związane z osłuchiwaniem układu oddechowego

	0	1	2	3	4	5
* dziecka	○	○	○	○	○	○
* dorosłego człowieka	○	○	○	○	○	○

Proszę o zaznaczenie w jakim stopniu zgadza się Pan/Pani z poniższymi stwierdzeniami

	zdecydowanie nie zgadzam się	nie zgadzam się	częściowo zgadzam się	zgadzam się	zdecydowanie zgadzam się
* Liczba godzin w trakcie studiów poświęcona na naukę osłuchiwania układu oddechowego jest dla mnie NIEWYSTARCZAJĄCA	○	○	○	○	○
* Liczba godzin przeznaczona na szkolenia w zakresie osłuchiwania układu oddechowego podczas stażu i specjalizacji jest dla mnie NIEWYSTARCZAJĄCA	○	○	○	○	○
* Dodatkowe szkolenia w zakresie osłuchiwania układu oddechowego są dla lekarzy mojej specjalności potrzebne	○	○	○	○	○
* Nazewnictwo związane z dźwiękami osłuchowymi dla układu oddechowego jest niespójne i potrzebuje uporządkowania	○	○	○	○	○

Jak często w swojej praktyce (lub zajęć klinicznych) osłuchuje Pan/Pani za pomocą stetoskopu

	codziennie	klika razy w tygodniu	przynajmniej raz w tygodniu	przynajmniej raz w miesiącu	rzadziej niż raz w miesiącu	nie osłuchuję pacjentów
* dzieci	○	○	○	○	○	○
* osoby dorosłe	○	○	○	○	○	○

*
Czy posiada Pan/Pani wykształcenie muzyczne?

☐ nie

☐ szkoła podstawowa muzyczna

☐ średnia szkoła muzyczna

☐ śpiew w chórze

☐ inne (wpisz jakie)

*** Czy w swojej praktyce używa Pan/Pani elektronicznego stetoskopu?**

○ tak

○ nie

*** Podczas osłuchiwania płuc używa Pan/Pani**

☐ membrany stetoskopu

☐ lejka stetoskopu

☐ inne (wpisz jakie)

Patrząc na rysunek umieszczony poniżej zaznacz numery punktów w których standardowo osłuchujesz układ oddechowy

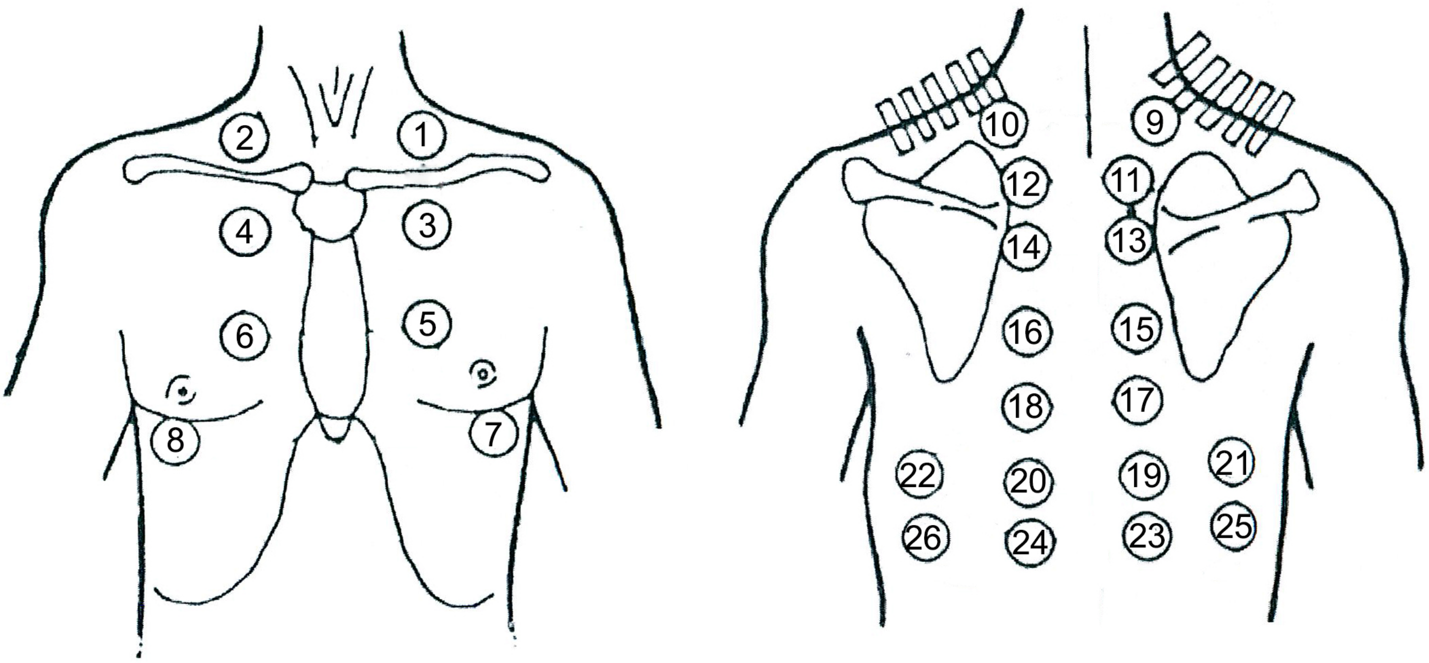

	1	2	3	4	5	6	7	8	9	10	11	12	13	14	15	16	17	18	19	20	21	22	23	24	25	26
* dziecka	☐	☐	☐	☐	☐	☐	☐	☐	☐	☐	☐	☐	☐	☐	☐	☐	☐	☐	☐	☐	☐	☐	☐	☐	☐	☐
* dorosłego pacjenta	☐	☐	☐	☐	☐	☐	☐	☐	☐	☐	☐	☐	☐	☐	☐	☐	☐	☐	☐	☐	☐	☐	☐	☐	☐	☐

Badania osłuchowe układu oddechowego - część praktyczna

W tej części przedstawione zostaną Panu/Pani nagrania osłuchowe dla kolejnych 24 pacjentów.
Bardzo ważne jest używanie w trakcie odsłuchu słuchawek (najlepiej wysokiej jakości)

Na ilustracji zaznaczony jest punkt, w którym został przyłożony stetoskop i zarejestrowany dźwięk. Poniżej znajduje się nagranie, które należy odsłuchać. Podczas odsłuchiwania można w miarę potrzeb regulować głośność dźwięku. Dźwięk można odtwarzać wielokrotnie.
Proszę o sklasyfikowanie każdego dźwięku poprzez wybór z listy (może to być wybór wielu cech jednocześnie).

---

*

Pacjent 1

Wiek: 11 lat

Wzrost: 147 cm

Waga: 32 kg

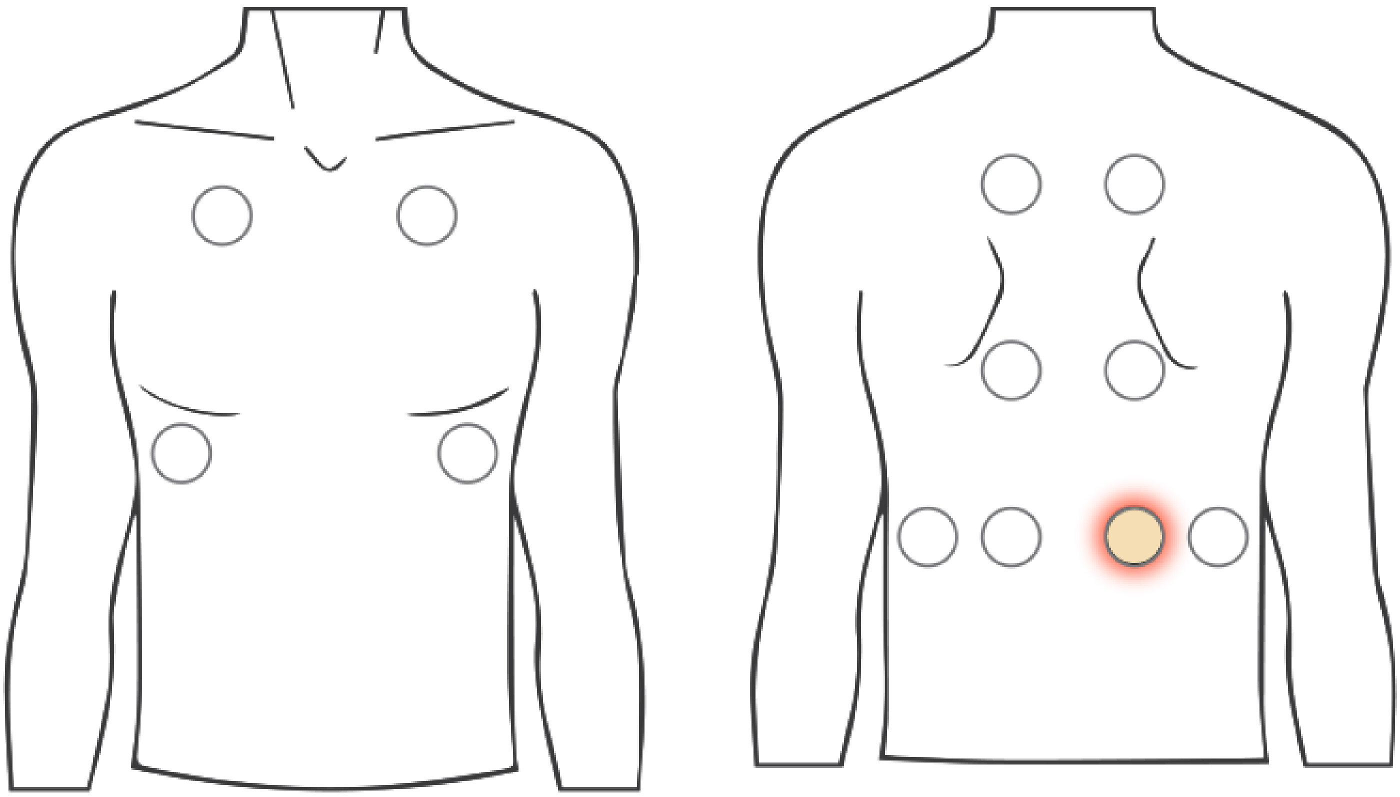

- ☐ Szmer pęcherzykowy prawidłowy
- ☐ Szmer pęcherzykowy ściszony
- ☐ Szmer pęcherzykowy zaostrzony
- ☐ Szmer oskrzelowy prawidłowy
- ☐ Szmer oskrzelowy patologiczny (w nieprawidłowym miejscu)
- ☐ Rzężenia drobnobańkowe
- ☐ Rzężenia średniobańkowe

QuestionPro

- ☐ Rzężenia grubobańkowe
- ☐ Trzeszczenia
- ☐ Świsty wdechowe
- ☐ Świsty wydechowe
- ☐ Stridor
- ☐ Wydłużenie fazy wydechowej
- ☐ Skrzeczenia
- ☐ Tarcie opłucnowe
- ☐ Furczenia
- ☐ Uwagi

---

*

Pacjent 2

Wiek: 3 lat
Wzrost: 96 cm
Waga: 12 kg

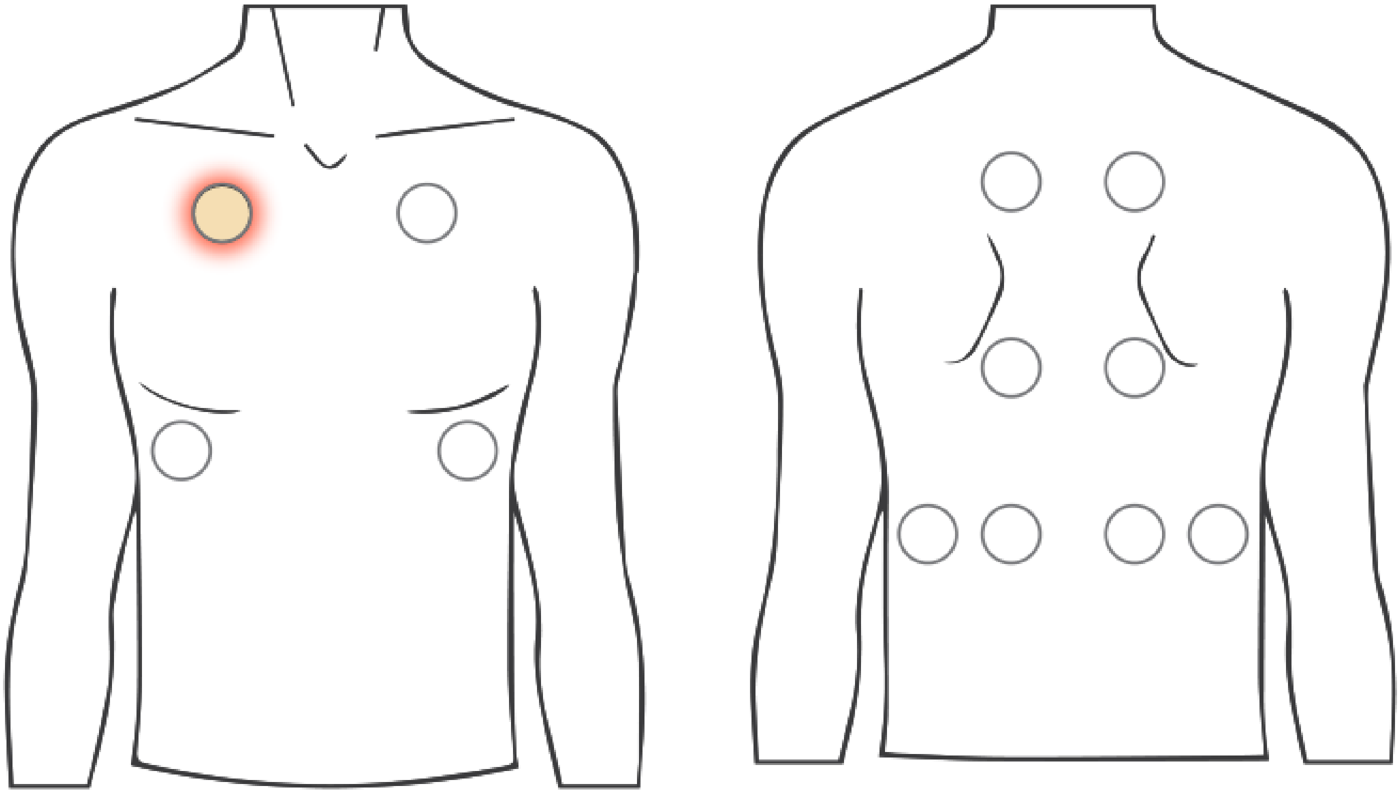

- ☐ Szmer pęcherzykowy prawidłowy
- ☐ Szmer pęcherzykowy ściszony

☐ Szmer pęcherzykowy zaostrzony

☐ Szmer oskrzelowy prawidłowy

☐ Szmer oskrzelowy patologiczny (w nieprawidłowym miejscu)

☐ Rzężenia drobnobańkowe

☐ Rzężenia średniobańkowe

☐ Rzężenia grubobańkowe

☐ Trzeszczenia

☐ Świsty wdechowe

☐ Świsty wydechowe

☐ Stridor

☐ Wydłużenie fazy wydechowej

☐ Skrzeczenia

☐ Tarcie opłucnowe

☐ Furczenia

☐ Uwagi

---

*

Pacjent 3

Wiek: 6 lat

Wzrost: 118 cm

Waga: 30 kg

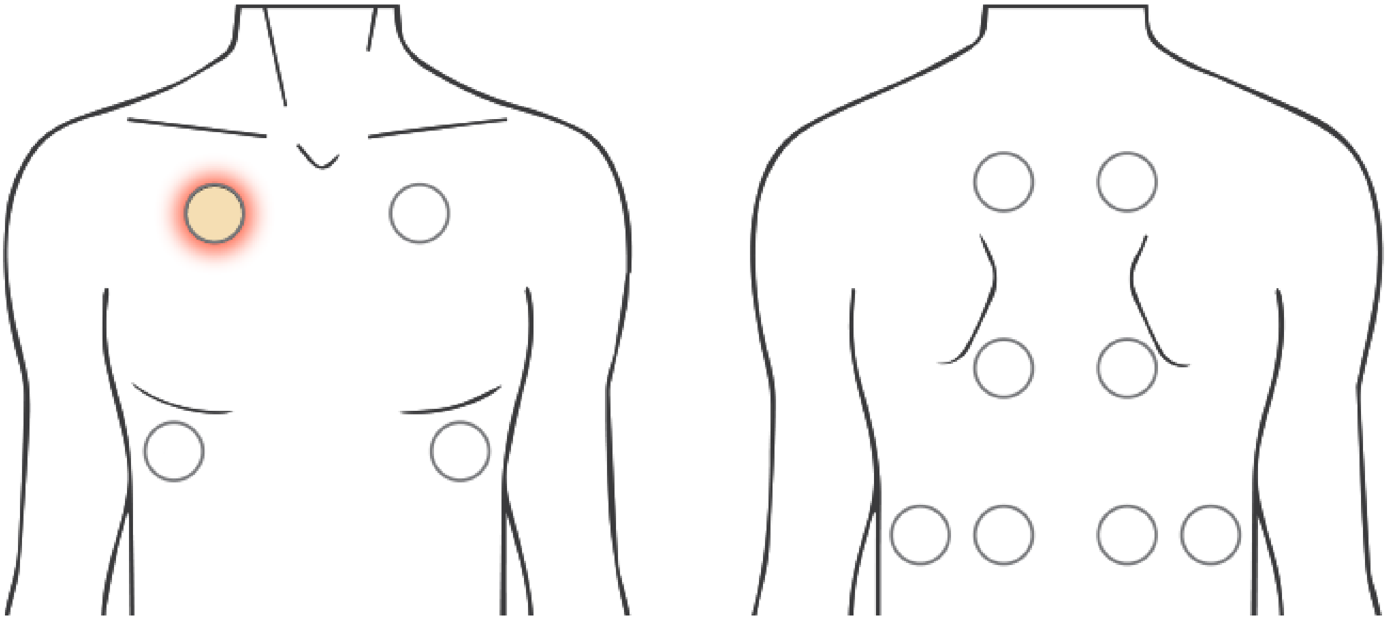

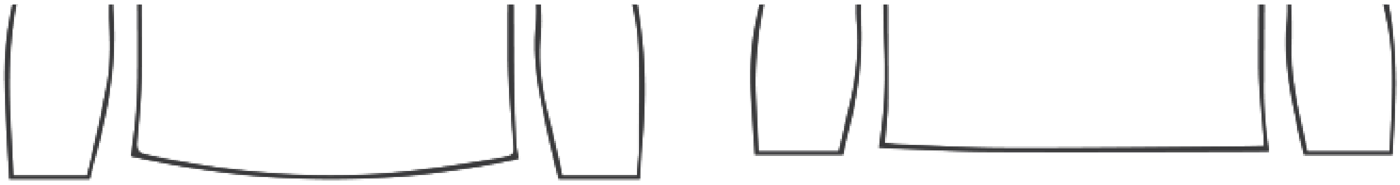

- ☐ Szmer pęcherzykowy prawidłowy
- ☐ Szmer pęcherzykowy ściszony
- ☐ Szmer pęcherzykowy zaostrzony
- ☐ Szmer oskrzelowy prawidłowy
- ☐ Szmer oskrzelowy patologiczny (w nieprawidłowym miejscu)
- ☐ Rzężenia drobnobańkowe
- ☐ Rzężenia średniobańkowe
- ☐ Rzężenia grubobańkowe
- ☐ Trzeszczenia
- ☐ Świsty wdechowe
- ☐ Świsty wydechowe
- ☐ Stridor
- ☐ Wydłużenie fazy wydechowej
- ☐ Skrzeczenia
- ☐ Tarcie opłucnowe
- ☐ Furczenia
- ☐ Uwagi

*

Pacjent 4

Wiek: 7 lat
Wzrost: 115 cm
Waga: 21 kg

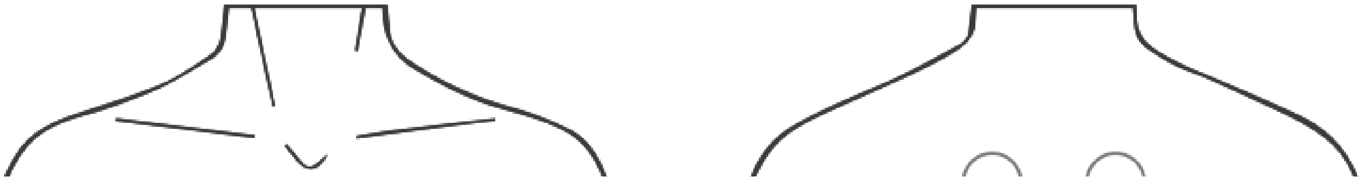

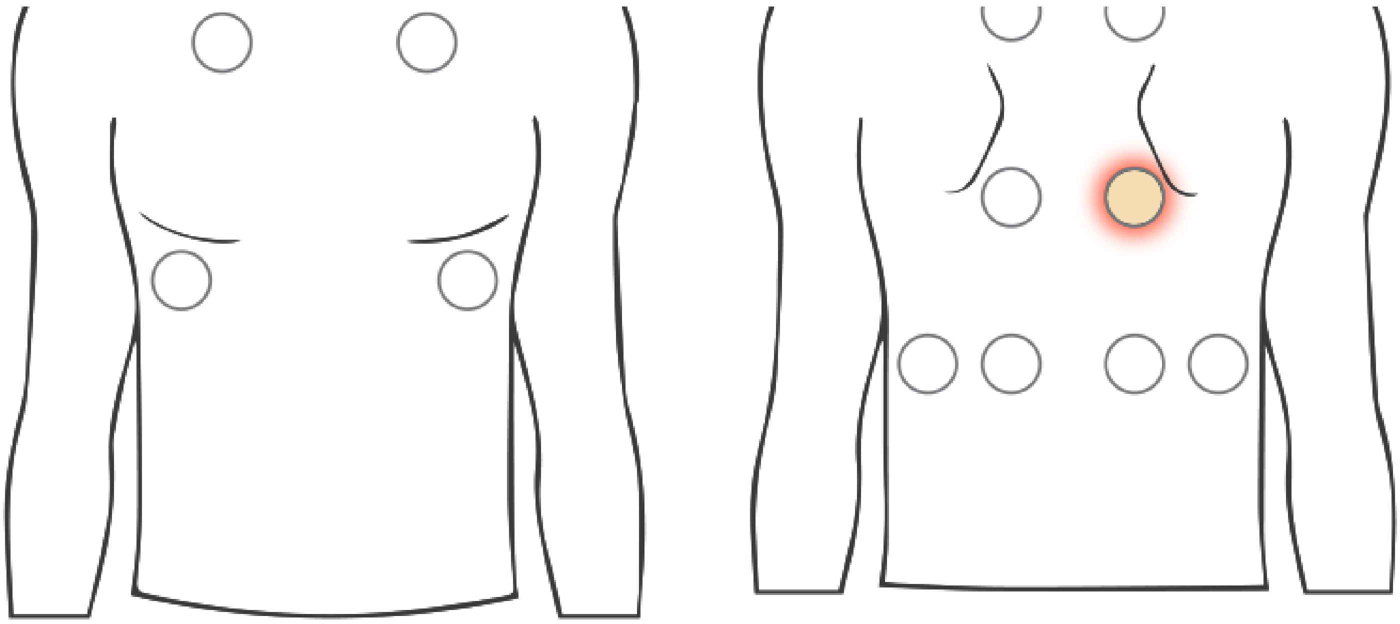

- ☐ Szmer pęcherzykowy prawidłowy
- ☐ Szmer pęcherzykowy ściszony
- ☐ Szmer pęcherzykowy zaostrzony
- ☐ Szmer oskrzelowy prawidłowy
- ☐ Szmer oskrzelowy patologiczny (w nieprawidłowym miejscu)
- ☐ Rzężenia drobnobańkowe
- ☐ Rzężenia średniobańkowe
- ☐ Rzężenia grubobańkowe
- ☐ Trzeszczenia
- ☐ Świsty wdechowe
- ☐ Świsty wydechowe
- ☐ Stridor
- ☐ Wydłużenie fazy wydechowej
- ☐ Skrzeczenia
- ☐ Tarcie opłucnowe
- ☐ Furczenia
- ☐ Uwagi

---

*

Pacjent 5

Wiek: 18 lat

Wzrost: 168 cm
Waga: 50 kg

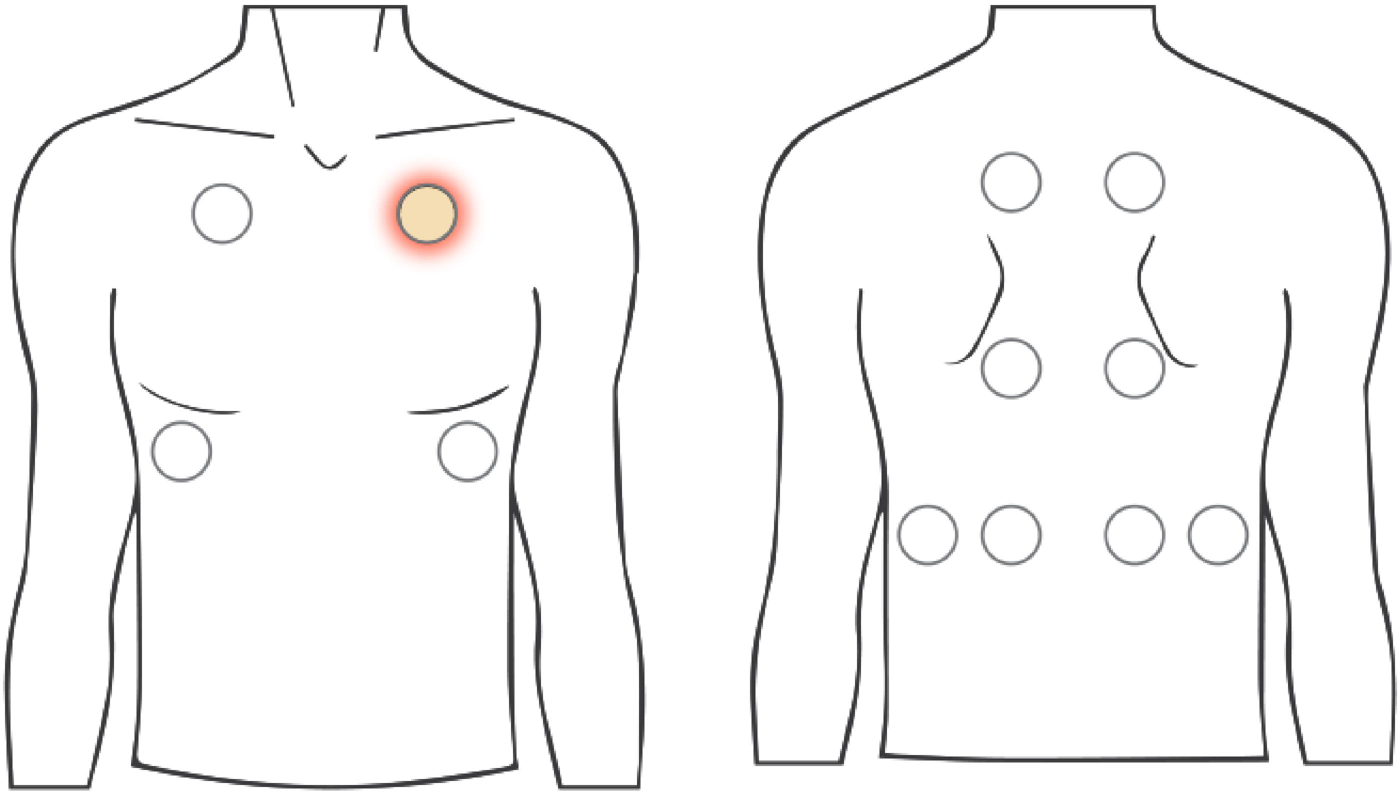

- ☐ Szmer pęcherzykowy prawidłowy
- ☐ Szmer pęcherzykowy ściszony
- ☐ Szmer pęcherzykowy zaostrzony
- ☐ Szmer oskrzelowy prawidłowy
- ☐ Szmer oskrzelowy patologiczny (w nieprawidłowym miejscu)
- ☐ Rzężenia drobnobańkowe
- ☐ Rzężenia średniobańkowe
- ☐ Rzężenia grubobańkowe
- ☐ Trzeszczenia
- ☐ Świsty wdechowe
- ☐ Świsty wydechowe
- ☐ Stridor
- ☐ Wydłużenie fazy wydechowej
- ☐ Skrzeczenia
- ☐ Tarcie opłucnowe
- ☐ Furczenia
- ☐ Uwagi

QuestionPro

*

Pacjent 6

Wiek: 5 miesięcy
Wzrost: 78 cm
Waga: 7 kg

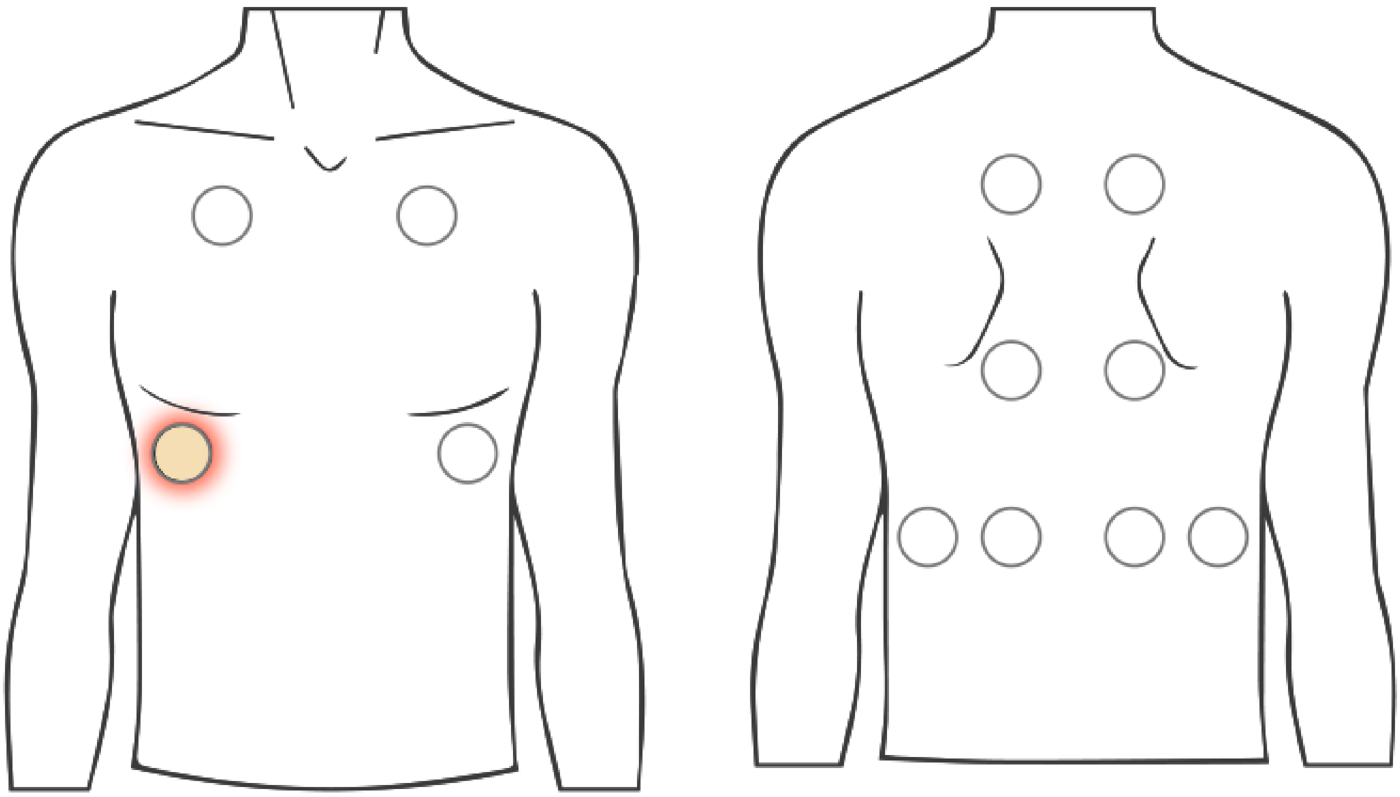

- ☐ Szmer pęcherzykowy prawidłowy
- ☐ Szmer pęcherzykowy ściszony
- ☐ Szmer pęcherzykowy zaostrzony
- ☐ Szmer oskrzelowy prawidłowy
- ☐ Szmer oskrzelowy patologiczny (w nieprawidłowym miejscu)
- ☐ Rzężenia drobnobańkowe
- ☐ Rzężenia średniobańkowe
- ☐ Rzężenia grubobańkowe
- ☐ Trzeszczenia
- ☐ Świsty wdechowe
- ☐ Świsty wydechowe
- ☐ Stridor

QuestionPro

☐ Wydłużenie fazy wydechowej

☐ Skrzeczenia

☐ Tarcie opłucnowe

☐ Furczenia

☐ Uwagi

*

Pacjent 7

Wiek: 2 lata
Wzrost: 81 cm
Waga: 10 kg

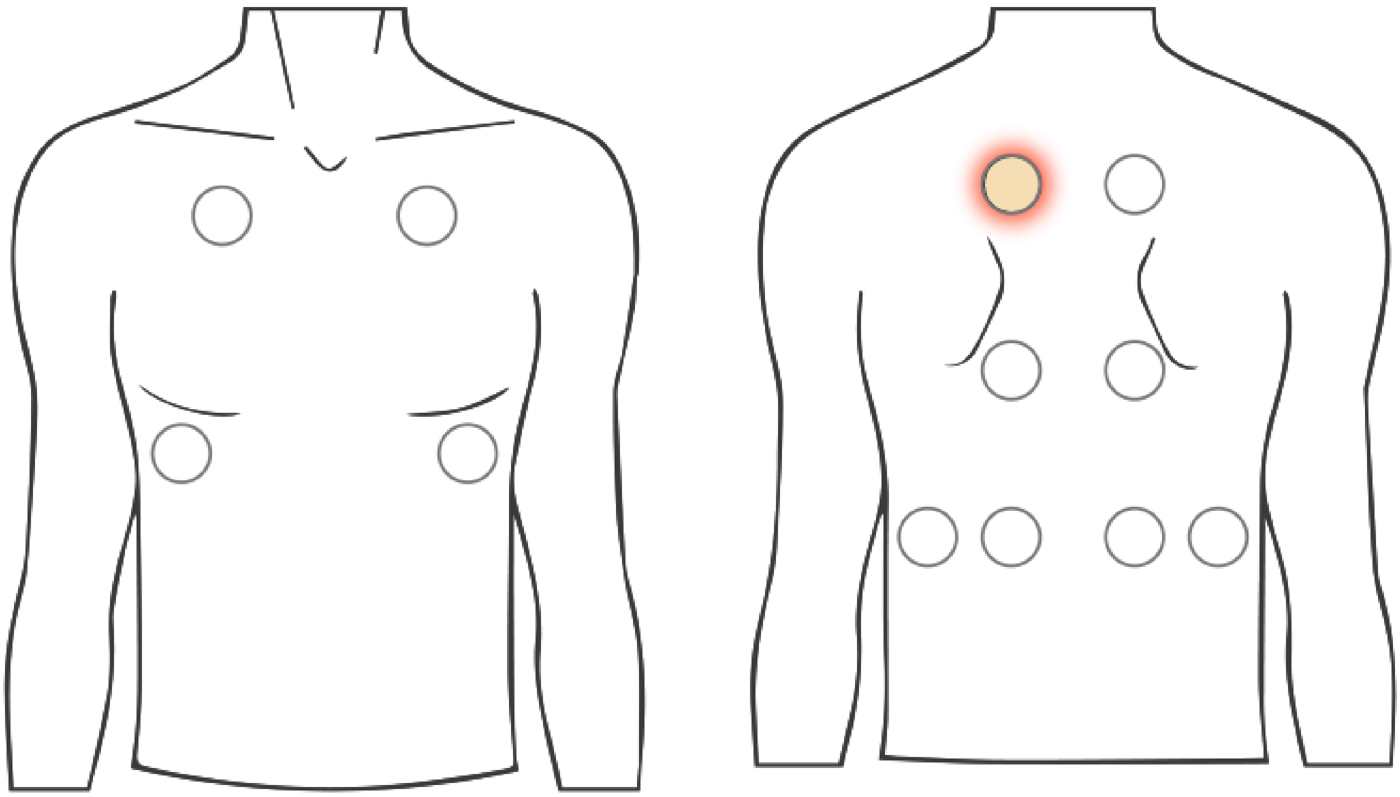

☐ Szmer pęcherzykowy prawidłowy

☐ Szmer pęcherzykowy ściszony

☐ Szmer pęcherzykowy zaostrzony

☐ Szmer oskrzelowy prawidłowy

☐ Szmer oskrzelowy patologiczny (w nieprawidłowym miejscu)

☐ Rzężenia drobnobańkowe

☐ Rzężenia średniobańkowe

☐ Rzężenia grubobańkowe

QuestionPro

☐ Trzeszczenia

☐ Świsty wdechowe

☐ Świsty wydechowe

☐ Stridor

☐ Wydłużenie fazy wydechowej

☐ Skrzeczenia

☐ Tarcie opłucnowe

☐ Furczenia

☐ Uwagi

---

*

Pacjent 8

Wiek: 18 lat

Wzrost: 179 cm

Waga: 60 kg

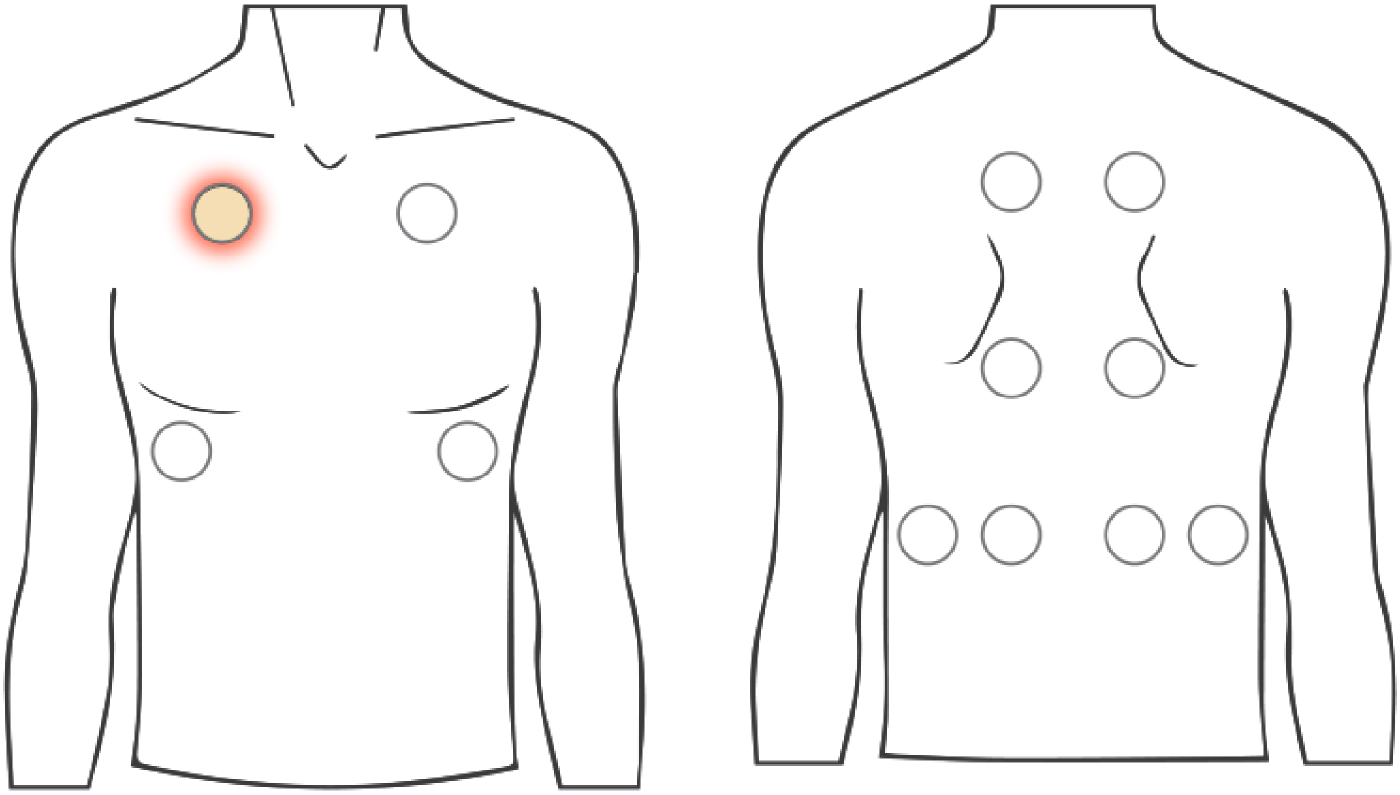

QuestionPro

☐ Szmer pęcherzykowy prawidłowy

☐ Szmer pęcherzykowy ściszony

☐ Szmer pęcherzykowy zaostrzony

☐ Szmer oskrzelowy prawidłowy

☐ Szmer oskrzelowy patologiczny (w nieprawidłowym miejscu)

☐ Rzężenia drobnobańkowe

☐ Rzężenia średniobańkowe

☐ Rzężenia grubobańkowe

☐ Trzeszczenia

☐ Świsty wdechowe

☐ Świsty wydechowe

☐ Stridor

☐ Wydłużenie fazy wydechowej

☐ Skrzeczenia

☐ Tarcie opłucnowe

☐ Furczenia

☐ Uwagi

---

*

Pacjent 9

Wiek: 15 lat

Wzrost: 164 cm

Waga: 42 kg

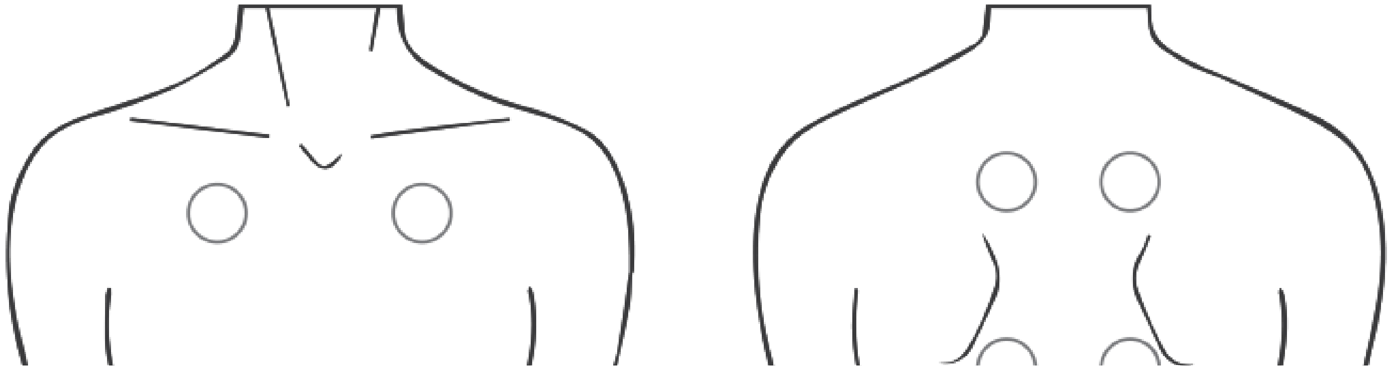

QuestionPro

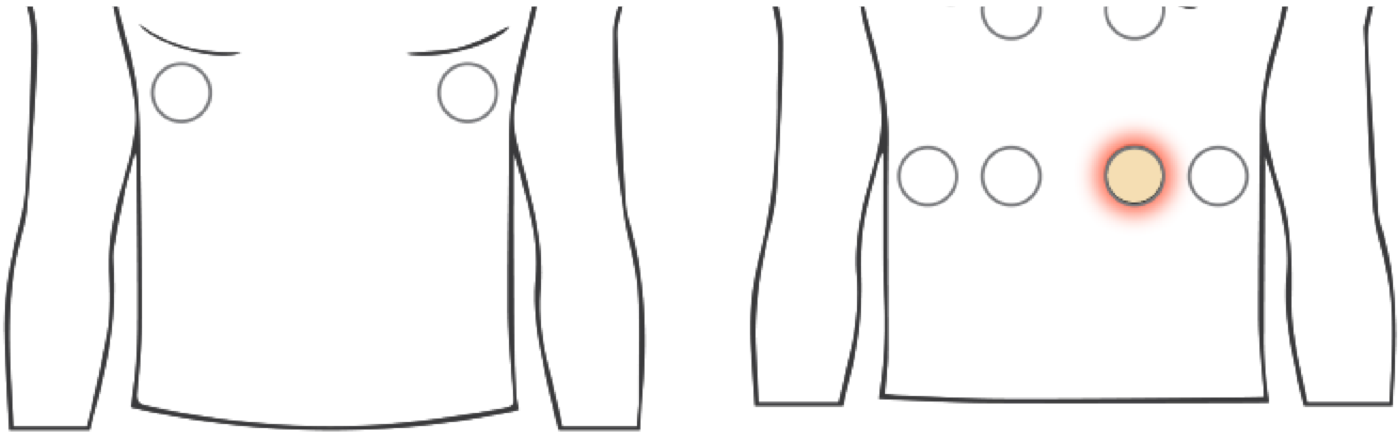

☐ Szmer pęcherzykowy prawidłowy

☐ Szmer pęcherzykowy ściszony

☐ Szmer pęcherzykowy zaostrzony

☐ Szmer oskrzelowy prawidłowy

☐ Szmer oskrzelowy patologiczny (w nieprawidłowym miejscu)

☐ Rzężenia drobnobańkowe

☐ Rzężenia średniobańkowe

☐ Rzężenia grubobańkowe

☐ Trzeszczenia

☐ Świsty wdechowe

☐ Świsty wydechowe

☐ Stridor

☐ Wydłużenie fazy wydechowej

☐ Skrzeczenia

☐ Tarcie opłucnowe

☐ Furczenia

☐ Uwagi

---

*

Pacjent 10

Wiek: 3 lata

Wzrost: 92 cm

Waga: 14 kg

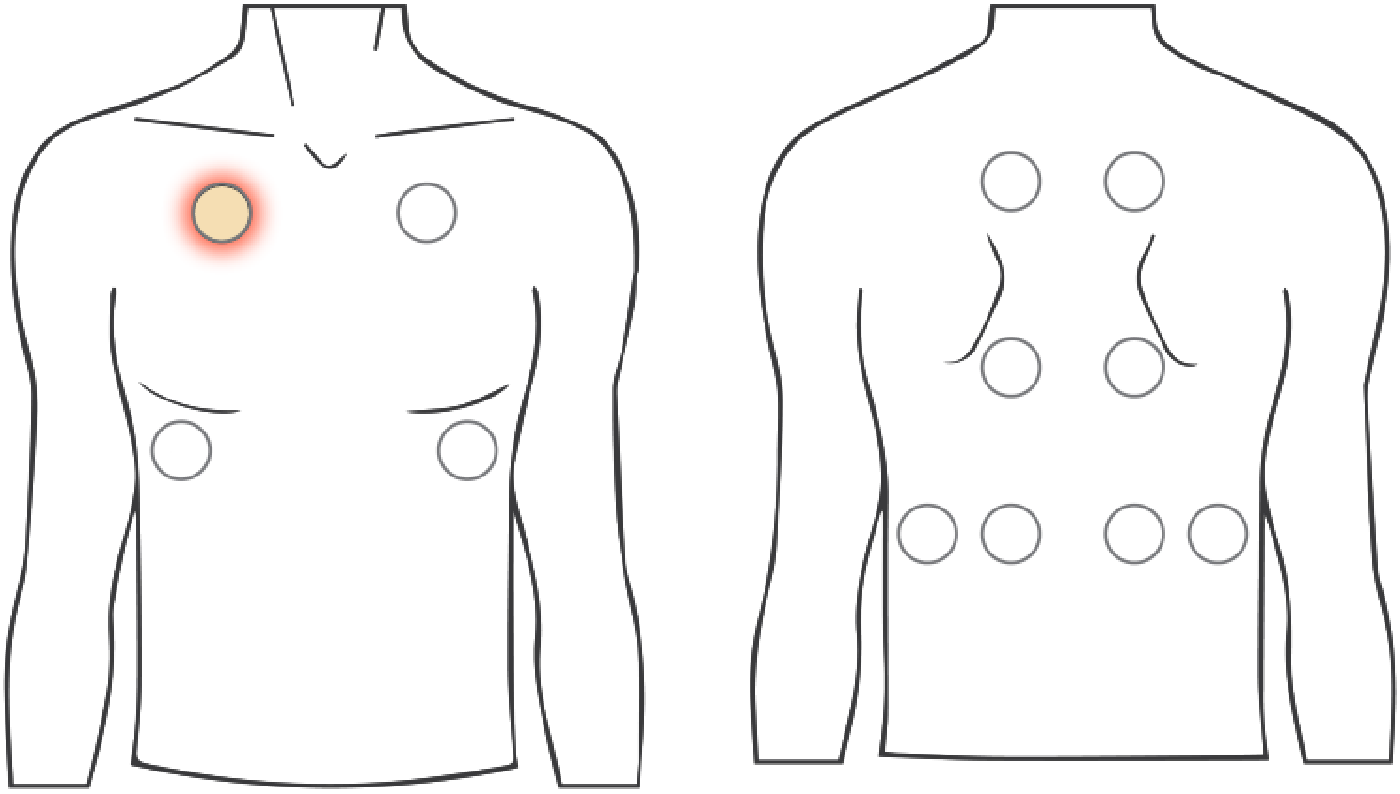

- ☐ Szmer pęcherzykowy prawidłowy
- ☐ Szmer pęcherzykowy ściszony
- ☐ Szmer pęcherzykowy zaostrzony
- ☐ Szmer oskrzelowy prawidłowy
- ☐ Szmer oskrzelowy patologiczny (w nieprawidłowym miejscu)
- ☐ Rzężenia drobnobańkowe
- ☐ Rzężenia średniobańkowe
- ☐ Rzężenia grubobańkowe
- ☐ Trzeszczenia
- ☐ Świsty wdechowe
- ☐ Świsty wydechowe
- ☐ Stridor

☐ Wydłużenie fazy wydechowej

☐ Skrzeczenia

☐ Tarcie opłucnowe

☐ Furczenia

☐ Uwagi

---

*

Pacjent 11

Wiek: 7 miesięc

Wzrost: 82 cm

Waga: 8 kg

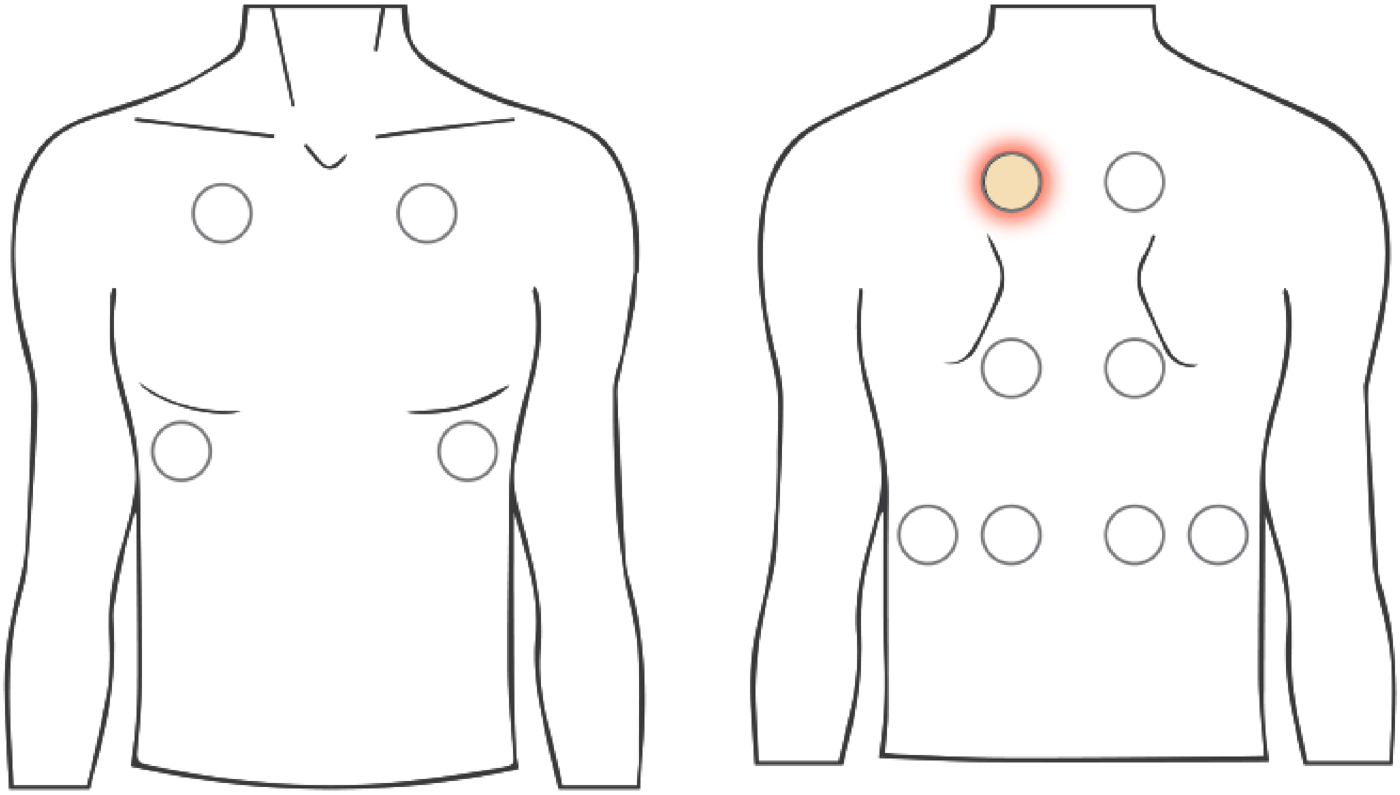

☐ Szmer pęcherzykowy prawidłowy

☐ Szmer pęcherzykowy ściszony

☐ Szmer pęcherzykowy zaostrzony

QuestionPro

☐ Szmer oskrzelowy prawidłowy

☐ Szmer oskrzelowy patologiczny (w nieprawidłowym miejscu)

☐ Rzężenia drobnobańkowe

☐ Rzężenia średniobańkowe

☐ Rzężenia grubobańkowe

☐ Trzeszczenia

☐ Świsty wdechowe

☐ Świsty wydechowe

☐ Stridor

☐ Wydłużenie fazy wydechowej

☐ Skrzeczenia

☐ Tarcie opłucnowe

☐ Furczenia

☐ Uwagi

---

*

Pacjent 12

Wiek: 17 lat

Wzrost: 150 cm

Waga: 50 kg

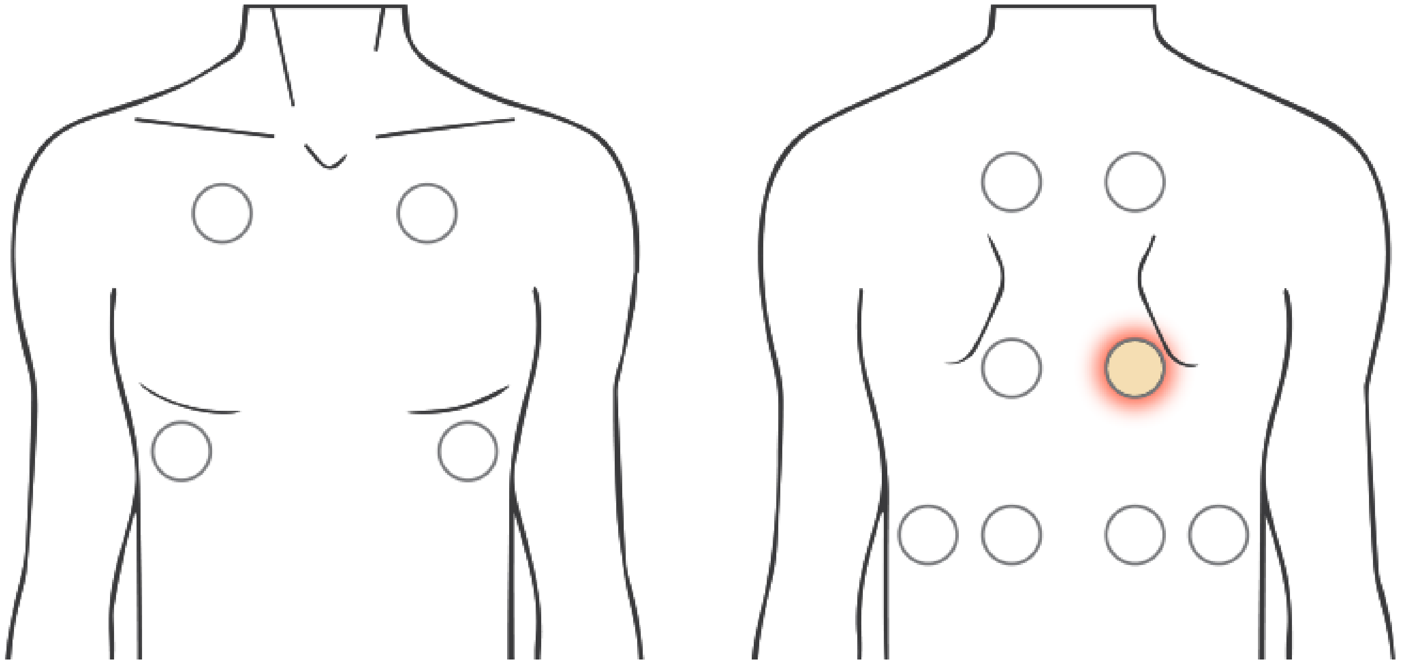

QuestionPro

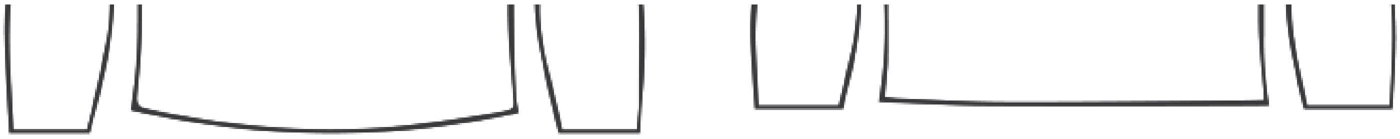

- ☐ Szmer pęcherzykowy prawidłowy
- ☐ Szmer pęcherzykowy ściszony
- ☐ Szmer pęcherzykowy zaostrzony
- ☐ Szmer oskrzelowy prawidłowy
- ☐ Szmer oskrzelowy patologiczny (w nieprawidłowym miejscu)
- ☐ Rzężenia drobnobańkowe
- ☐ Rzężenia średniobańkowe
- ☐ Rzężenia grubobańkowe
- ☐ Trzeszczenia
- ☐ Świsty wdechowe
- ☐ Świsty wydechowe
- ☐ Stridor
- ☐ Wydłużenie fazy wydechowej
- ☐ Skrzeczenia
- ☐ Tarcie opłucnowe
- ☐ Furczenia
- ☐ Uwagi

---

*

Pacjent 13

Wiek: 13 lat

Wzrost: 148 cm

Waga: 33 kg

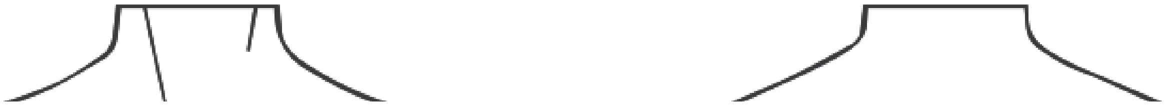

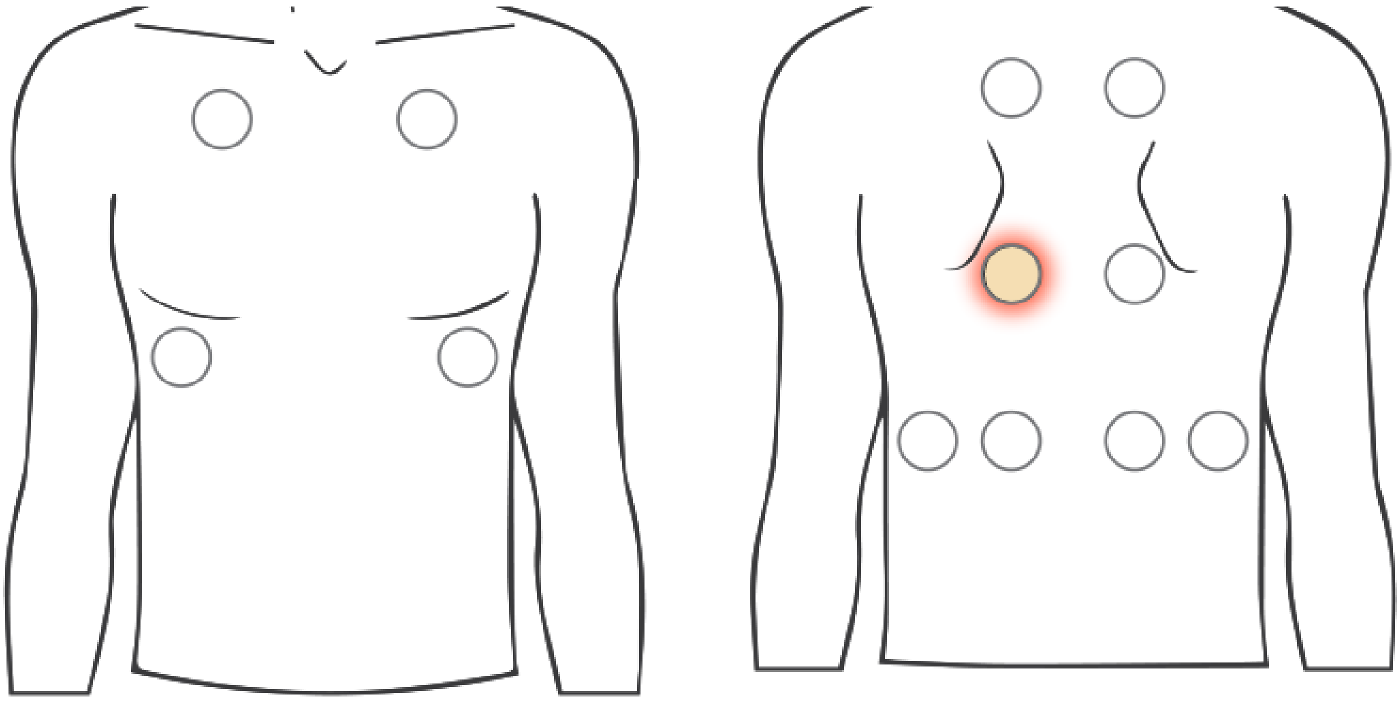

- ☐ Szmer pęcherzykowy prawidłowy
- ☐ Szmer pęcherzykowy ściszony
- ☐ Szmer pęcherzykowy zaostrzony
- ☐ Szmer oskrzelowy prawidłowy
- ☐ Szmer oskrzelowy patologiczny (w nieprawidłowym miejscu)
- ☐ Rzężenia drobnobańkowe
- ☐ Rzężenia średniobańkowe
- ☐ Rzężenia grubobańkowe
- ☐ Trzeszczenia
- ☐ Świsty wdechowe
- ☐ Świsty wydechowe
- ☐ Stridor
- ☐ Wydłużenie fazy wydechowej
- ☐ Skrzeczenia
- ☐ Tarcie opłucnowe
- ☐ Furczenia
- ☐ Uwagi

QuestionPro

*

Pacjent 14

Wiek: 14 lat

Wzrost: 150 cm

Waga: 35 kg

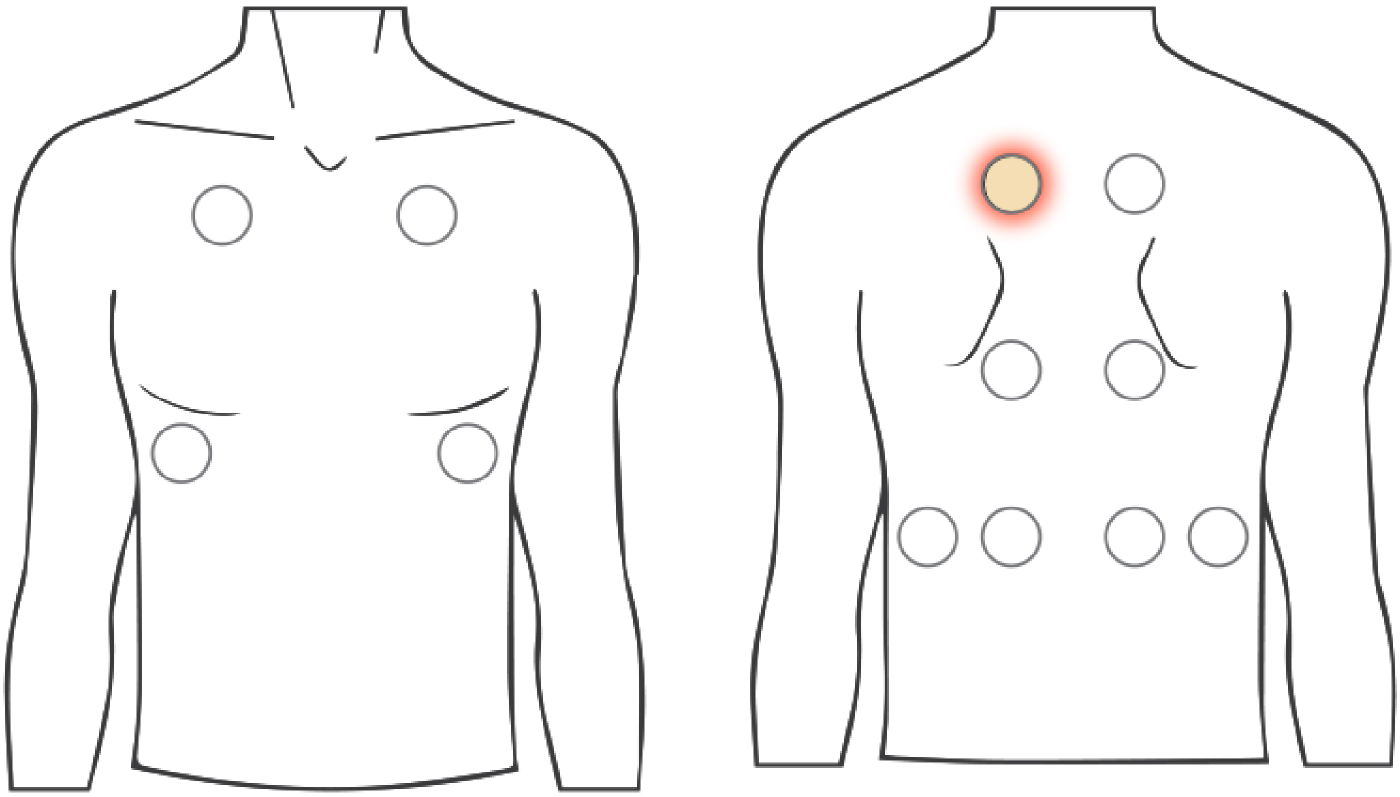

- ☐ Szmer pęcherzykowy prawidłowy
- ☐ Szmer pęcherzykowy ściszony
- ☐ Szmer pęcherzykowy zaostrzony
- ☐ Szmer oskrzelowy prawidłowy
- ☐ Szmer oskrzelowy patologiczny (w nieprawidłowym miejscu)
- ☐ Rzężenia drobnobańkowe
- ☐ Rzężenia średniobańkowe
- ☐ Rzężenia grubobańkowe

QuestionPro

☐ Trzeszczenia

☐ Świsty wdechowe

☐ Świsty wydechowe

☐ Stridor

☐ Wydłużenie fazy wydechowej

☐ Skrzeczenia

☐ Tarcie opłucnowe

☐ Furczenia

☐ Uwagi

*

Pacjent 15

Wiek: 25 lat

Wzrost: 176 cm

Waga: 58 kg

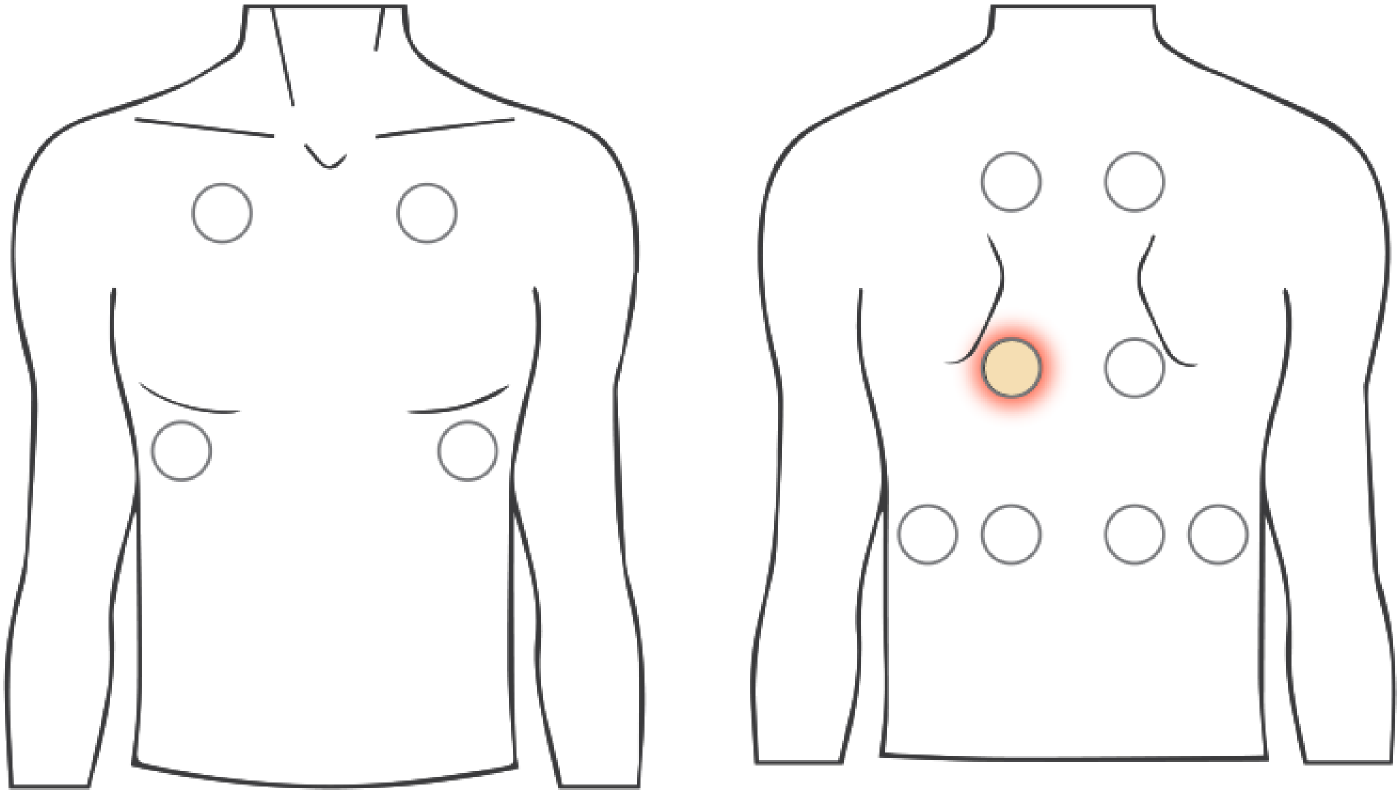

☐ Szmer pęcherzykowy prawidłowy

☐ Szmer pęcherzykowy ściszony

☐ Szmer pęcherzykowy zaostrzony

☐ Szmer oskrzelowy prawidłowy

☐ Szmer oskrzelowy patologiczny (w nieprawidłowym miejscu)

☐ Rzężenia drobnobańkowe

☐ Rzężenia średniobańkowe

☐ Rzężenia grubobańkowe

☐ Trzeszczenia

☐ Świsty wdechowe

☐ Świsty wydechowe

☐ Stridor

☐ Wydłużenie fazy wydechowej

☐ Skrzeczenia

☐ Tarcie opłucnowe

☐ Furczenia

☐ Uwagi

---

*

Pacjent 16

Wiek: 3 lat

Wzrost: 94 cm

Waga: 14 kg

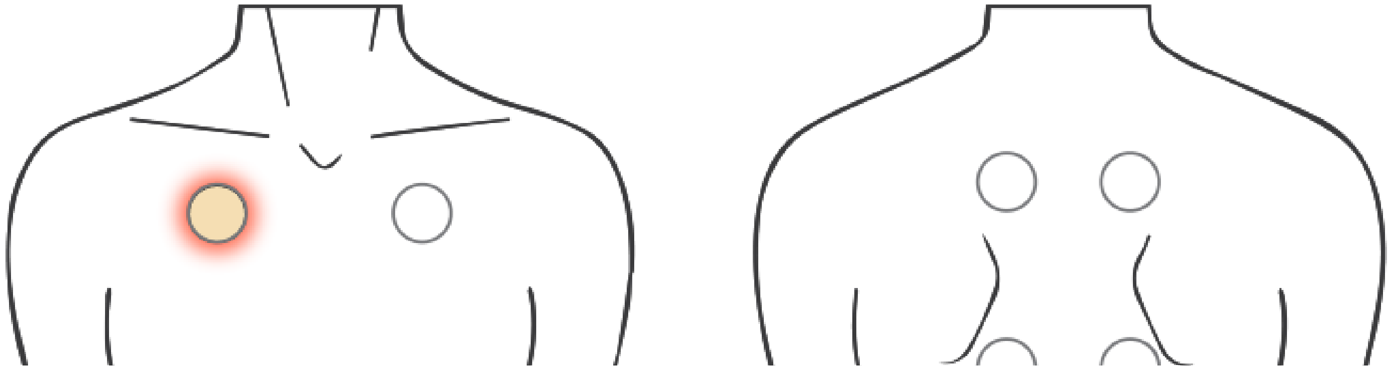

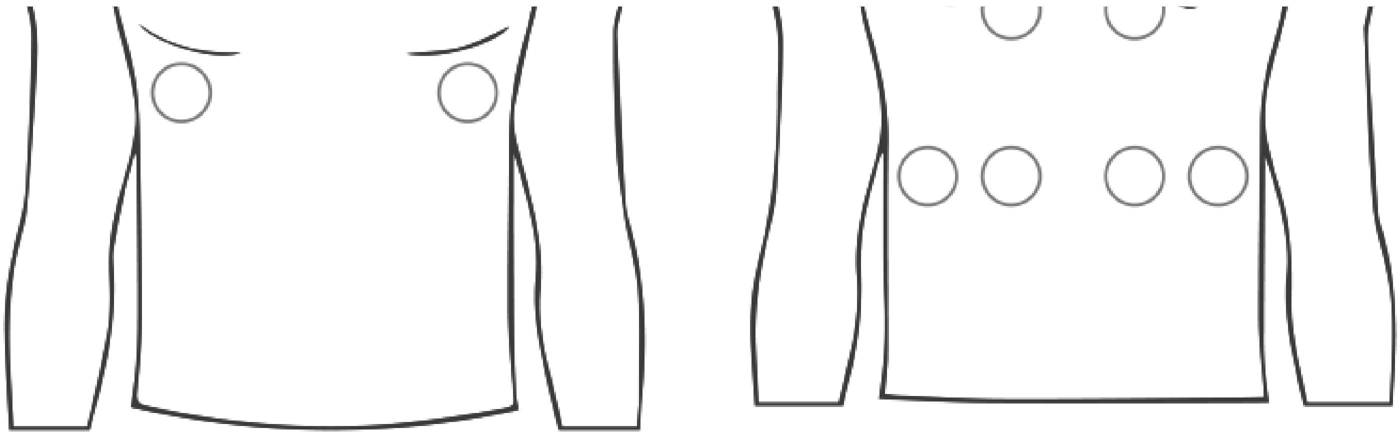

- ☐ Szmer pęcherzykowy prawidłowy
- ☐ Szmer pęcherzykowy ściszony
- ☐ Szmer pęcherzykowy zaostrzony
- ☐ Szmer oskrzelowy prawidłowy
- ☐ Szmer oskrzelowy patologiczny (w nieprawidłowym miejscu)
- ☐ Rzężenia drobnobańkowe
- ☐ Rzężenia średniobańkowe
- ☐ Rzężenia grubobańkowe
- ☐ Trzeszczenia
- ☐ Świsty wdechowe
- ☐ Świsty wydechowe
- ☐ Stridor
- ☐ Wydłużenie fazy wydechowej
- ☐ Skrzeczenia
- ☐ Tarcie opłucnowe
- ☐ Furczenia
- ☐ Uwagi

---

*

Pacjent 17

Wiek: 17 lat

Wzrost: 150 cm

Waga: 50 kg

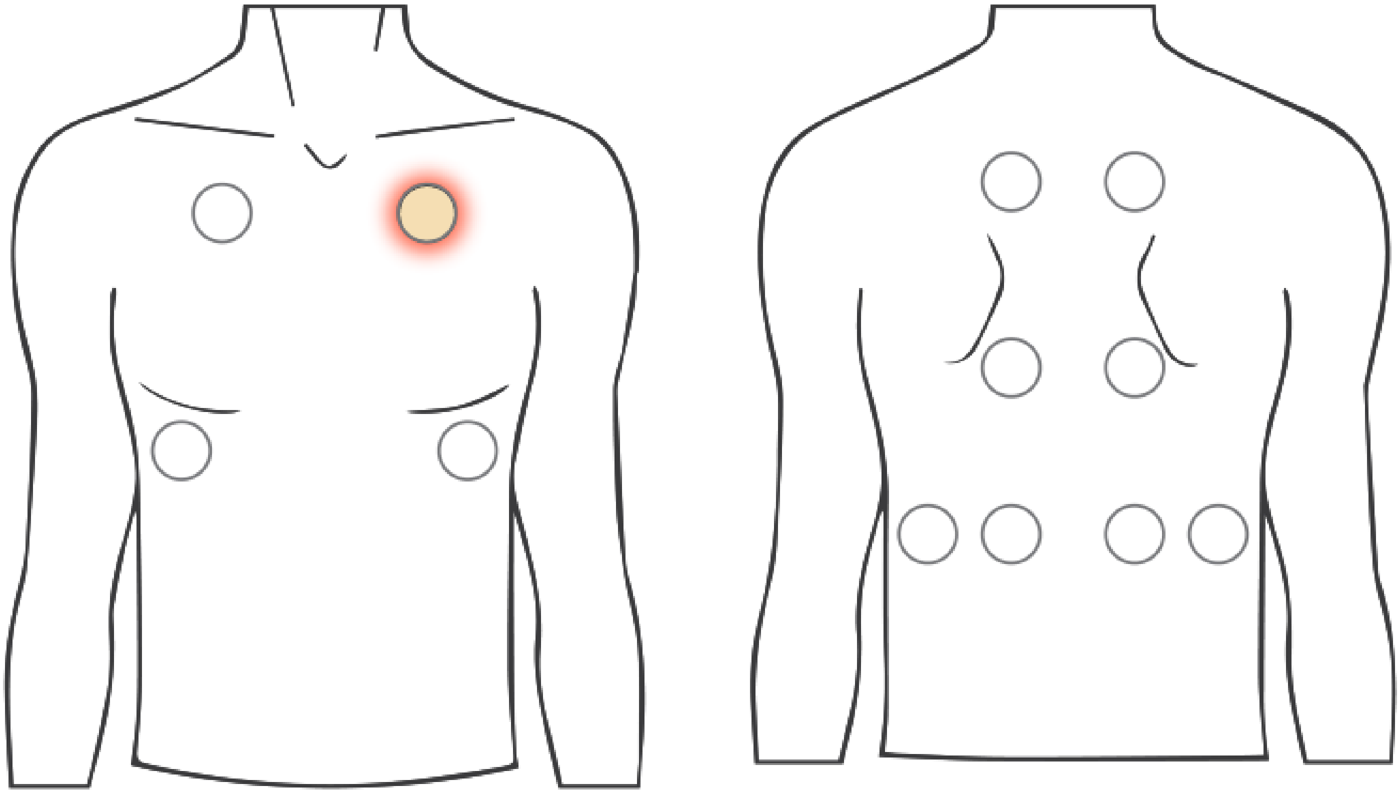

☐ Szmer pęcherzykowy prawidłowy

☐ Szmer pęcherzykowy ściszony

☐ Szmer pęcherzykowy zaostrzony

☐ Szmer oskrzelowy prawidłowy

☐ Szmer oskrzelowy patologiczny (w nieprawidłowym miejscu)

☐ Rzężenia drobnobańkowe

☐ Rzężenia średniobańkowe

☐ Rzężenia grubobańkowe

☐ Trzeszczenia

☐ Świsty wdechowe

☐ Świsty wydechowe

☐ Stridor

☐ Wydłużenie fazy wydechowej

☐ Skrzeczenia

☐ Tarcie opłucnowe

☐ Furczenia

☐ Uwagi

---

*

Pacjent 18
Wiek: 18 lat
Wzrost: 179 cm
Waga: 60 kg

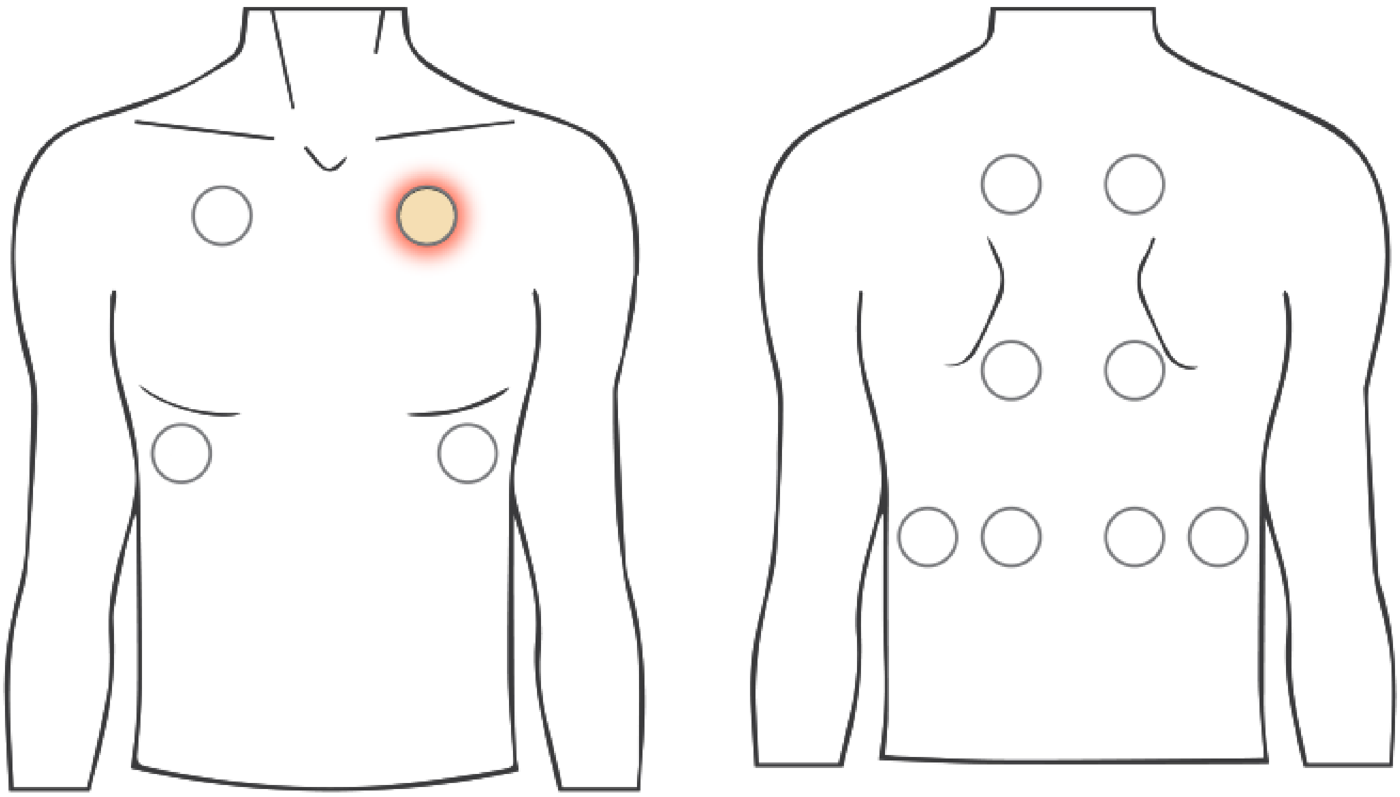

☐ Szmer pęcherzykowy prawidłowy

☐ Szmer pęcherzykowy ściszony

☐ Szmer pęcherzykowy zaostrzony

☐ Szmer oskrzelowy prawidłowy

☐ Szmer oskrzelowy patologiczny (w nieprawidłowym miejscu)

☐ Rzężenia drobnobańkowe

☐ Rzężenia średniobańkowe

☐ Rzężenia grubobańkowe

QuestionPro

☐ Trzeszczenia

☐ Świsty wdechowe

☐ Świsty wydechowe

☐ Stridor

☐ Wydłużenie fazy wydechowej

☐ Skrzeczenia

☐ Tarcie opłucnowe

☐ Furczenia

☐ Uwagi

---

*

Pacjent 19
Wiek: 2 lata
Wzrost: 84 cm
Waga: 11 kg

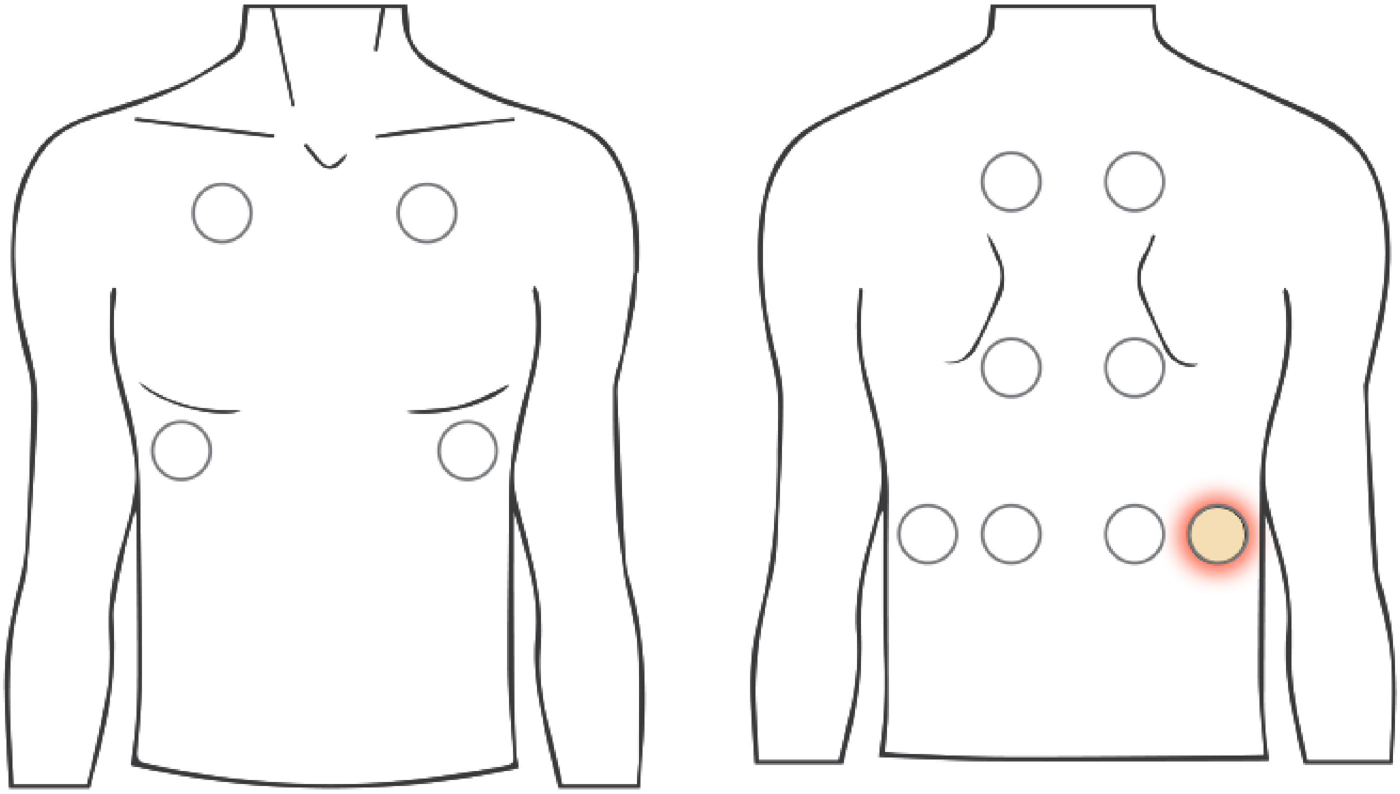

☐ Szmer pęcherzykowy prawidłowy

☐ Szmer pęcherzykowy ściszony

☐ Szmer pęcherzykowy zaostrzony

☐ Szmer oskrzelowy prawidłowy

☐ Szmer oskrzelowy patologiczny (w nieprawidłowym miejscu)

☐ Rzężenia drobnobańkowe

☐ Rzężenia średniobańkowe

☐ Rzężenia grubobańkowe

☐ Trzeszczenia

☐ Świsty wdechowe

☐ Świsty wydechowe

☐ Stridor

☐ Wydłużenie fazy wydechowej

☐ Skrzeczenia

☐ Tarcie opłucnowe

☐ Furczenia

☐ Uwagi

---

*

Pacjent 20

Wiek: 6 lat

Wzrost: 118 cm

Waga: 23 kg

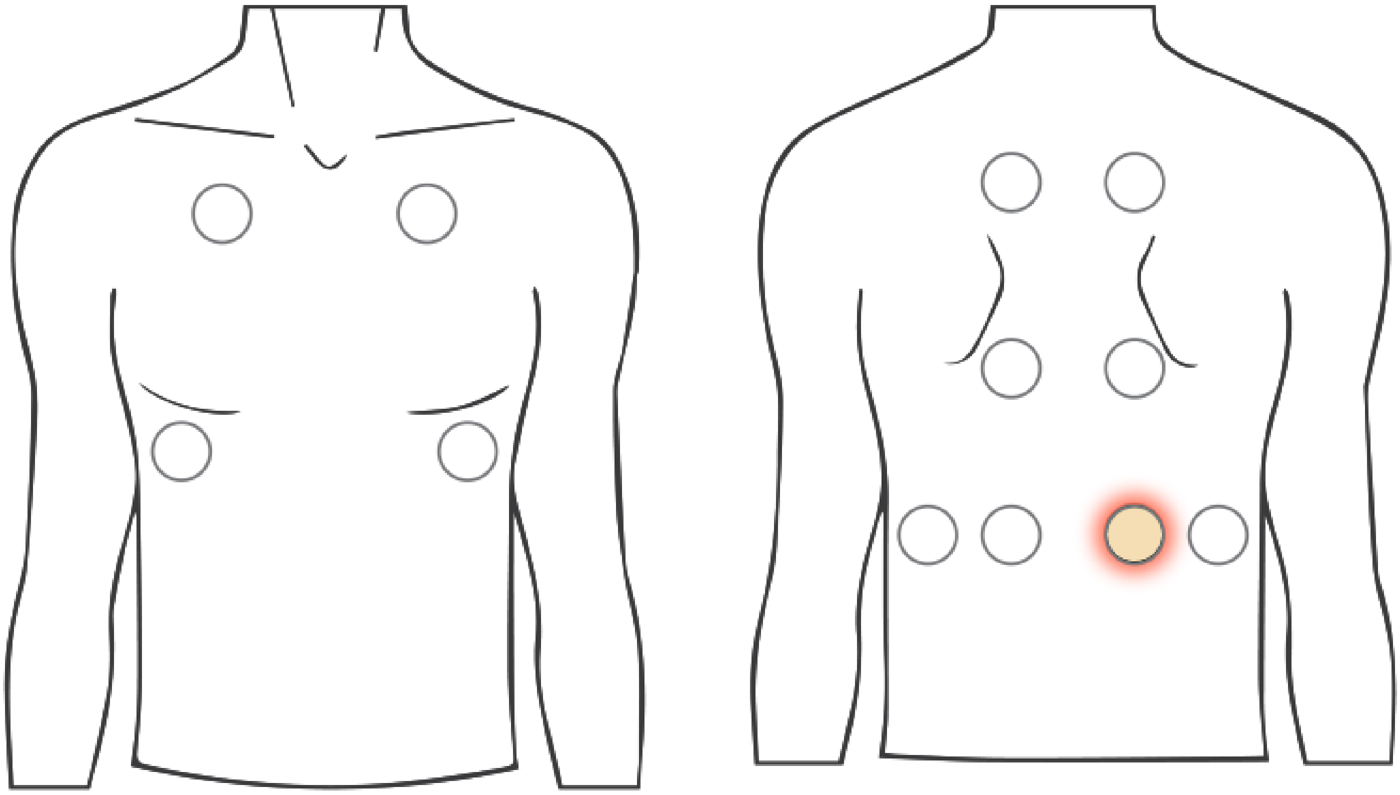

☐ Szmer pęcherzykowy prawidłowy

☐ Szmer pęcherzykowy ściszony

☐ Szmer pęcherzykowy zaostrzony

☐ Szmer oskrzelowy prawidłowy

☐ Szmer oskrzelowy patologiczny (w nieprawidłowym miejscu)

☐ Rzężenia drobnobańkowe

☐ Rzężenia średniobańkowe

☐ Rzężenia grubobańkowe

☐ Trzeszczenia

☐ Świsty wdechowe

☐ Świsty wydechowe

☐ Stridor

☐ Wydłużenie fazy wydechowej

☐ Skrzeczenia

☐ Tarcie opłucnowe

☐ Furczenia

☐ Uwagi

*

Pacjent 21

Wiek: 35 lat
Wzrost: 180 cm
Waga: 74 kg

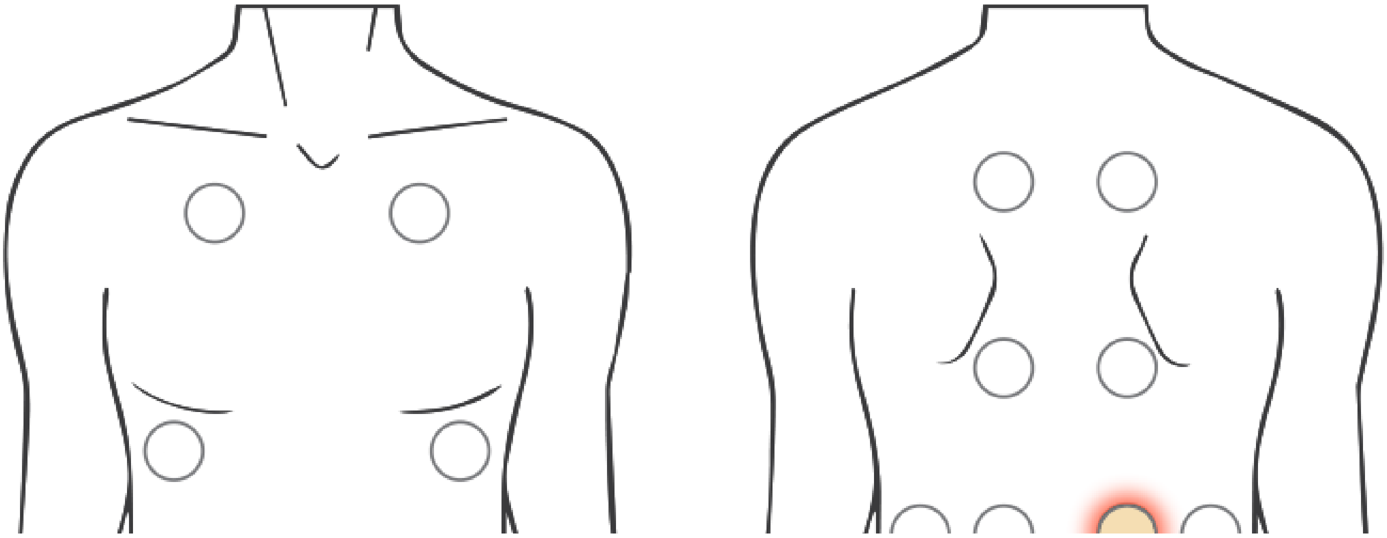

QuestionPro

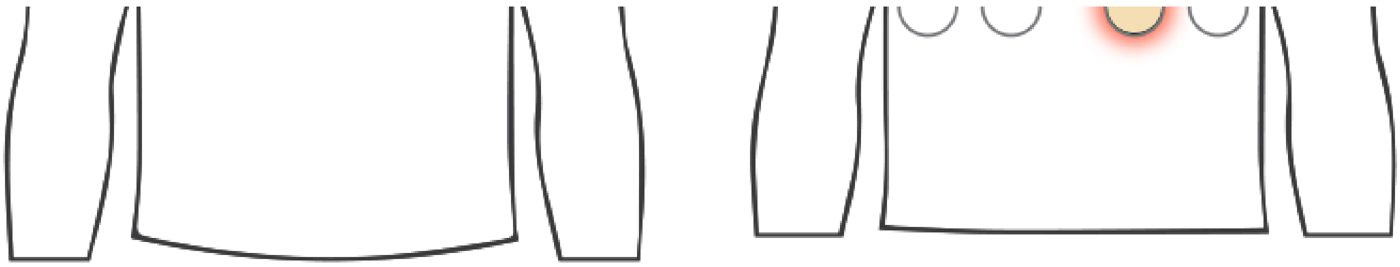

- ☐ Szmer pęcherzykowy prawidłowy
- ☐ Szmer pęcherzykowy ściszony
- ☐ Szmer pęcherzykowy zaostrzony
- ☐ Szmer oskrzelowy prawidłowy
- ☐ Szmer oskrzelowy patologiczny (w nieprawidłowym miejscu)
- ☐ Rzężenia drobnobańkowe
- ☐ Rzężenia średniobańkowe
- ☐ Rzężenia grubobańkowe
- ☐ Trzeszczenia
- ☐ Świsty wdechowe
- ☐ Świsty wydechowe
- ☐ Stridor
- ☐ Wydłużenie fazy wydechowej
- ☐ Skrzeczenia
- ☐ Tarcie opłucnowe
- ☐ Furczenia
- ☐ Uwagi

---

*

Pacjent 22
Wiek: 56 lat
Wzrost: 162 cm
Waga: 53 kg

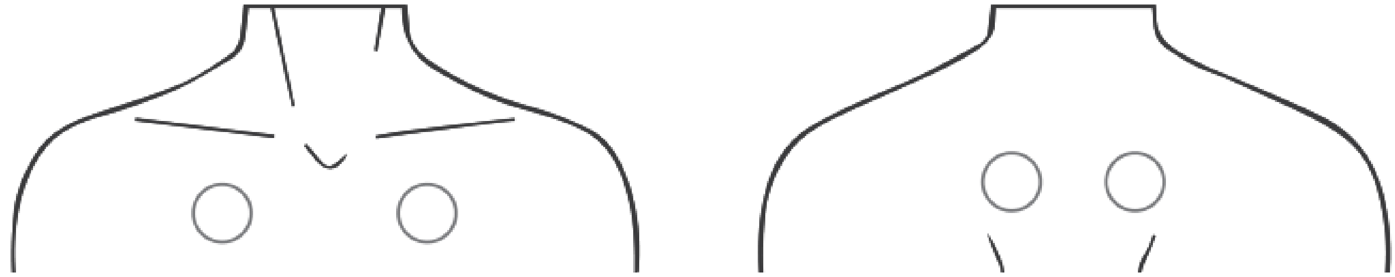

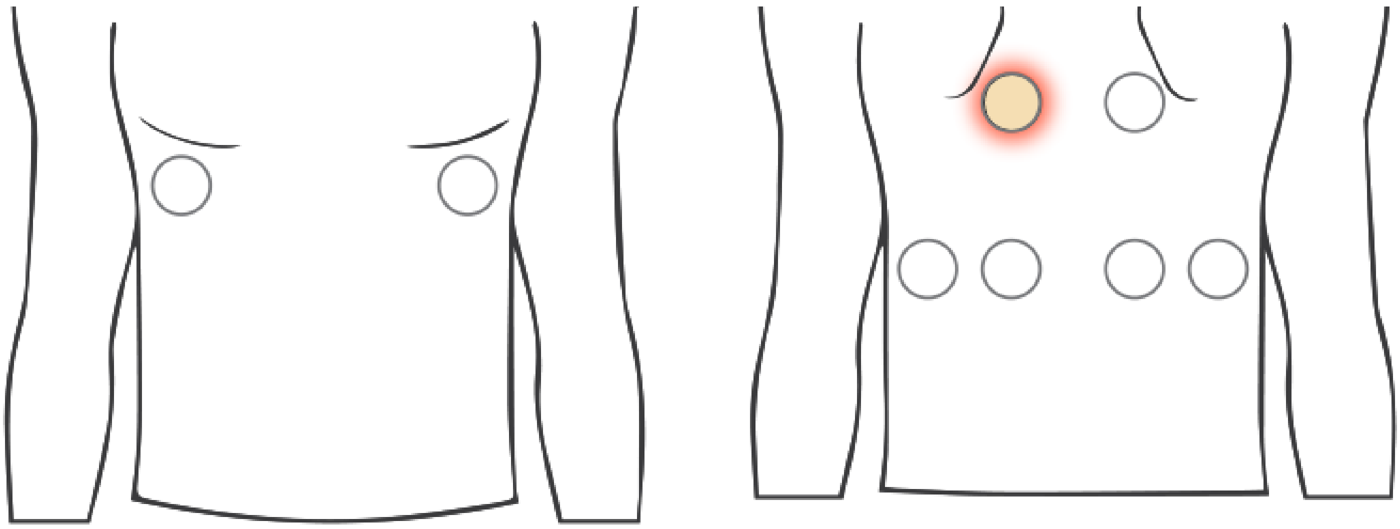

☐ Szmer pęcherzykowy prawidłowy

☐ Szmer pęcherzykowy ściszony

☐ Szmer pęcherzykowy zaostrzony

☐ Szmer oskrzelowy prawidłowy

☐ Szmer oskrzelowy patologiczny (w nieprawidłowym miejscu)

☐ Rzężenia drobnobańkowe

☐ Rzężenia średniobańkowe

☐ Rzężenia grubobańkowe

☐ Trzeszczenia

☐ Świsty wdechowe

☐ Świsty wydechowe

☐ Stridor

☐ Wydłużenie fazy wydechowej

☐ Skrzeczenia

☐ Tarcie opłucnowe

☐ Furczenia

☐ Uwagi

*

Pacjent 23

Wiek: 11 lat
Wzrost: 147 cm
Waga: 32 kg

QuestionPro

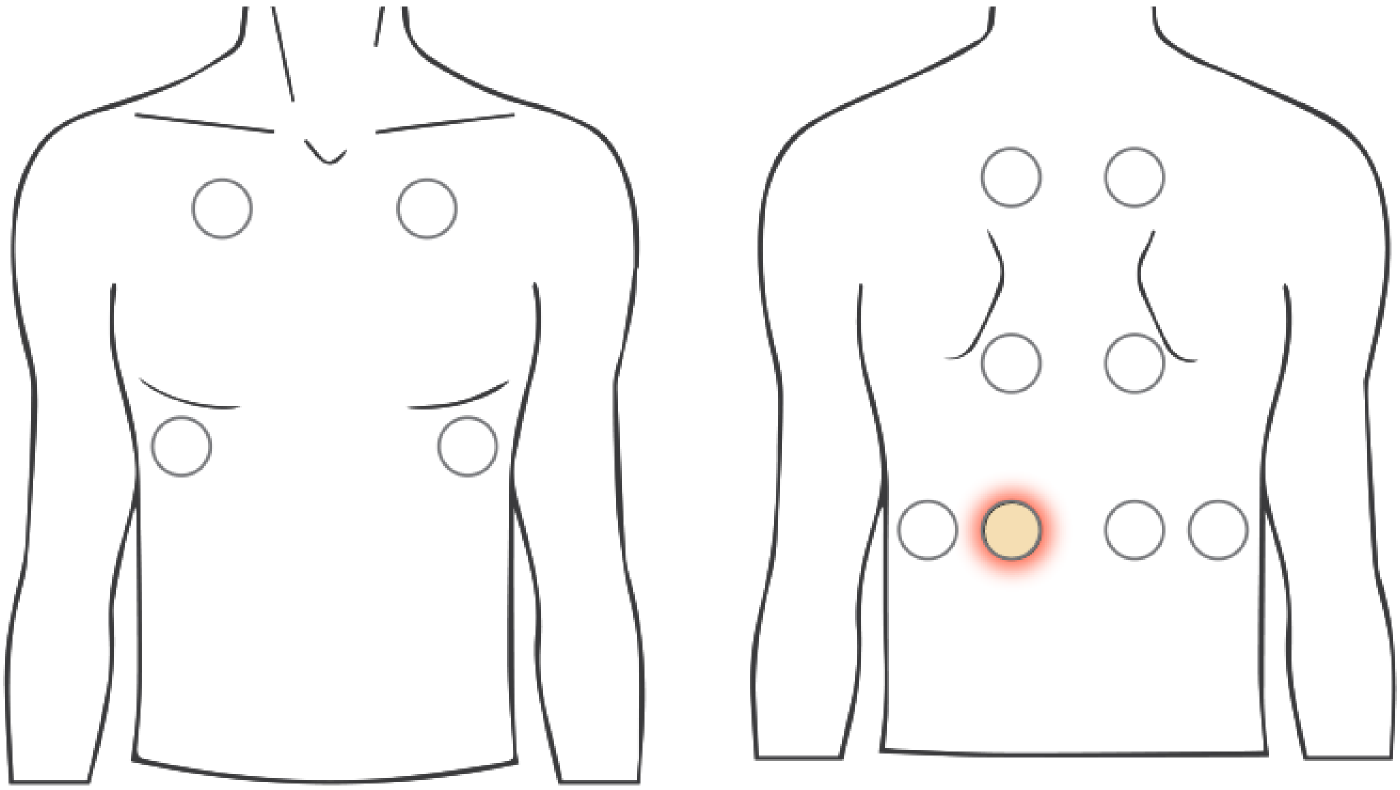

- ☐ Szmer pęcherzykowy prawidłowy
- ☐ Szmer pęcherzykowy ściszony
- ☐ Szmer pęcherzykowy zaostrzony
- ☐ Szmer oskrzelowy prawidłowy
- ☐ Szmer oskrzelowy patologiczny (w nieprawidłowym miejscu)
- ☐ Rzężenia drobnobańkowe
- ☐ Rzężenia średniobańkowe
- ☐ Rzężenia grubobańkowe
- ☐ Trzeszczenia
- ☐ Świsty wdechowe
- ☐ Świsty wydechowe
- ☐ Stridor
- ☐ Wydłużenie fazy wydechowej
- ☐ Skrzeczenia
- ☐ Tarcie opłucnowe
- ☐ Furczenia
- ☐ Uwagi

QuestionPro

*

Pacjent 24

Wiek: 14 lat

Wzrost: 150 cm

Waga: 35 kg

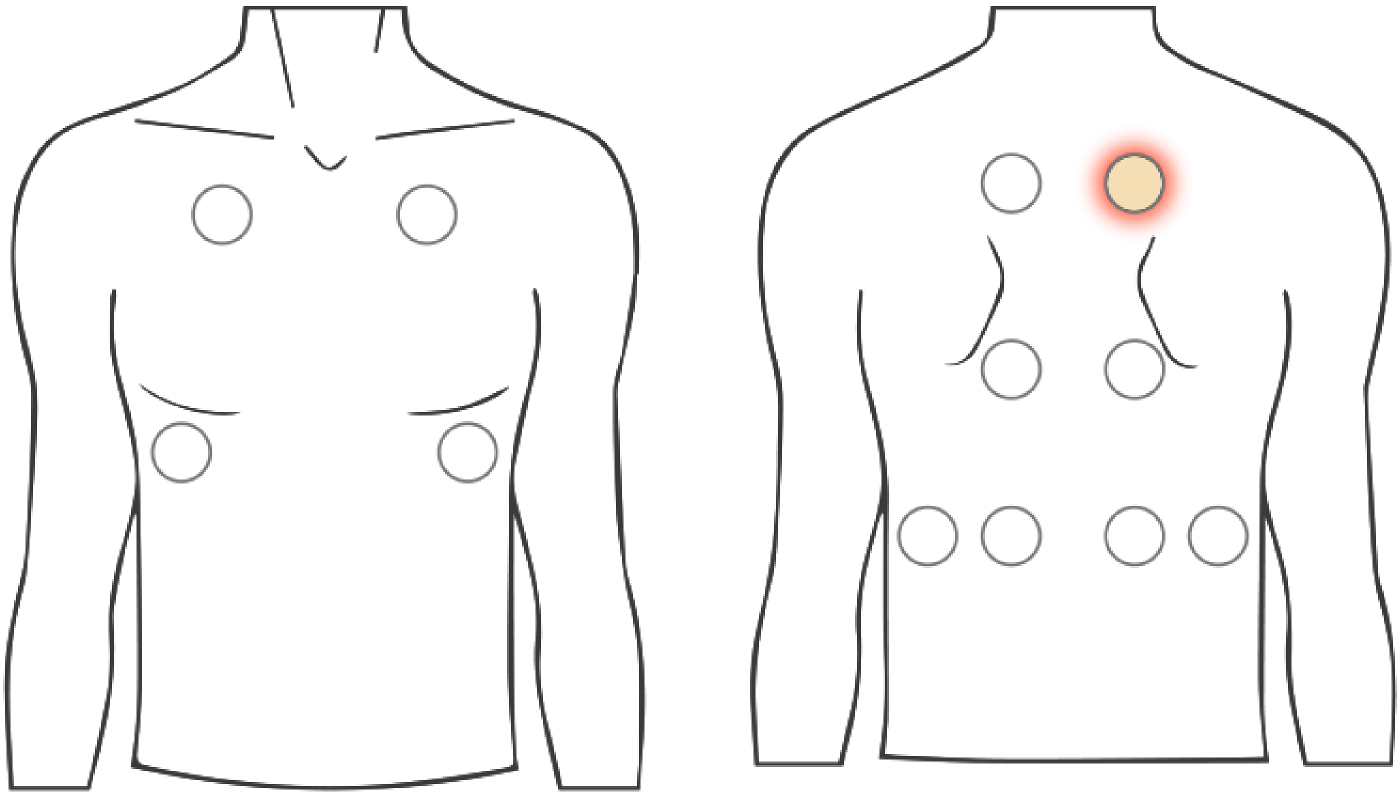

- ☐ Szmer pęcherzykowy prawidłowy
- ☐ Szmer pęcherzykowy ściszony
- ☐ Szmer pęcherzykowy zaostrzony
- ☐ Szmer oskrzelowy prawidłowy
- ☐ Szmer oskrzelowy patologiczny (w nieprawidłowym miejscu)
- ☐ Rzężenia drobnobańkowe
- ☐ Rzężenia średniobańkowe
- ☐ Rzężenia grubobańkowe
- ☐ Trzeszczenia
- ☐ Świsty wdechowe

- ☐ Świsty wydechowe
- ☐ Stridor
- ☐ Wydłużenie fazy wydechowej
- ☐ Skrzeczenia
- ☐ Tarcie opłucnowe
- ☐ Furczenia
- ☐ Uwagi

*** Proszę zaznaczyć jakie słuchawki użył Pan/Pani do odsłuchania nagrań. Jeśli znana jest firma i model słuchawek, proszę wpisać w polu "model słuchawek"**

- ☐ słuchawki nauszne
- ☐ słuchawki douszne
- ☐ model słuchawek
