# Supplementary material for: The accuracy of lung auscultation in the practice of physicians and medical students
Source: PLoS One. 2019 Aug 12;14(8):e0220606. doi: 10.1371/journal.pone.0220606 (PMC6690530; doi:10.1371/journal.pone.0220606)
Supplement: S2 File — (PDF) [file pone.0220606.s003.pdf]

Survey: Sounds of auscultation

Survey for doctors and medical students for the research about the auscultation of the respiratory system.

Please enter your e-mail. The field is not mandatory.

* **What is your specialization?**

- ☐ pediatrics
- ☐ currently training to specialize in pediatrics
- ☐ neonatology
- ☐ currently training to specialize in neonatology
- ☐ pulmonology
- ☐ currently training to specialize in pulmonology
- ☐ internal medicine
- ☐ currently training to specialize in internal medicine
- ☐ family medicine
- ☐ currently training to specialize in family medicine
- ☐ cardiac surgery
- ☐ currently training to specialize in cardiac surgery
- ☐ anesthesiology and intensive care
- ☐ currently training to specialize in anesthesiology and intensive care
- ☐ medical student (enter your year in the "other" box)
- ☐ other

* **The year of graduation (if you are a student please enter your planned year of graduation).**

* **University you graduated from (if you are a student, enter the name of the University where you are currently studying).**

On a scale from 0 to 5 (where 0 = "very poorly" and 5 = "very well"), how do you evaluate your skills in respiratory system auscultation?

	0	1	2	3	4	5
* a child	○	○	○	○	○	○
* an adult	○	○	○	○	○	○

Please indicate how much you agree with the following statements.

	strongly disagree	do not agree	partially agree	agree	strongly agree
* The number of hours during the studies dedicated to the auscultation of the respiratory system is INADEQUATE	○	○	○	○	○
* The number of hours dedicated to training in auscultation of the respiratory system during internship and specialization is INADEQUATE	○	○	○	○	○
* Additional training in auscultation of respiratory system for doctors in my specialization are needed	○	○	○	○	○
* The names associated with the auscultatory sounds of the respiratory system are inconsistent and need to be organised	○	○	○	○	○

How often do you practice auscultation.

	everyday	few times per week	at least once per week	at least once per month	fewer than once per month	I don't do auscultation
* a child	○	○	○	○	○	○
* an adult	○	○	○	○	○	○

*** Do you have a musical background?**

☐ no

☐ music school

☐ choir

☐ other

[ ]

Do you use an electronic stethoscope in your work?

○ yes

○ no

*** During lungs auscultation, do you use:**

☐ the diaphragm of stethoscope

☐ the bell of stethoscope

☐ other (what kind?)

[ ]

Looking at the illustration below, please select the points where you usually auscultate a patient.

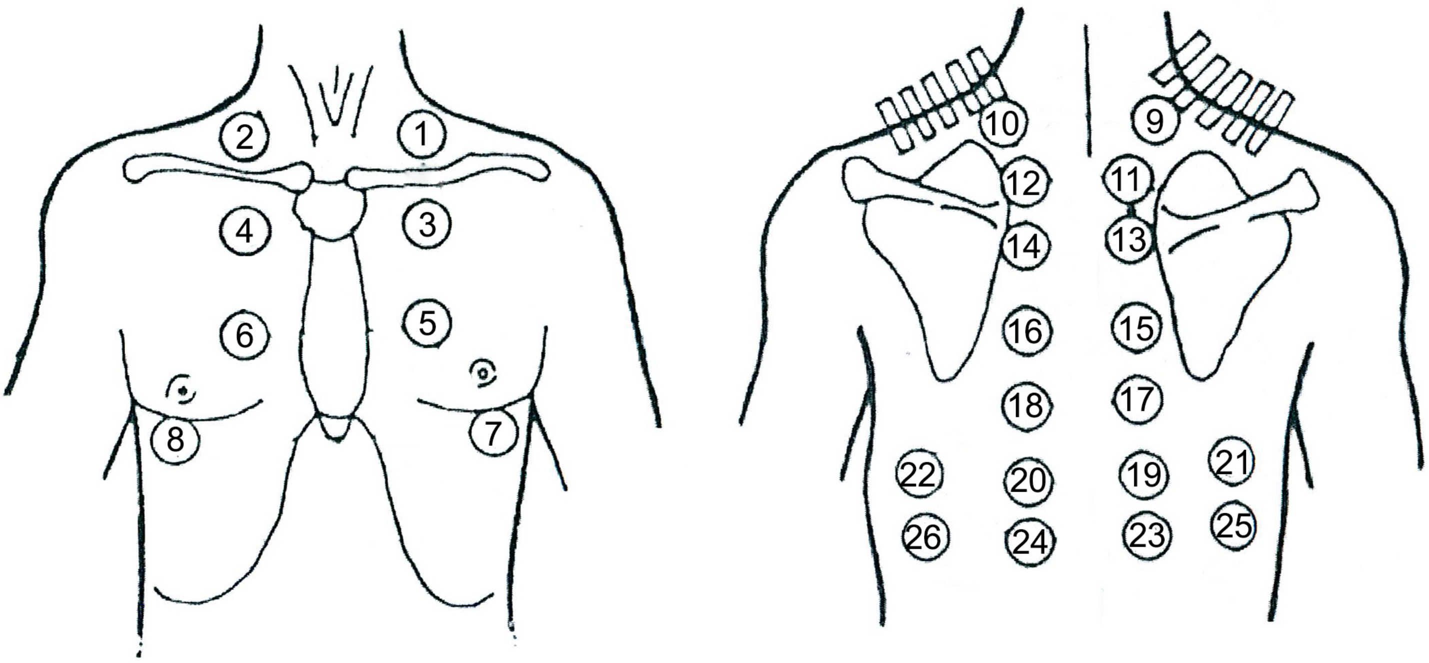

	1	2	3	4	5	6	7	8	9	10	11	12	13	14	15	16	17	18	19	20	21	22	23	24	25	26
* a child	☐	☐	☐	☐	☐	☐	☐	☐	☐	☐	☐	☐	☐	☐	☐	☐	☐	☐	☐	☐	☐	☐	☐	☐	☐	☐
* an adult	☐	☐	☐	☐	☐	☐	☐	☐	☐	☐	☐	☐	☐	☐	☐	☐	☐	☐	☐	☐	☐	☐	☐	☐	☐	☐

The research on auscultation of the respiratory system - practical part

In this part auscultation recordings of 24 patients will be presented. Please use headphones to listen to the recordings.

For each patient the information includes age, height and weight of the patient.

The illustrations show where the stethoscope was placed and the sound recorded. Below are the recordings for you to listen to. Adjust the volume as needed. The recordings can be played repeatedly.

Please identify the respiratory sounds you can hear in each recording (multiple choice from two columns).

*

Patient 1

Age: 11 years old

Height: 147 cm

Weight: 32 kg

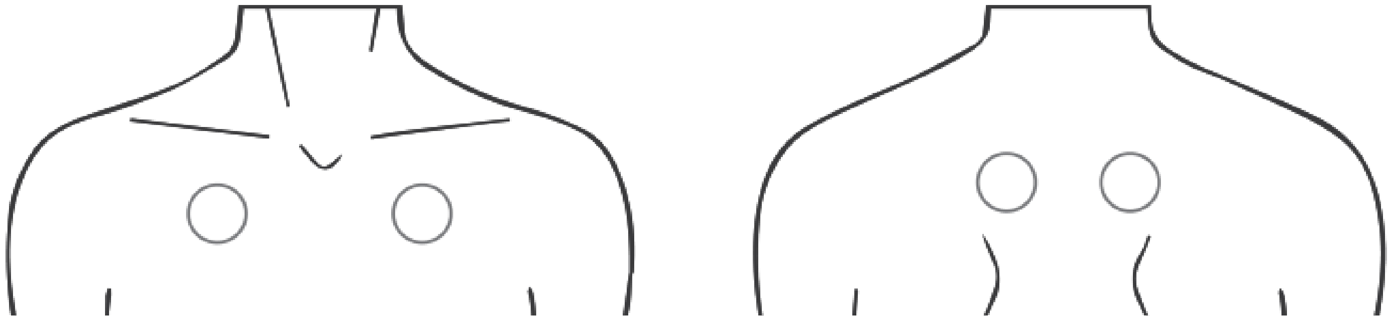

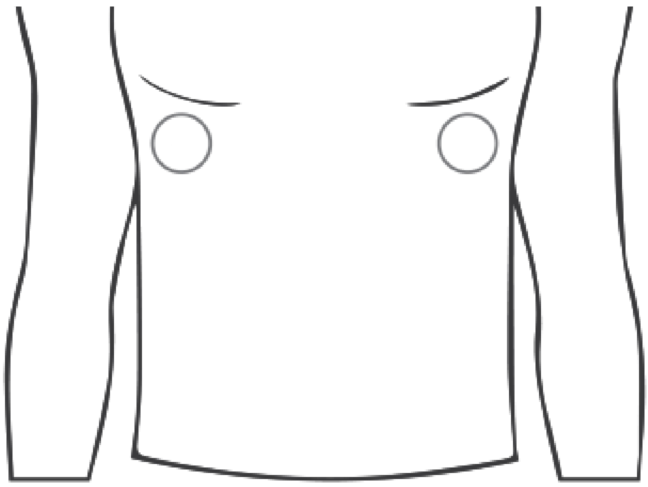

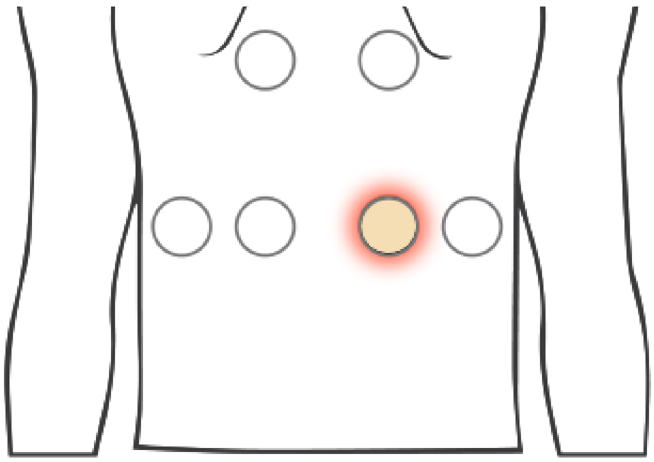

- ☐ vesicular breath sound
- ☐ louder breath sound
- ☐ abnormal bronchial sound
- ☐ medium crackles
- ☐ crepitus
- ☐ expiratory wheezes
- ☐ prolonged expiratory phase
- ☐ pleural rub
- ☐ comments
- ☐ diminished breath sound
- ☐ normal bronchial sound
- ☐ fine crackles
- ☐ coarse crackles
- ☐ inspiratory wheezes
- ☐ stridor
- ☐ squawk
- ☐ rhonchi

*

Patient 2

Age: 3 years old
Height: 96 cm
Weight: 12 kg

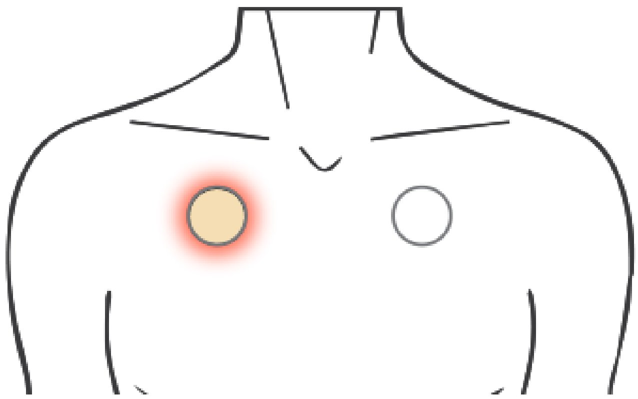

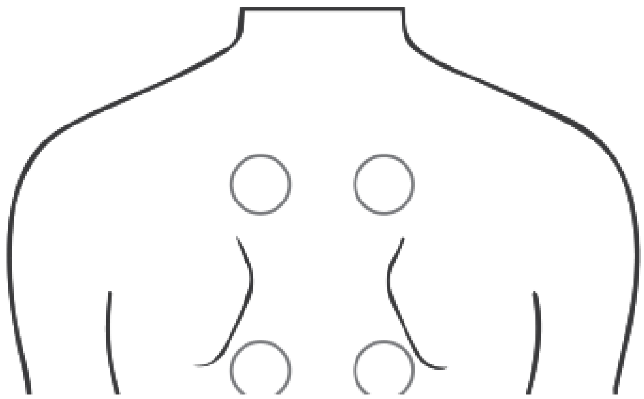

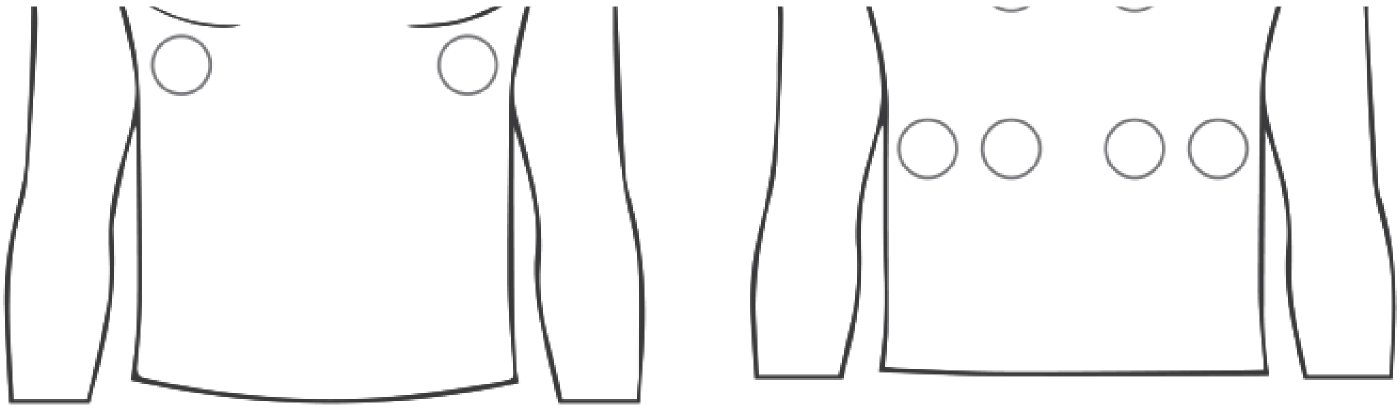

- ☐ vesicular breath sound
- ☐ louder breath sound
- ☐ abnormal bronchial sound
- ☐ medium crackles
- ☐ crepitus
- ☐ expiratory wheezes
- ☐ prolonged expiratory phase
- ☐ pleural rub
- ☐ comments
- ☐ diminished breath sound
- ☐ normal bronchial sound
- ☐ fine crackles
- ☐ coarse crackles
- ☐ inspiratory wheezes
- ☐ stridor
- ☐ squawk
- ☐ rhonchi

*
Patient 3
Age: 6 years old
Height: 118 cm
Weight: 30 kg

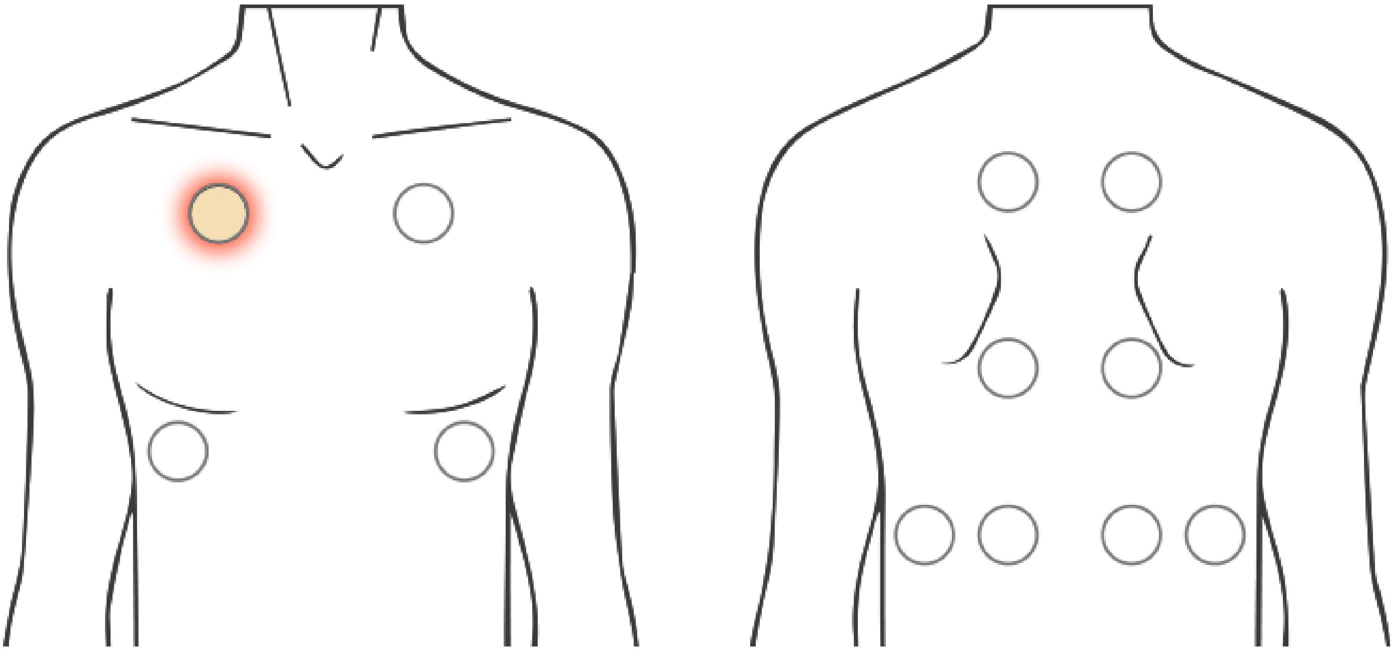

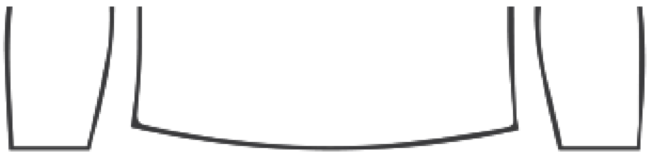

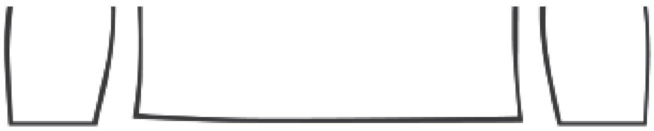

- ☐ vesicular breath sound
- ☐ louder breath sound
- ☐ abnormal bronchial sound
- ☐ medium crackles
- ☐ crepitus
- ☐ expiratory wheezes
- ☐ prolonged expiratory phase
- ☐ pleural rub
- ☐ comments
- ☐ diminished breath sound
- ☐ normal bronchial sound
- ☐ fine crackles
- ☐ coarse crackles
- ☐ inspiratory wheezes
- ☐ stridor
- ☐ squawk
- ☐ rhonchi

*

Patient 4

Age: 7 years old
Height: 115 cm
Weight: 21 kg

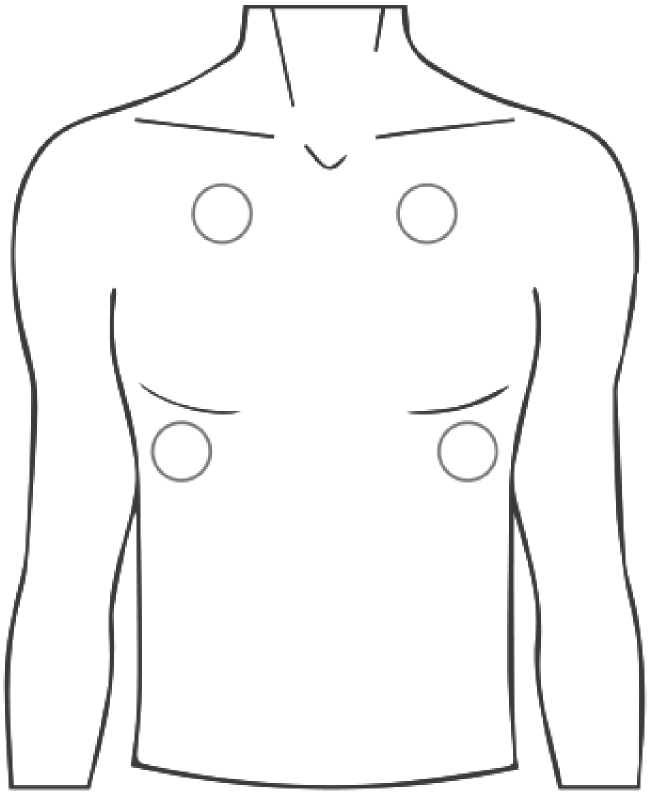

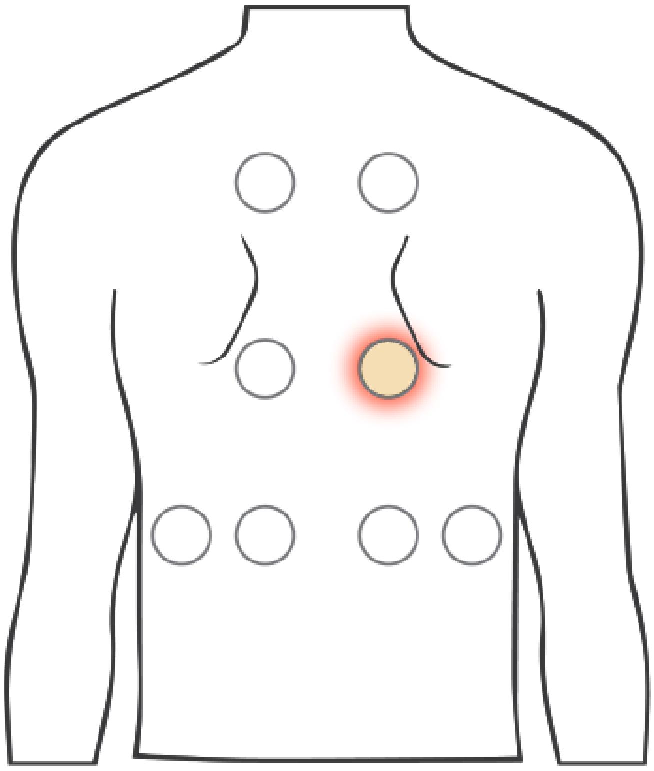

☐ vesicular breath sound

☐ diminished breath sound

☐ louder breath sound

☐ normal bronchial sound

☐ abnormal bronchial sound

☐ fine crackles

☐ medium crackles

☐ coarse crackles

☐ crepitus

☐ inspiratory wheezes

☐ expiratory wheezes

☐ stridor

☐ prolonged expiratory phase

☐ squawk

☐ pleural rub

☐ rhonchi

☐ comments

*

Patient 5

Age: 18 years old
Height: 168 cm
Weight: 50 kg

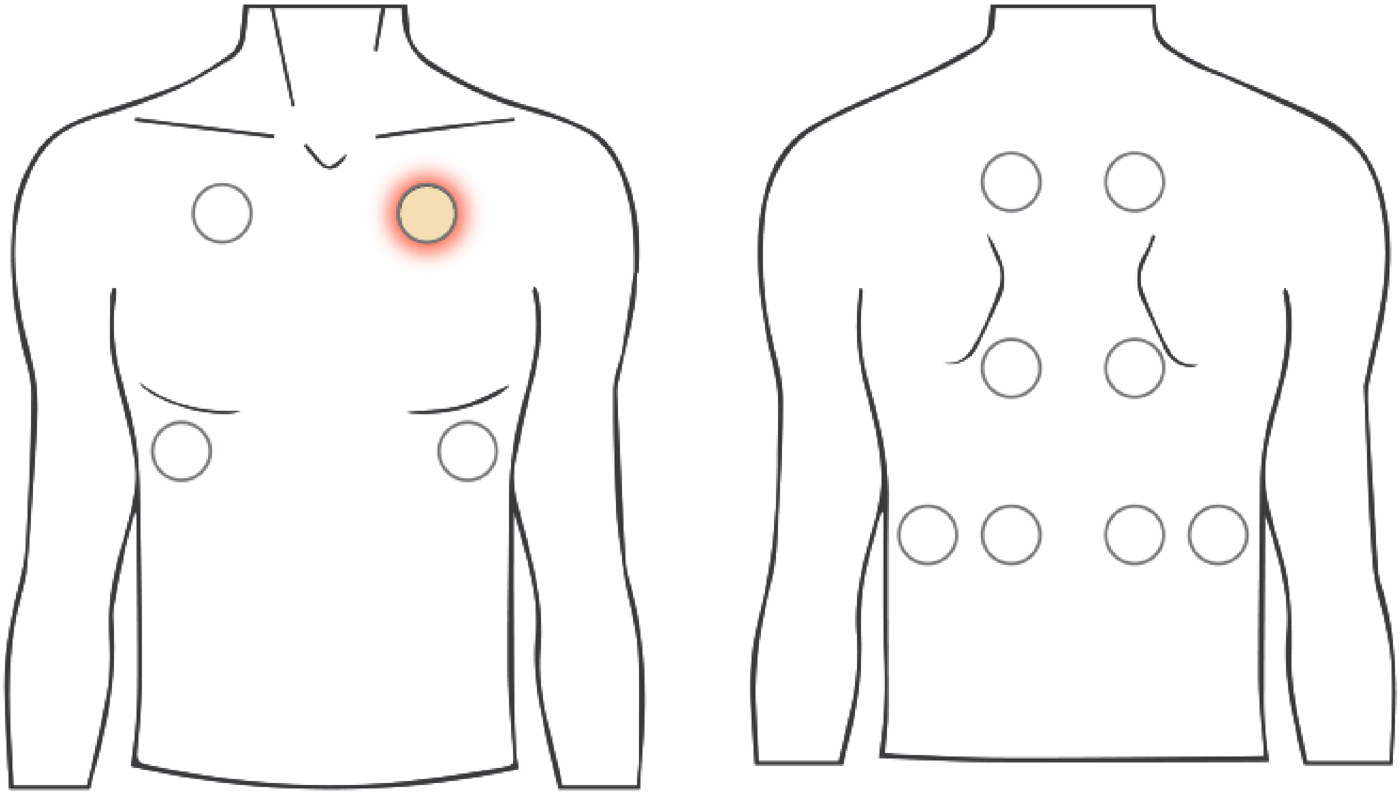

☐ vesicular breath sound

☐ diminished breath sound

☐ louder breath sound

☐ normal bronchial sound

☐ abnormal bronchial sound

☐ fine crackles

☐ medium crackles

☐ coarse crackles

QuestionPro

☐ crepitus

☐ expiratory wheezes

☐ prolonged expiratory phase

☐ pleural rub

☐ comments

☐ inspiratory wheezes

☐ stridor

☐ squawk

☐ rhonchi

*
Patient 6
Age: 5 months old
Height: 78 cm
Weight: 7 kg

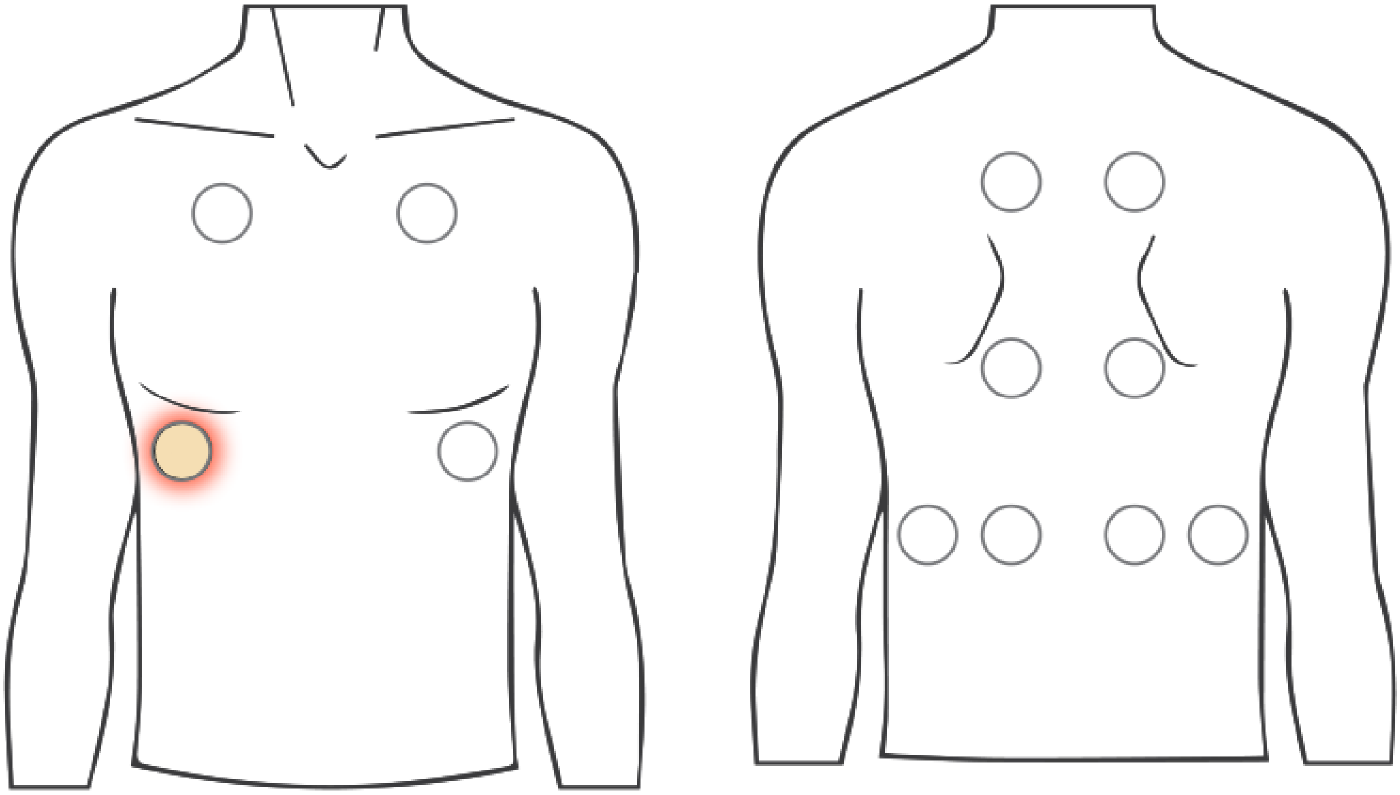

☐ vesicular breath sound

☐ louder breath sound

☐ abnormal bronchial sound

☐ medium crackles

☐ crepitus

☐ expiratory wheezes

☐ prolonged expiratory phase

☐ pleural rub

☐ diminished breath sound

☐ normal bronchial sound

☐ fine crackles

☐ coarse crackles

☐ inspiratory wheezes

☐ stridor

☐ squawk

☐ rhonchi

☐ comments

*

Patient 7

Age: 2 years old

Height: 81 cm

Weight: 10 kg

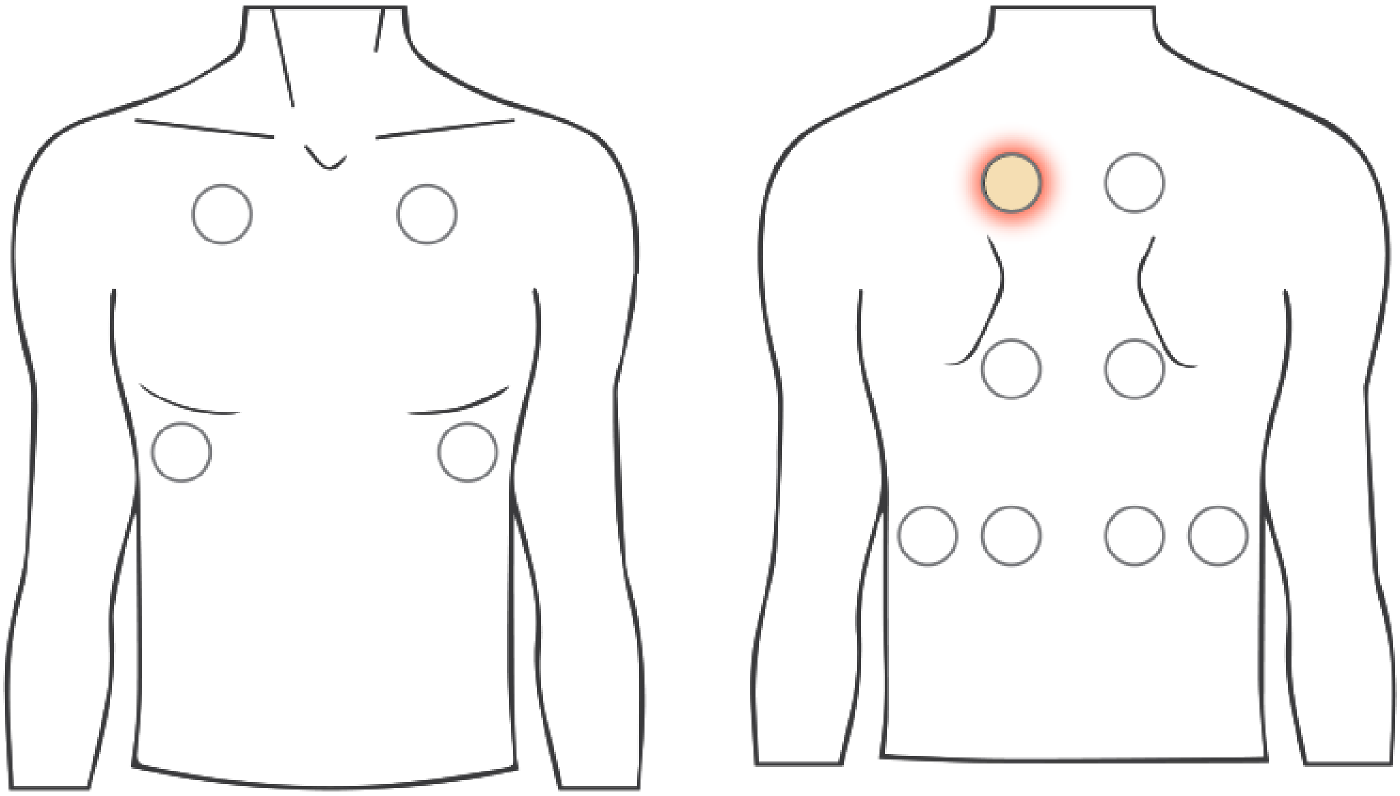

- ☐ vesicular breath sound
- ☐ louder breath sound
- ☐ abnormal bronchial sound
- ☐ medium crackles
- ☐ crepitus
- ☐ expiratory wheezes
- ☐ prolonged expiratory phase
- ☐ diminished breath sound
- ☐ normal bronchial sound
- ☐ fine crackles
- ☐ coarse crackles
- ☐ inspiratory wheezes
- ☐ stridor
- ☐ squawk

- [ ] pleural rub
- [ ] rhonchi
- [ ] comments

*

Patient 8

Age: 18 years old

Height: 179 cm

Weight: 60 kg

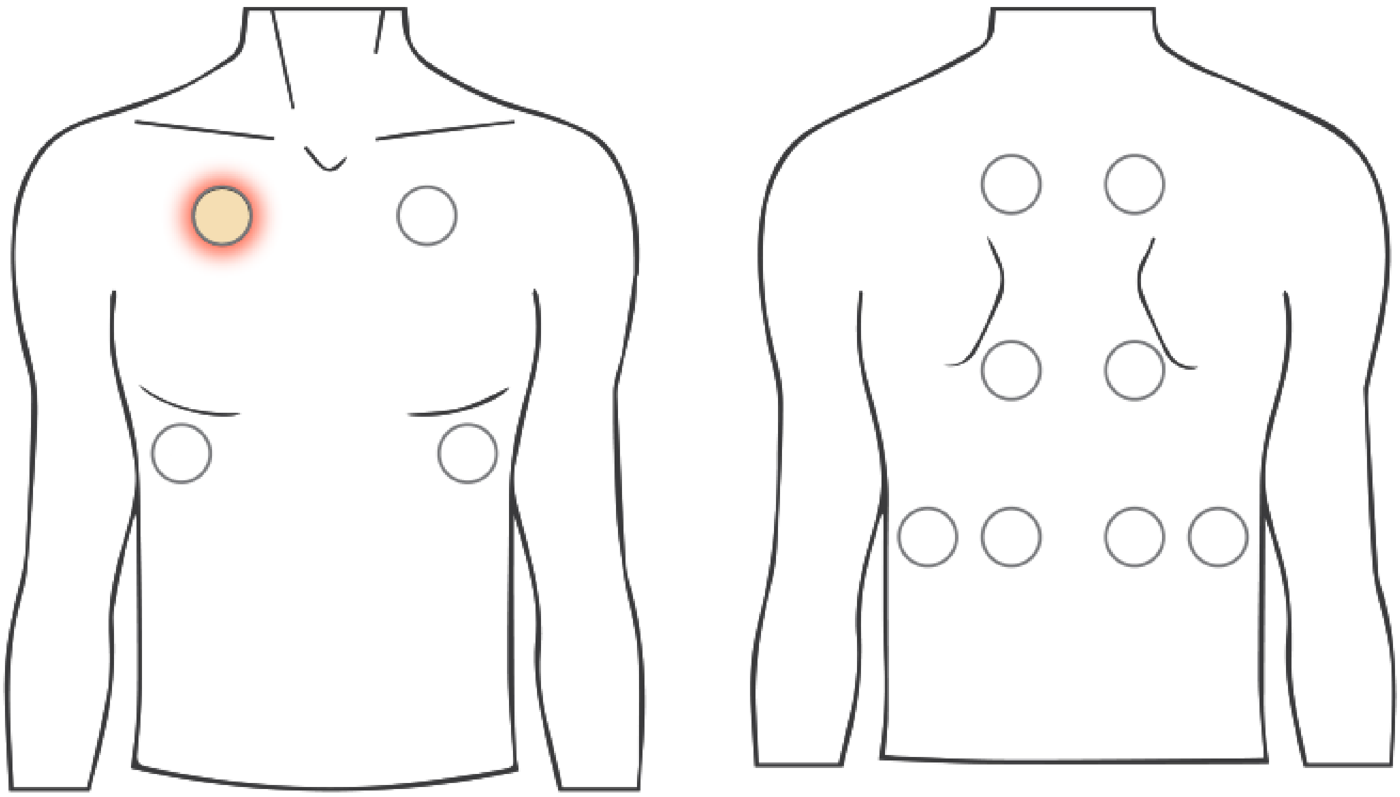

- [ ] vesicular breath sound
- [ ] diminished breath sound
- [ ] louder breath sound
- [ ] normal bronchial sound
- [ ] abnormal bronchial sound
- [ ] fine crackles
- [ ] medium crackles
- [ ] coarse crackles
- [ ] crepitus
- [ ] inspiratory wheezes
- [ ] expiratory wheezes
- [ ] stridor

☐ prolonged expiratory phase

☐ pleural rub

☐ comments

☐ squawk

☐ rhonchi

*

Patient 9

Age: 15 years old

Height: 164 cm

Weight: 42 kg

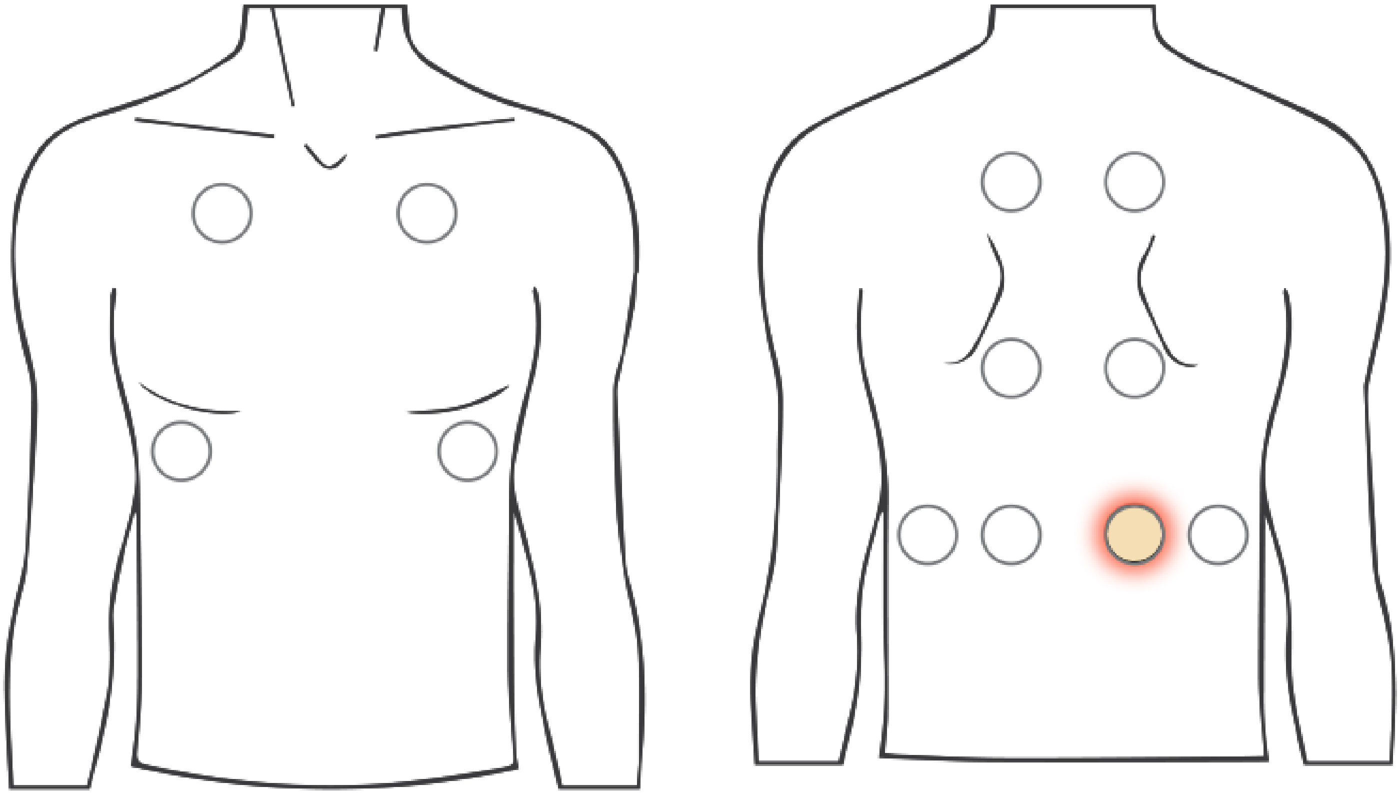

☐ vesicular breath sound

☐ louder breath sound

☐ abnormal bronchial sound

☐ medium crackles

☐ crepitus

☐ diminished breath sound

☐ normal bronchial sound

☐ fine crackles

☐ coarse crackles

☐ inspiratory wheezes

QuestionPro

- ☐ expiratory wheezes
- ☐ stridor
- ☐ prolonged expiratory phase
- ☐ squawk
- ☐ pleural rub
- ☐ rhonchi
- ☐ comments

*

Patient 10

Age: 3 years old

Height: 92 cm

Weight: 14 kg

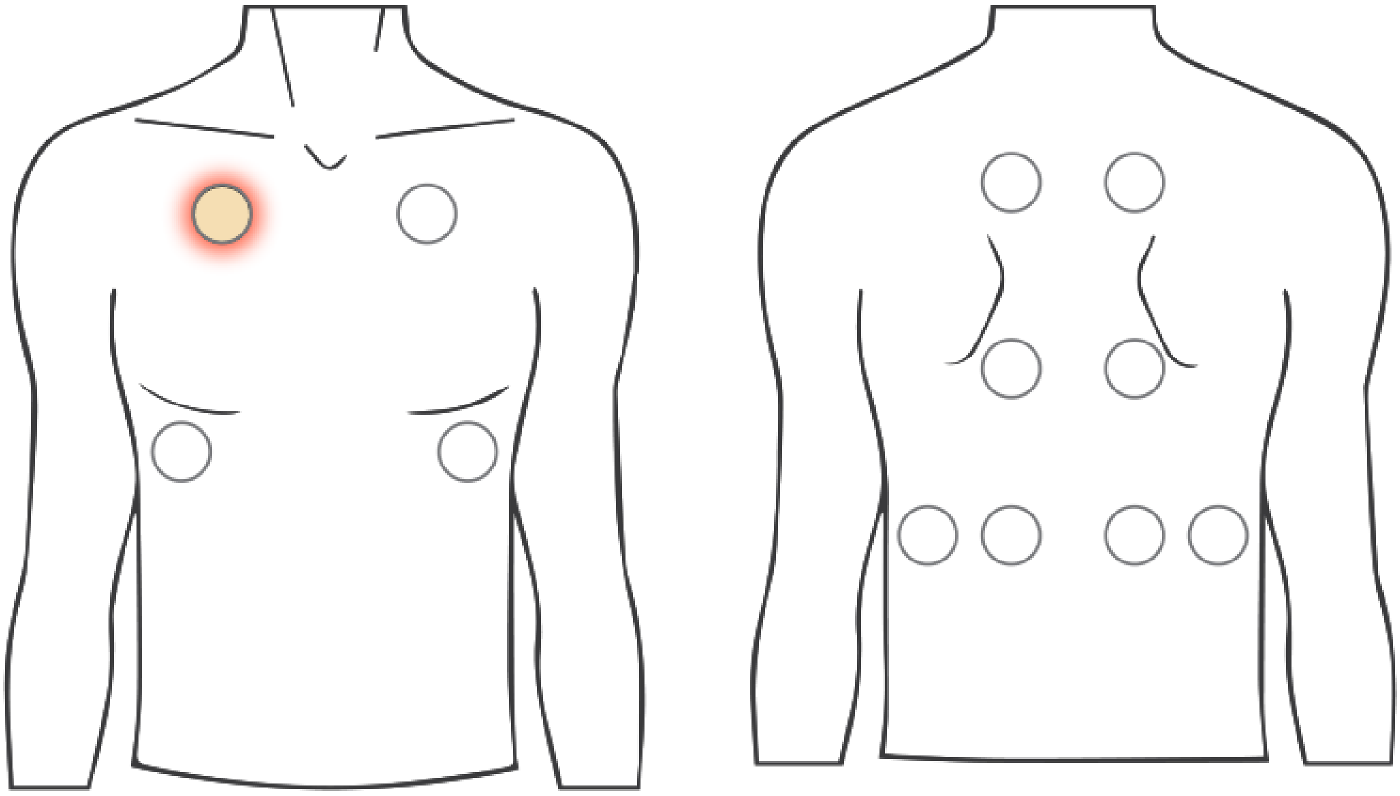

- ☐ vesicular breath sound
- ☐ diminished breath sound
- ☐ louder breath sound
- ☐ normal bronchial sound
- ☐ abnormal bronchial sound
- ☐ fine crackles
- ☐ medium crackles
- ☐ coarse crackles

- ☐ crepitus
- ☐ expiratory wheezes
- ☐ prolonged expiratory phase
- ☐ pleural rub
- ☐ comments
- ☐ inspiratory wheezes
- ☐ stridor
- ☐ squawk
- ☐ rhonchi

*

Patient 11

Age: 7 months old
Wzrost: 82 cm
Weight: 8 kg

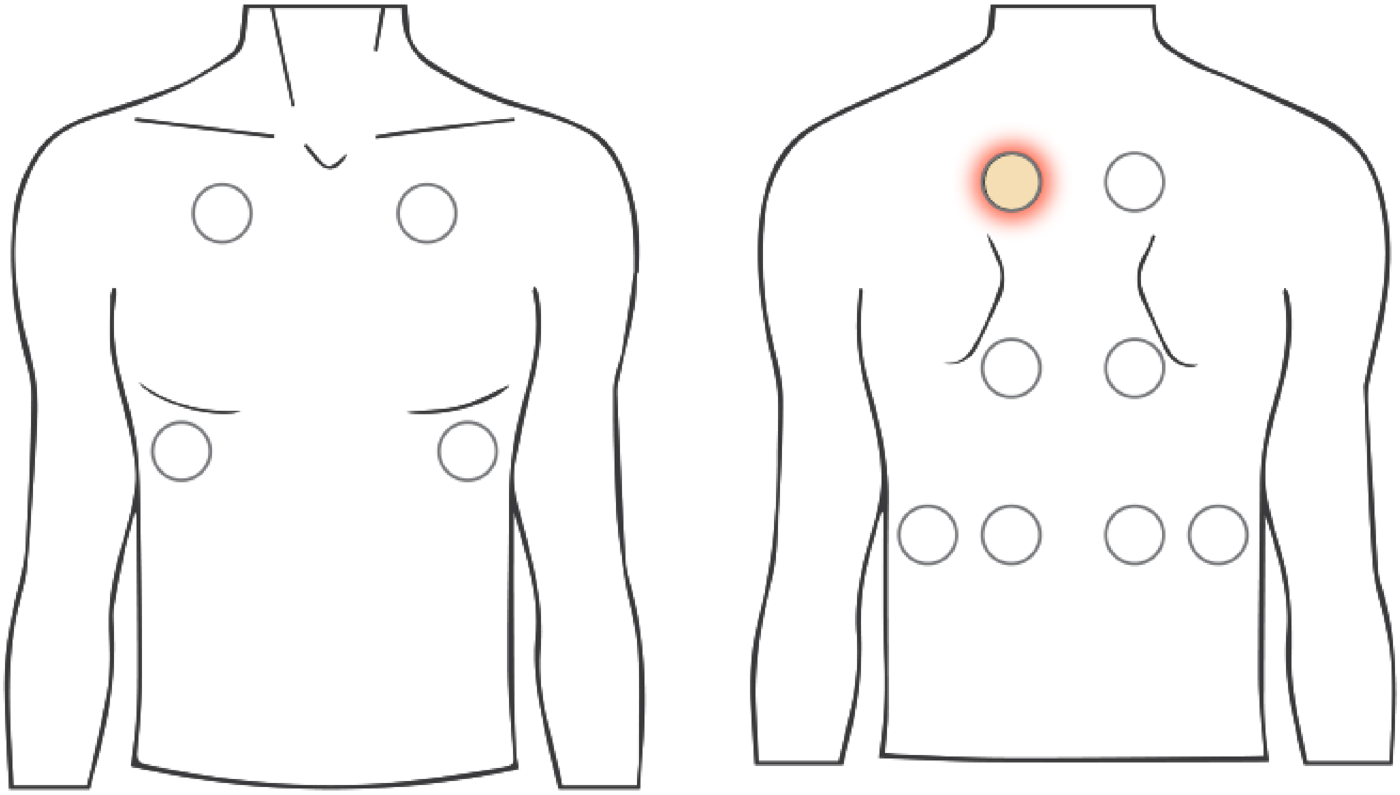

- ☐ vesicular breath sound
- ☐ louder breath sound
- ☐ abnormal bronchial sound
- ☐ medium crackles
- ☐ crepitus
- ☐ expiratory wheezes
- ☐ prolonged expiratory phase
- ☐ pleural rub
- ☐ diminished breath sound
- ☐ normal bronchial sound
- ☐ fine crackles
- ☐ coarse crackles
- ☐ inspiratory wheezes
- ☐ stridor
- ☐ squawk
- ☐ rhonchi

☐ comments

*

Patient 12

Age: 17 years old
Height: 150 cm
Weight: 50 kg

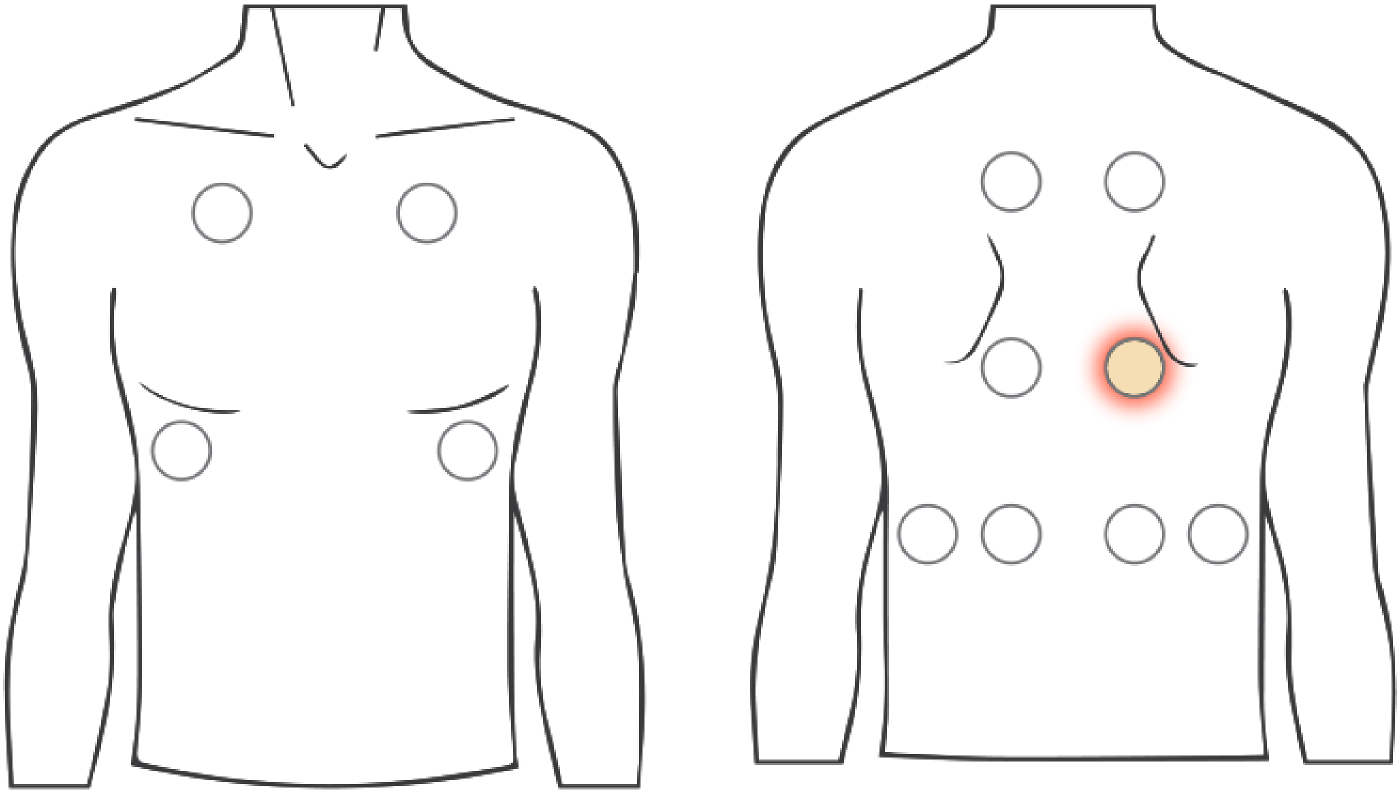

☐ vesicular breath sound

☐ louder breath sound

☐ abnormal bronchial sound

☐ medium crackles

☐ crepitus

☐ expiratory wheezes

☐ prolonged expiratory phase

☐ pleural rub

☐ comments

☐ diminished breath sound

☐ normal bronchial sound

☐ fine crackles

☐ coarse crackles

☐ inspiratory wheezes

☐ stridor

☐ squawk

☐ rhonchi

*

Patient 13

Age: 13 years old
Height: 148 cm
Weight: 33 kg

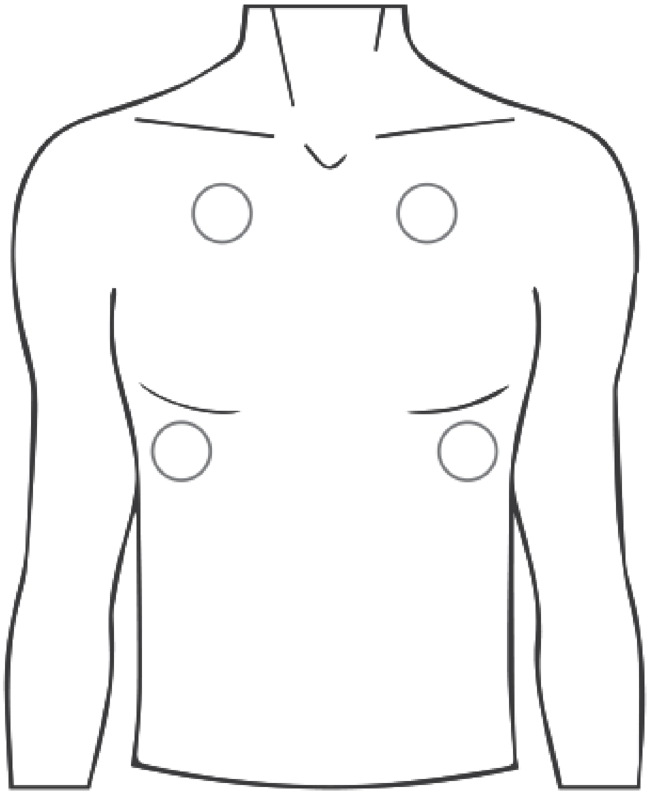

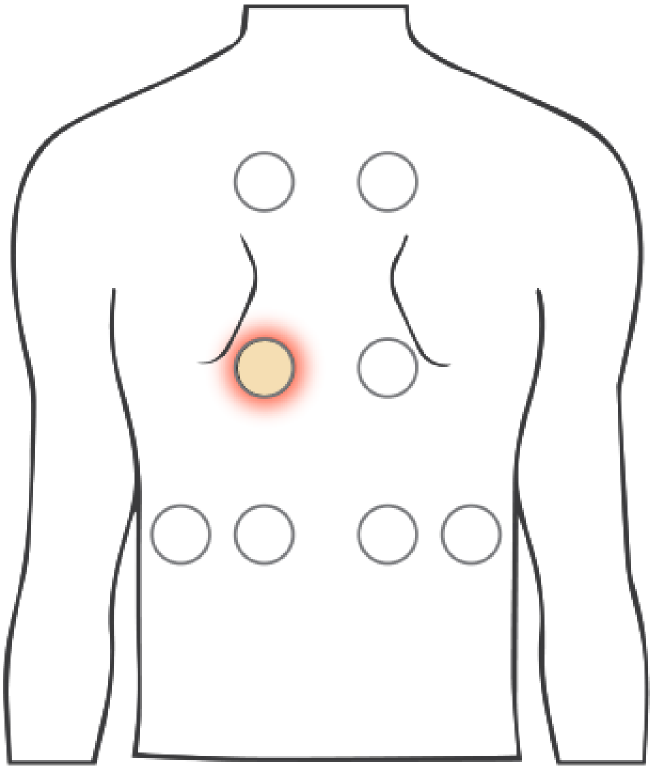

- ☐ vesicular breath sound
- ☐ louder breath sound
- ☐ abnormal bronchial sound
- ☐ medium crackles
- ☐ crepitus
- ☐ expiratory wheezes
- ☐ prolonged expiratory phase
- ☐ pleural rub
- ☐ comments
- ☐ diminished breath sound
- ☐ normal bronchial sound
- ☐ fine crackles
- ☐ coarse crackles
- ☐ inspiratory wheezes
- ☐ stridor
- ☐ squawk
- ☐ rhonchi

*

Patient 14

Age: 14 years old
Height: 150 cm
Weight: 35 kg

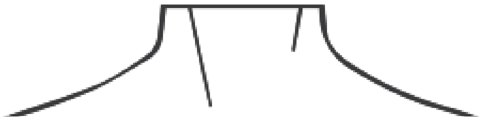

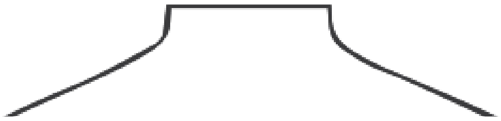

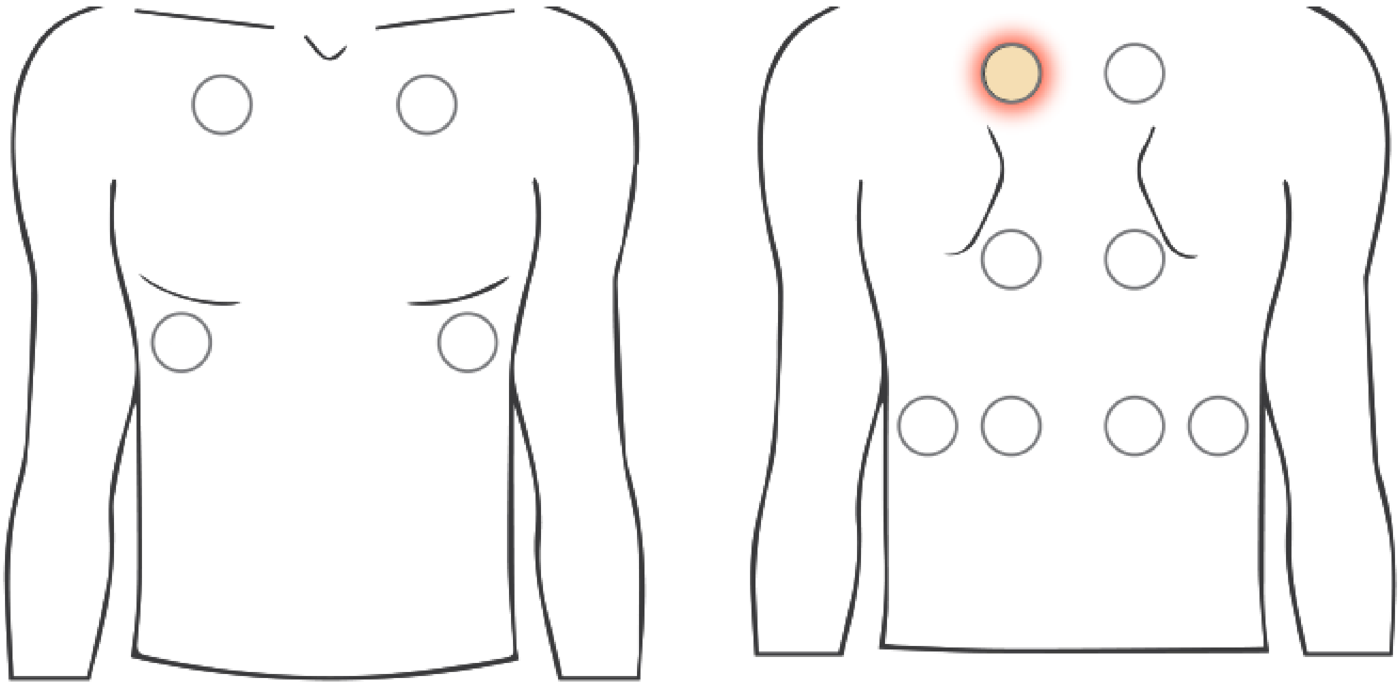

- ☐ vesicular breath sound
- ☐ louder breath sound
- ☐ abnormal bronchial sound
- ☐ medium crackles
- ☐ crepitus
- ☐ expiratory wheezes
- ☐ prolonged expiratory phase
- ☐ pleural rub
- ☐ comments
- ☐ diminished breath sound
- ☐ normal bronchial sound
- ☐ fine crackles
- ☐ coarse crackles
- ☐ inspiratory wheezes
- ☐ stridor
- ☐ squawk
- ☐ rhonchi

*

Patient 15

Age: 25 years old
Height: 176 cm
Weight: 58 kg

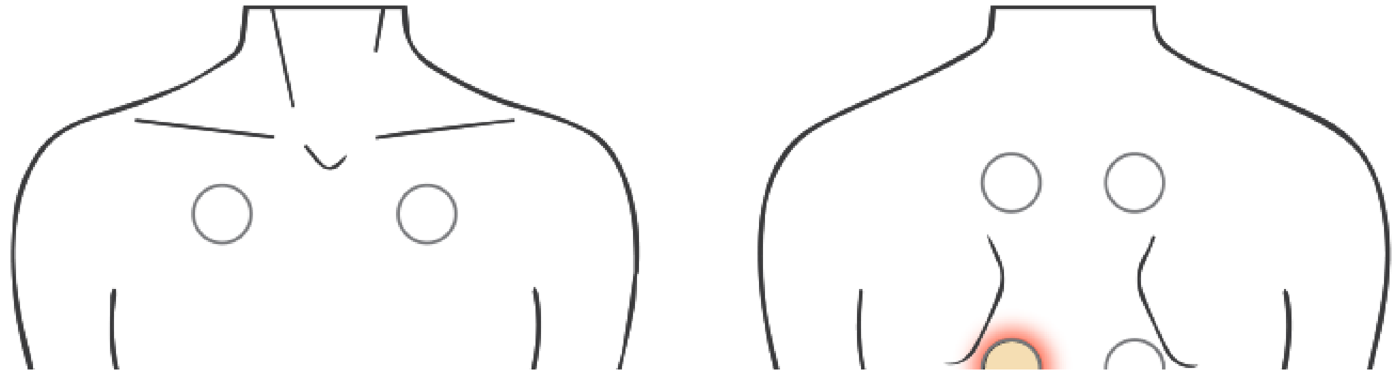

QuestionPro

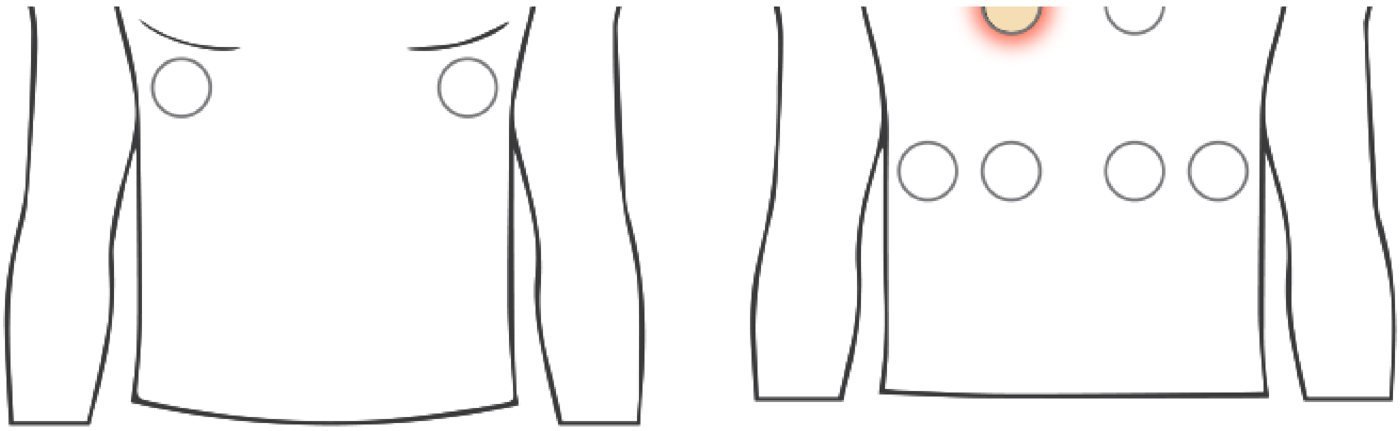

- ☐ vesicular breath sound
- ☐ louder breath sound
- ☐ abnormal bronchial sound
- ☐ medium crackles
- ☐ crepitus
- ☐ expiratory wheezes
- ☐ prolonged expiratory phase
- ☐ pleural rub
- ☐ comments
- ☐ diminished breath sound
- ☐ normal bronchial sound
- ☐ fine crackles
- ☐ coarse crackles
- ☐ inspiratory wheezes
- ☐ stridor
- ☐ squawk
- ☐ rhonchi

*

Patient 16

Age: 3 years old

Height: 94 cm

Weight: 14 kg

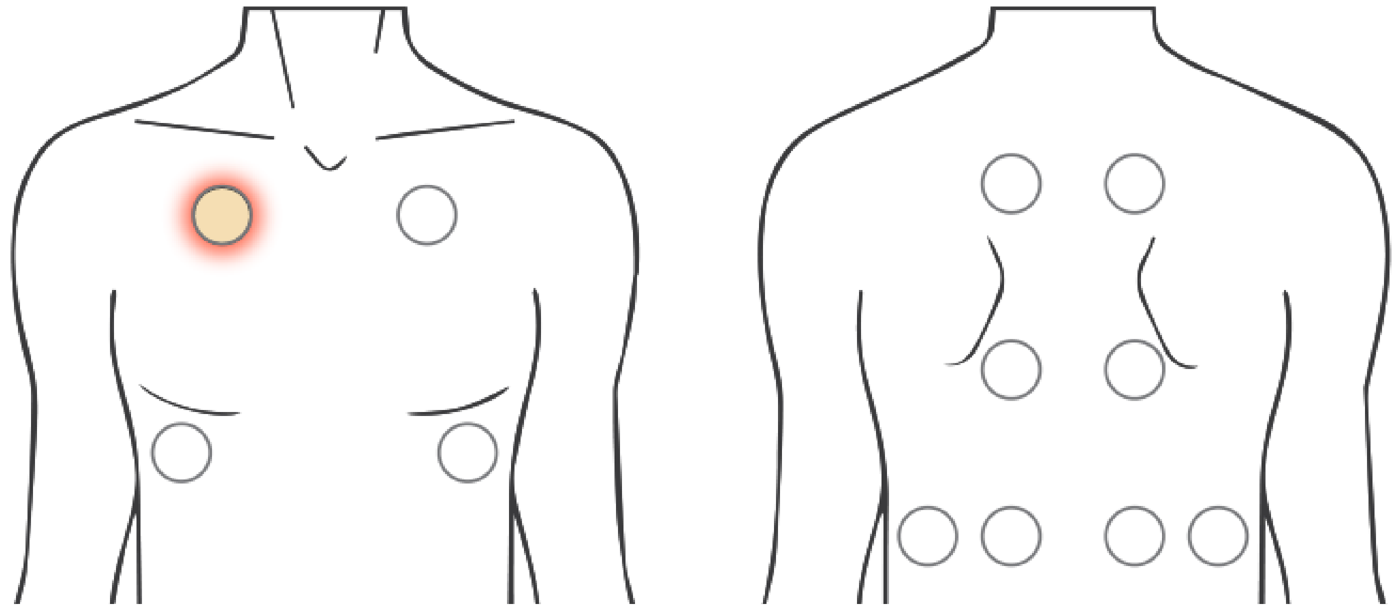

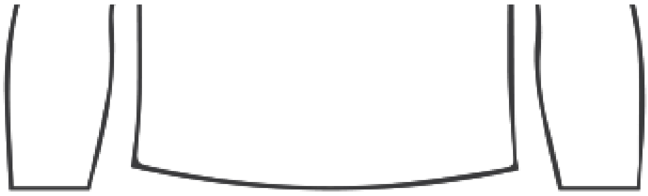

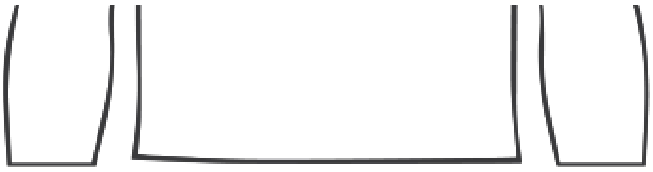

- ☐ vesicular breath sound
- ☐ louder breath sound
- ☐ abnormal bronchial sound
- ☐ medium crackles
- ☐ crepitus
- ☐ expiratory wheezes
- ☐ prolonged expiratory phase
- ☐ pleural rub
- ☐ comments
- ☐ diminished breath sound
- ☐ normal bronchial sound
- ☐ fine crackles
- ☐ coarse crackles
- ☐ inspiratory wheezes
- ☐ stridor
- ☐ squawk
- ☐ rhonchi

*

Patient 17

Age: 17 years old
Height: 150 cm
Weight: 50 kg

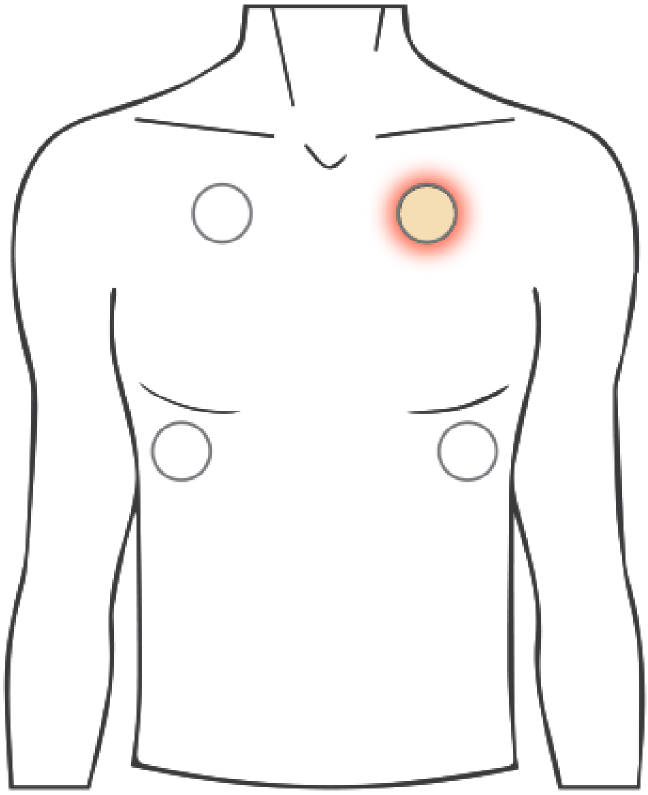

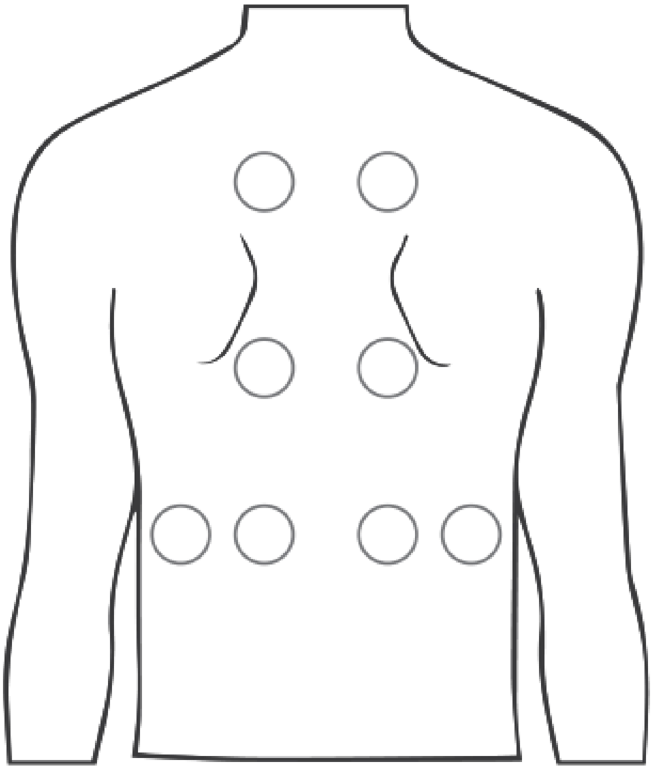

- ☐ vesicular breath sound
- ☐ louder breath sound
- ☐ abnormal bronchial sound
- ☐ medium crackles
- ☐ crepitus
- ☐ expiratory wheezes
- ☐ prolonged expiratory phase
- ☐ pleural rub
- ☐ comments
- ☐ diminished breath sound
- ☐ normal bronchial sound
- ☐ fine crackles
- ☐ coarse crackles
- ☐ inspiratory wheezes
- ☐ stridor
- ☐ squawk
- ☐ rhonchi

*

Patient 18
Age: 18 years old
Height: 179 cm
Weight: 60 kg

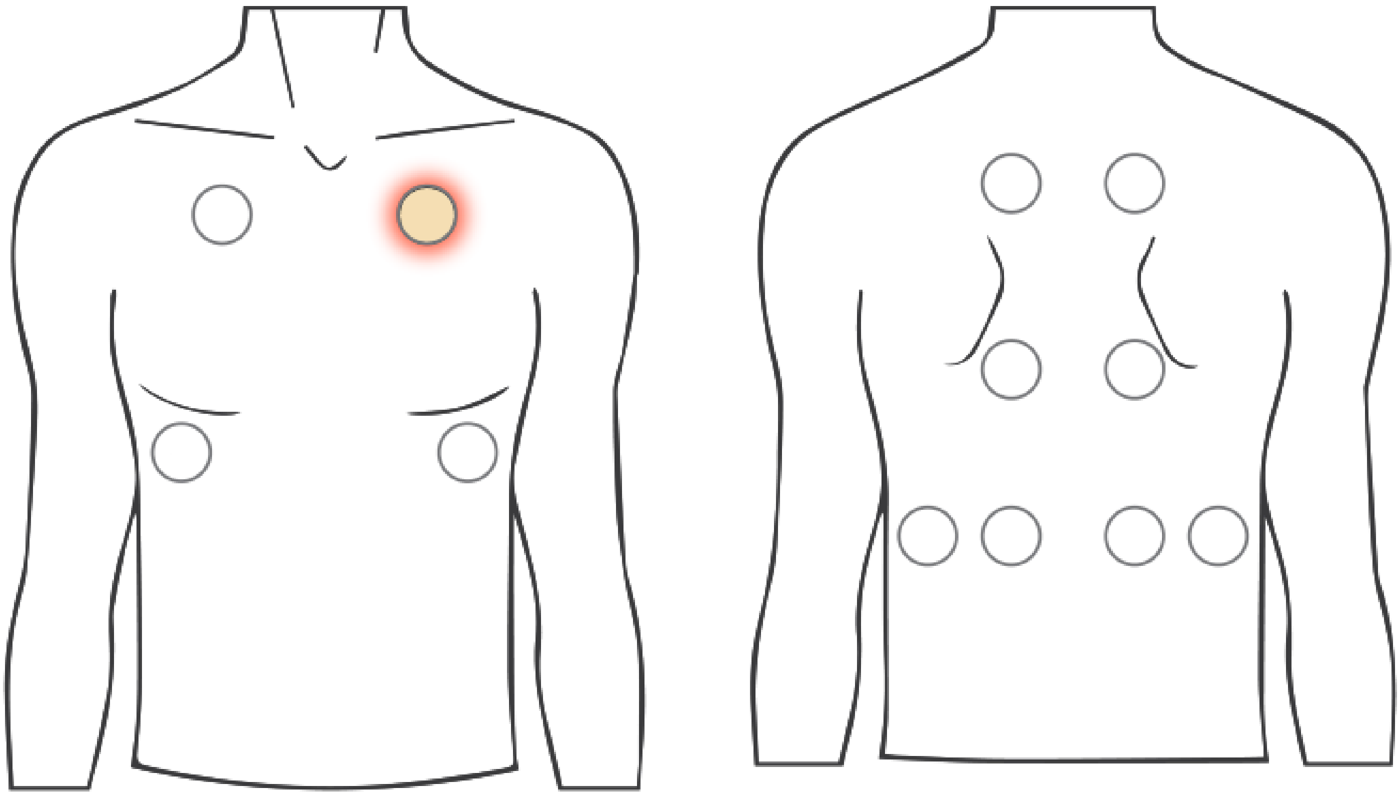

- ☐ vesicular breath sound
- ☐ louder breath sound
- ☐ abnormal bronchial sound
- ☐ medium crackles
- ☐ diminished breath sound
- ☐ normal bronchial sound
- ☐ fine crackles
- ☐ coarse crackles

- [ ] crepitus
- [ ] inspiratory wheezes
- [ ] expiratory wheezes
- [ ] stridor
- [ ] prolonged expiratory phase
- [ ] squawk
- [ ] pleural rub
- [ ] rhonchi
- [ ] comments

*

Patient 19

Age: 2 years old

Height: 84 cm
Weight: 11 kg

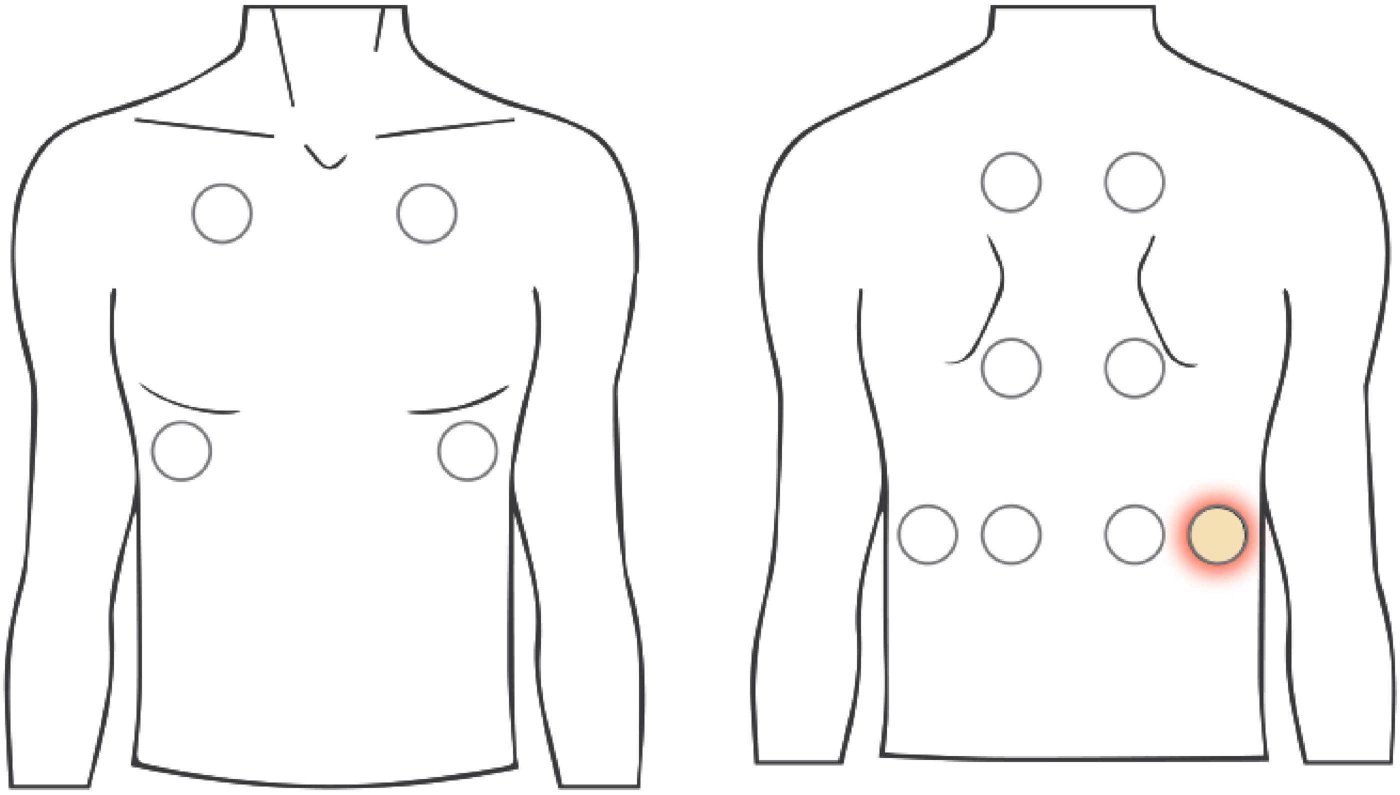

- [ ] vesicular breath sound
- [ ] diminished breath sound
- [ ] louder breath sound
- [ ] normal bronchial sound
- [ ] abnormal bronchial sound
- [ ] fine crackles

QuestionPro

☐ medium crackles

☐ coarse crackles

☐ crepitus

☐ inspiratory wheezes

☐ expiratory wheezes

☐ stridor

☐ prolonged expiratory phase

☐ squawk

☐ pleural rub

☐ rhonchi

☐ comments

*

Patient 20
Age: 6 years old
Height: 118 cm
Weight: 23 kg

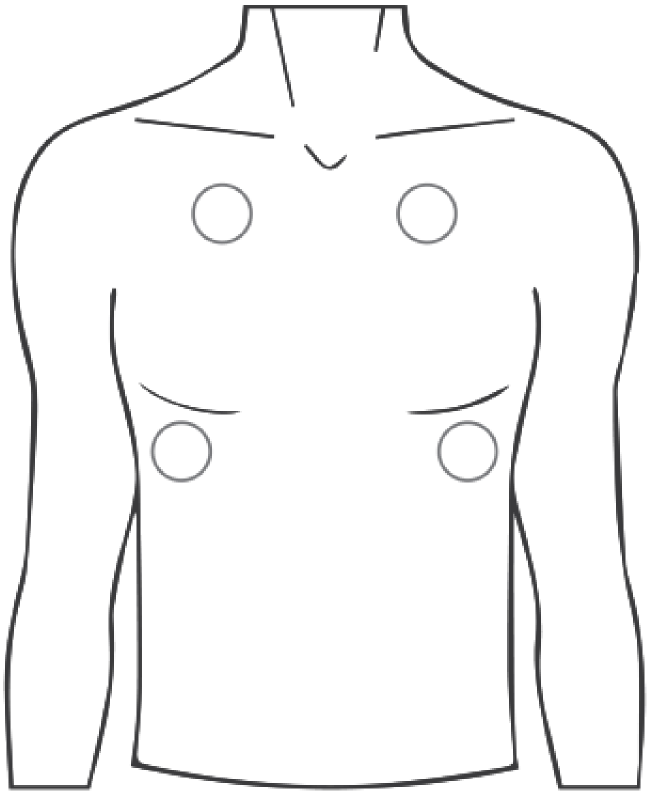

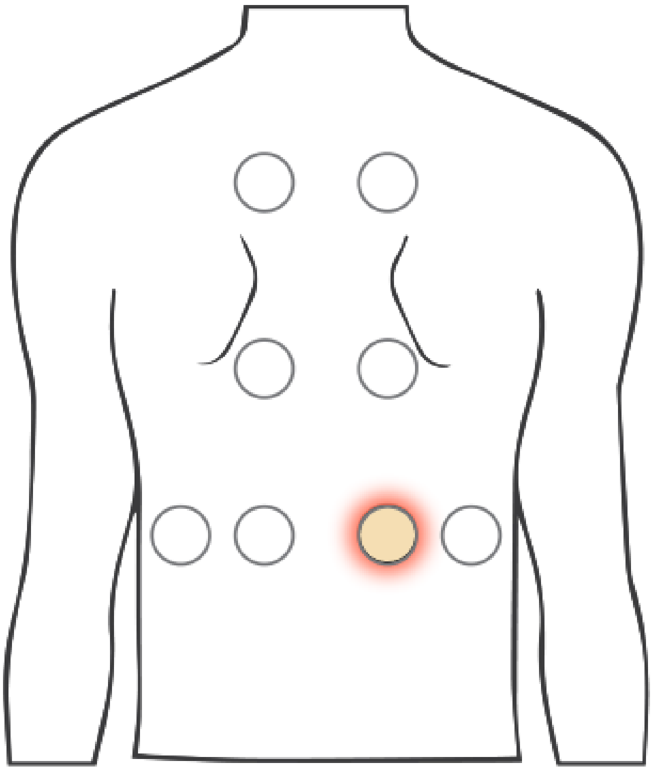

☐ vesicular breath sound

☐ diminished breath sound

☐ louder breath sound

☐ normal bronchial sound

☐ abnormal bronchial sound

☐ fine crackles

☐ medium crackles

☐ coarse crackles

☐ crepitus

☐ inspiratory wheezes

☐ expiratory wheezes

☐ stridor

☐ prolonged expiratory phase

☐ squawk

☐ pleural rub

☐ rhonchi

☐ comments

*

Patient 21

Age: 35 years old
Height: 180 cm
Weight: 74 kg

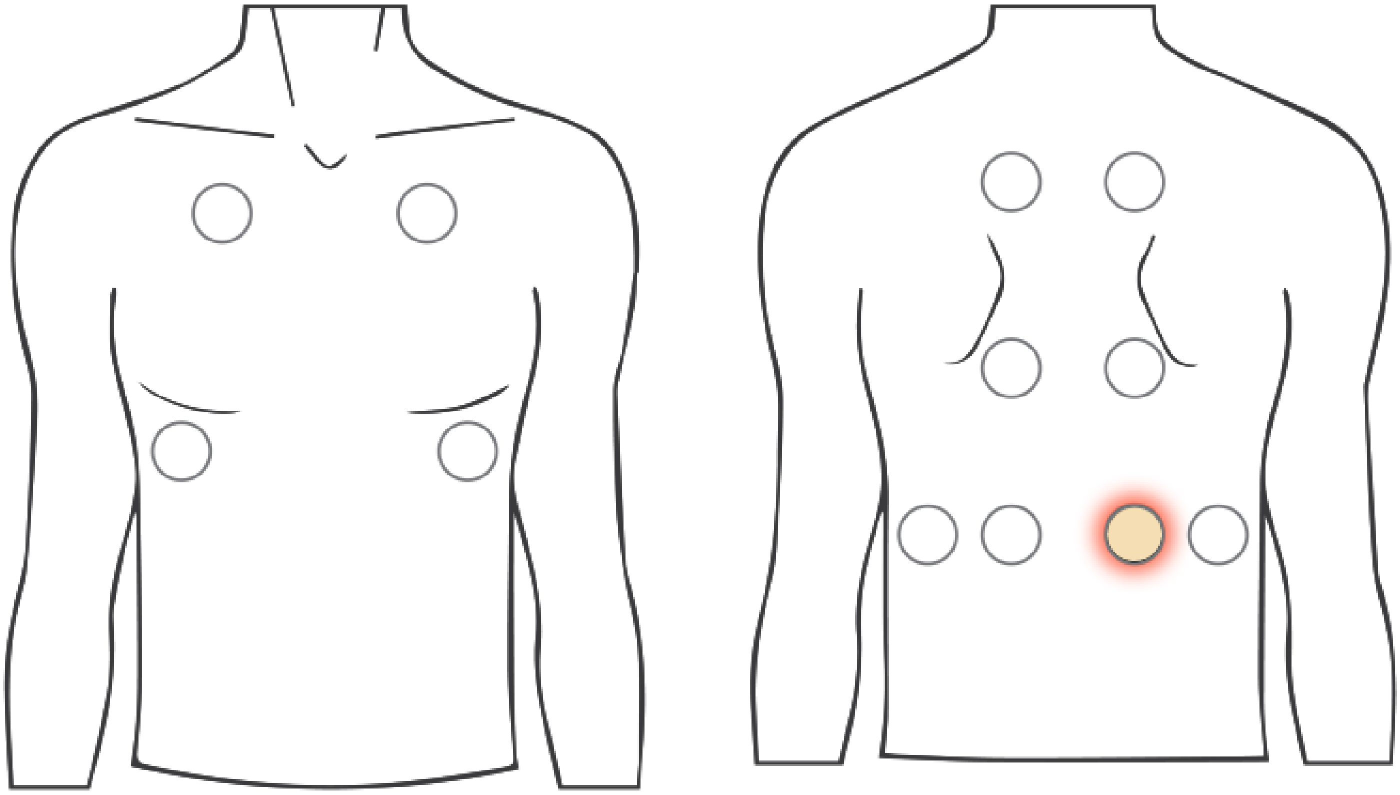

☐ vesicular breath sound

☐ diminished breath sound

☐ louder breath sound

☐ normal bronchial sound

☐ abnormal bronchial sound

☐ fine crackles

☐ medium crackles

☐ coarse crackles

☐ crepitus

☐ inspiratory wheezes

☐ expiratory wheezes

☐ stridor

☐ prolonged expiratory phase

☐ squawk

☐ pleural rub

☐ rhonchi

☐ comments

*

Patient 22
Age: 56 years old
Height: 162 cm
Weight: 53 kg

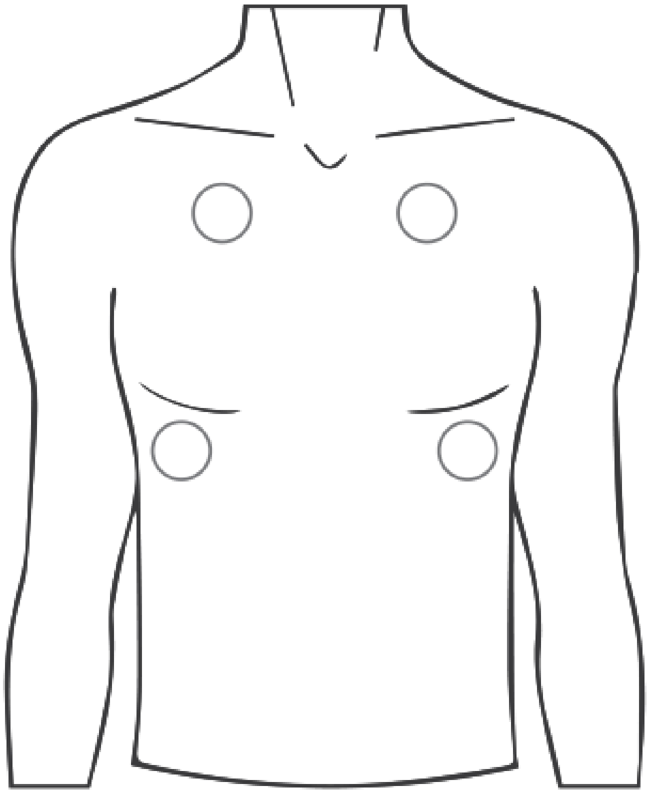

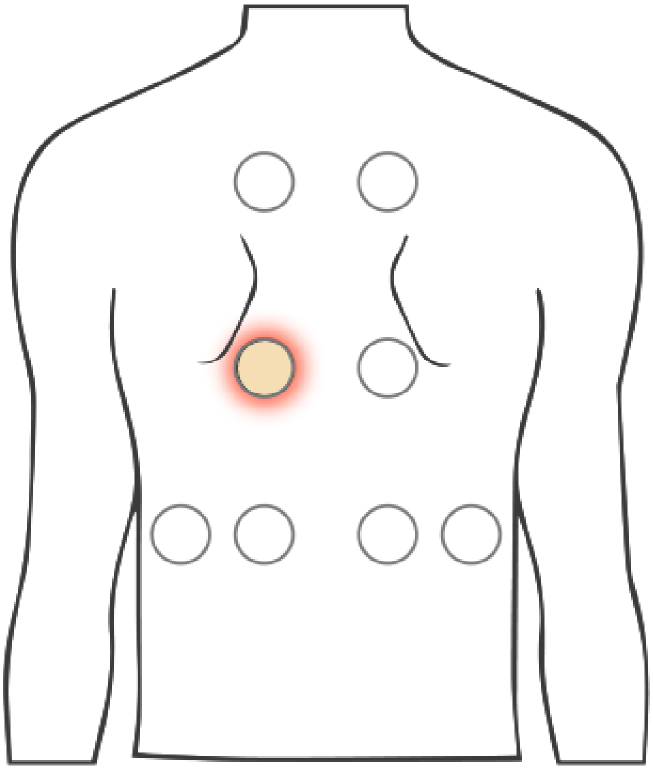

- ☐ vesicular breath sound
- ☐ louder breath sound
- ☐ abnormal bronchial sound
- ☐ medium crackles
- ☐ crepitus
- ☐ expiratory wheezes
- ☐ prolonged expiratory phase
- ☐ pleural rub
- ☐ comments
- ☐ diminished breath sound
- ☐ normal bronchial sound
- ☐ fine crackles
- ☐ coarse crackles
- ☐ inspiratory wheezes
- ☐ stridor
- ☐ squawk
- ☐ rhonchi

*
Patient 23
Age: 11 years old
Height: 147 cm
Weight: 32 kg

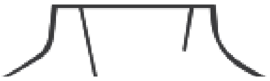

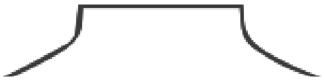

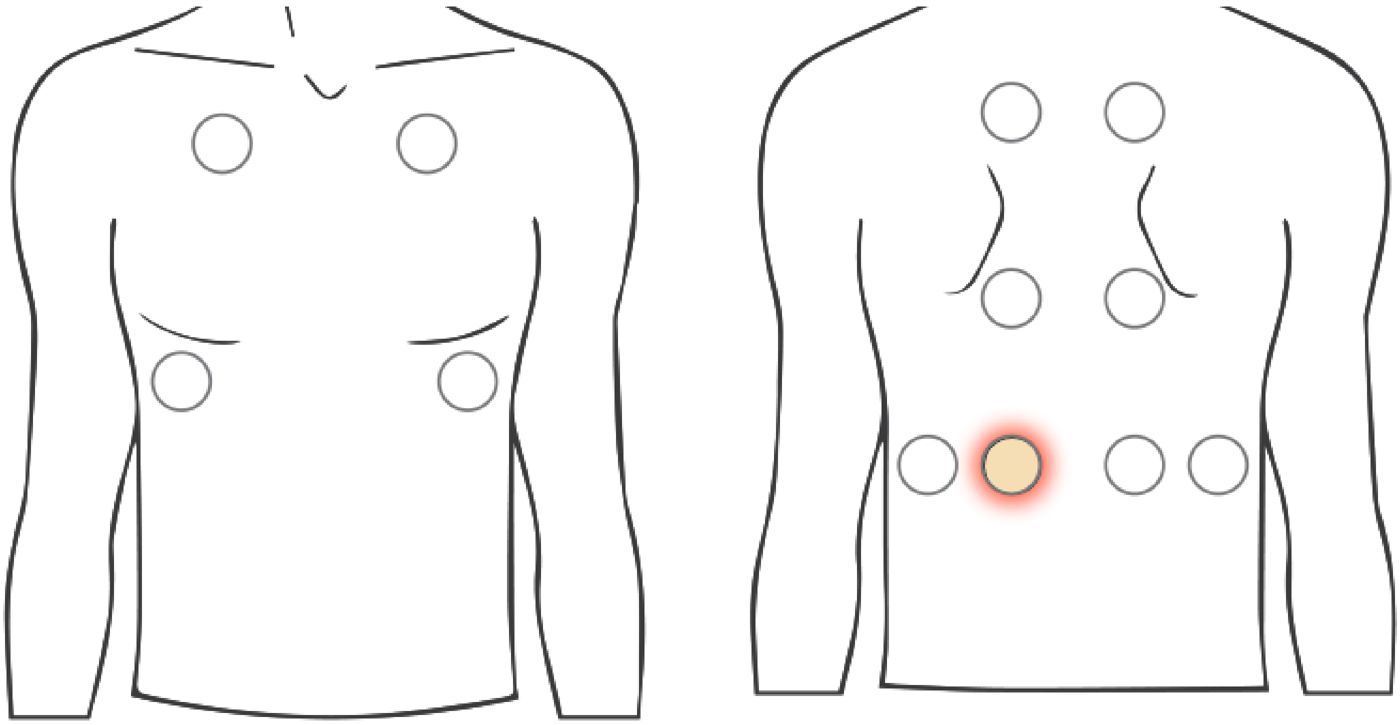

- ☐ vesicular breath sound
- ☐ louder breath sound
- ☐ abnormal bronchial sound
- ☐ medium crackles
- ☐ crepitus
- ☐ expiratory wheezes
- ☐ prolonged expiratory phase
- ☐ pleural rub
- ☐ comments
- ☐ diminished breath sound
- ☐ normal bronchial sound
- ☐ fine crackles
- ☐ coarse crackles
- ☐ inspiratory wheezes
- ☐ stridor
- ☐ squawk
- ☐ rhonchi

*

Patient 24

Age: 14 years old
Height: 150 cm
Weight: 35 kg

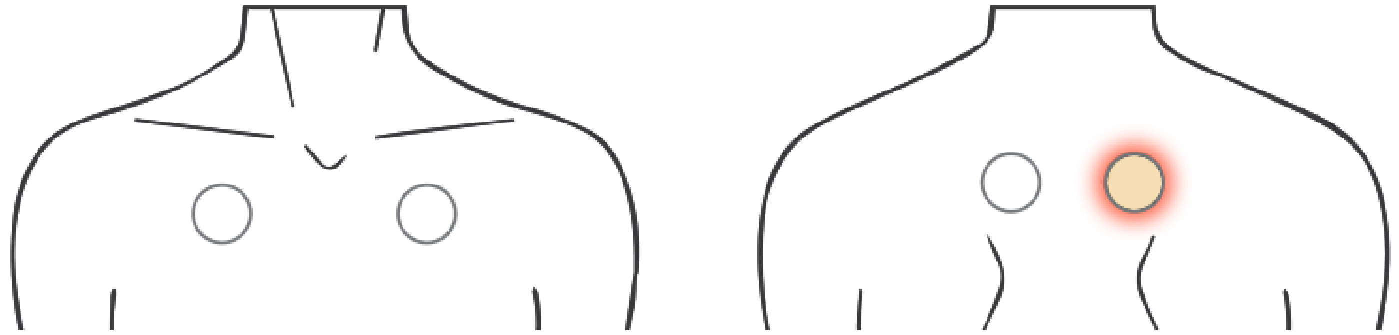

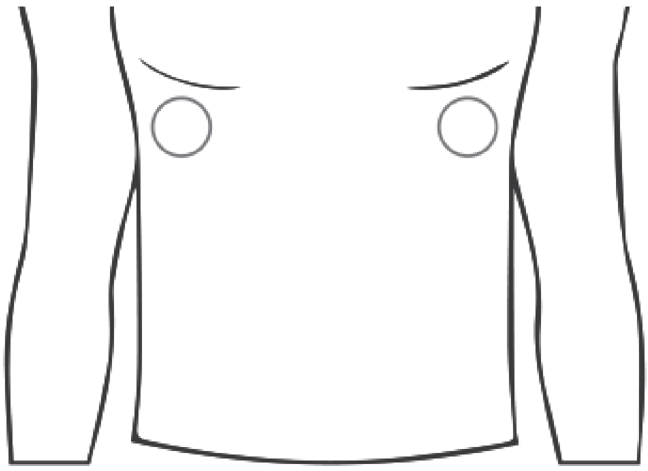

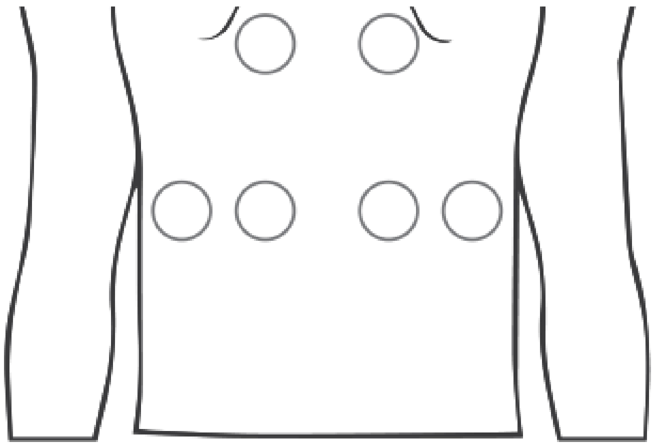

- ☐ vesicular breath sound
- ☐ louder breath sound
- ☐ abnormal bronchial sound
- ☐ medium crackles
- ☐ crepitus
- ☐ expiratory wheezes
- ☐ prolonged expiratory phase
- ☐ pleural rub
- ☐ comments
- ☐ diminished breath sound
- ☐ normal bronchial sound
- ☐ fine crackles
- ☐ coarse crackles
- ☐ inspiratory wheezes
- ☐ stridor
- ☐ squawk
- ☐ rhonchi

*** What kind of headphones do you use to listen to the recordings? If you know the name of the headphones company, please enter in the box**

- ☐ Over-Ear Headphones
- ☐ In-Ear Headphones
- ☐ Name of the headphones company
